# High Diversity, Low Disparity and Small Body Size in Plesiosaurs (Reptilia, Sauropterygia) from the Triassic–Jurassic Boundary

**DOI:** 10.1371/journal.pone.0031838

**Published:** 2012-03-16

**Authors:** Roger B. J. Benson, Mark Evans, Patrick S. Druckenmiller

**Affiliations:** 1 Department of Earth Sciences, University of Cambridge, Cambridge, United Kingdom; 2 Department of Earth Sciences, University College London, London, United Kingdom; 3 New Walk Museum and Art Gallery, Leicester, United Kingdom; 4 Department of Geology, University of Leicester, Leicester, United Kingdom; 5 University of Alaska Museum and Department of Geology and Geophysics, University of Alaska Fairbanks, Fairbanks, Alaska, United States of America; Institut de Biologia Evolutiva – Universitat Pompeu Fabra, Spain

## Abstract

Invasion of the open ocean by tetrapods represents a major evolutionary transition that occurred independently in cetaceans, mosasauroids, chelonioids (sea turtles), ichthyosaurs and plesiosaurs. Plesiosaurian reptiles invaded pelagic ocean environments immediately following the Late Triassic extinctions. This diversification is recorded by three intensively-sampled European fossil faunas, spanning 20 million years (Ma). These provide an unparalleled opportunity to document changes in key macroevolutionary parameters associated with secondary adaptation to pelagic life in tetrapods. A comprehensive assessment focuses on the oldest fauna, from the Blue Lias Formation of Street, and nearby localities, in Somerset, UK (Earliest Jurassic: 200 Ma), identifying three new species representing two small-bodied rhomaleosaurids (*Stratesaurus taylori* gen et sp. nov.; *Avalonnectes arturi* gen. et sp. nov) and the most basal plesiosauroid, *Eoplesiosaurus antiquior* gen. et sp. nov. The initial radiation of plesiosaurs was characterised by high, but short-lived, diversity of an archaic clade, Rhomaleosauridae. Representatives of this initial radiation were replaced by derived, neoplesiosaurian plesiosaurs at small-medium body sizes during a more gradual accumulation of morphological disparity. This gradualistic modality suggests that adaptive radiations within tetrapod subclades are not always characterised by the initially high levels of disparity observed in the Paleozoic origins of major metazoan body plans, or in the origin of tetrapods. High rhomaleosaurid diversity immediately following the Triassic-Jurassic boundary supports the gradual model of Late Triassic extinctions, mostly predating the boundary itself. Increase in both maximum and minimum body length early in plesiosaurian history suggests a driven evolutionary trend. However, Maximum-likelihood models suggest only passive expansion into higher body size categories.

## Introduction

The origin of Plesiosauria 200 million years ago (Ma) was a landmark event in tetrapod evolution. Spanning 135 Ma, plesiosaurians represent one of only three long-lived radiations of secondarily marine, non-mammalian tetrapods (alongside ichthyosaurs and marine turtles; duration >100 Ma). Plesiosaurians possess an unusual body plan not seen in other marine vertebrates [1]. All four limbs are enlarged and modified as propulsive flippers, the trunk is short and stiff, and proportional head size seems to vary inversely with neck length, resulting in a gradation of forms between ‘plesiosauromorph’ (long neck, small head) and ‘pliosauromorph’ (short neck, large head) extremes [2]–[3]. Despite its oddity, this body plan was an extraordinarily successful adaptation to life in the open ocean [1], [4]. It secured plesiosaurian survival for the entire Mesozoic, in spite of the emergence of possibly competing marine tetrapods clades [5], and recurrent regression events, which decimated shallow marine tetrapod lineages [6], including basal representatives of Sauropterygia, the wider clade that includes Plesiosauria.

Despite its significance for understanding the macroevolutionary dynamics of adaptive radiations, the fossil record of the oldest plesiosaurs has been relatively understudied. This contrasts with early records of other pelagic tetrapod clades, the ichthyosaurs [7]–[10] and cetaceans [11]–[14]. Lower Jurassic (200–175 Ma) European deposits yield the earliest plesiosaurian fossils, possessing their full complement of pelagic adaptations. Abundant specimens provide snapshots of the emergence of Plesiosauria in the earliest Hettangian of Somerset and Leicestershire, UK (∼200 million years ago [Mya] [15]–[16]), the Sinemurian of Dorset and Leicestershire UK (∼197–190 Mya [17]–[18]) and the lower Toarcian of Yorkshire and Northamptonshire, UK, and Baden-Württemberg, Germany (∼183–180 Mya [19]–[20]). The occurrence of multiple intensively-sampled horizons provides unique data on key biotic and ecological parameters associated with invasion of the open ocean, including body size, species richness, and morphological disparity. Furthermore, global ecosystems were in decline for much of the Late Triassic, resulting in a progressive diminution of diversity in many invertebrate clades reviewed by [21], and possibly culminating in Late Triassic extinctions, or a Triassic-Jurassic boundary extinction event (e.g. [22]). The exact cause and modality of these extinctions is uncertain [21]–[22]. However, the Lower Jurassic rise of plesiosaurians is one aspect of global recovery from this episode.

We present the results of a comprehensive review of the little-studied earliest Jurassic plesiosaurian fauna from Street, Somerset, UK (and an adjacent, contemporaneous locality at Watchet, Somerset), and a new phylogenetic dataset focussed on Lower Jurassic taxa. These are used as tools to study the earliest stage of plesiosaurian evolution.

### Plesiosaurian faunal composition at Street

The Blue Lias Formation at Street, Somerset, UK was extensively quarried for building stone in the 19^th^ century [16], [23]–[25], resulting in the discovery of 25 extant plesiosaur fossils [16]–[17], [26]–[30]. Most specimens likely originate from the Pre-*planorbis* beds, which occur below the first occurrence of the ammonite *Psiloceras planorbis*. Thus, they probably fall within the earliest Hettangian *P. tilmanni* Chronozone, immediately following the Triassic–Jurassic boundary [31], although some specimens may be from slightly younger horizons [16]. These specimens are usually considered to represent three taxa [16]: one individual each of the large-bodied rhomaleosaurids *Eurycleidus arcuatus* (Owen, 1840) [32] and ‘*Rhomaleosaurus*’ *megacephalus* (Stutchbury, 1846) [33], plus 23 specimens of smaller-bodied individuals (trunk length<1 metre) that are typically referred to the basal pliosaurid *Thalassiodracon hawkinsii* (Owen, 1838) [34]. However, Benson *et al*. [35] listed a number of morphologically distinct specimens and suggested that ‘*Plesiosaurus*’ *cliduchus* Seeley, 1865a [29] also represented a distinct, valid taxon.

Our taxonomic revision of the plesiosaurian fauna from Street indicates the presence of six species, including two new taxa identified here for the first time (below, *Systematic Palaeontology*). In total 16 plesiosaurian specimens from Street are taxonomically determinate (Table 1; nine additional specimens are indeterminate). Penecontemporaneous strata at Watchet, Somerset yield an additional new taxon (below; *Systematic Palaeontology*). Other localities of similar age at ‘Street-on-the-Fosse’ (Pylle), Somerset and Barrow-on-Soar, Leicestershire have yielded additional specimens of ‘*Rhomaleosaurus*’ *megacephalus* [15]–[16], a specimen of *T. hawkinsii* is also known from Walton, Somerset [35].

**Table 1:** Revised taxonomy of plesiosaurian specimens from the earliest Jurassic of Street, Somerset, UK, and Watchet (TTNCM 8348) (modified from [35], table 1). Abbreviation: NMING, National Museum of Ireland, Dublin, Ireland.

Specimen	References and notes
*Thalassiodracon hawkinsii*	
NHMUK 2018*	Lectotype: skull and skeleton
NHMUK 2020*[14551]	Skull with partial postcranial skeleton
NHMUK 2022*[14549]	Skull and skeleton
ANSP 15767	Skull and skeleton
CAMSM J.35181	Partial postcranial skeleton
CAMSM J.46986	Skull, anterior cervical vertebrae and fragments
GSM 51235	Skull and skeleton
*Eurycleidus arcuatus*	
NHMUK 2030*	Lectotype: partial mandible. Disarticulated postcranial remains (NHMUK R1317–9, 2027*–2030*, 2047*, 2061*) probably represent the same individual as the lectotype (Cruickshank 1994)
*Rhomaleosaurus megacephalus*	
NMING F10194	Partial postcranial skeleton
‘*Plesiosaurus*’ *cliduchus*	
CAMSM J.35180	Holotype: partial postcranial skeleton
*Stratesaurus taylori* n. gen. et sp.	
OUMNH J.10337	Skull, and partial postcranial skeleton
AGT 11	Skull
GSM 26035	Skull and anterior cervical vertebrae
*Avalonnectes arturi* n. gen. et sp.	
NHMUK 14550	Partial skull and postcranial skeleton
AGT uncatalogued	Partial postcranial skeleton
*Eoplesiosaurus antiquior* n. gen. et sp.	
TTNCM 8348	Postcranial skeleton – cannot be compared to ‘*Plesiosaurus*’ *cliduchus*.
Not determined	
MANCH MM L.9767	Fragmentary postcranium
NHMUK R45	Not determined
NHMUK R1331	Limb
NHMUK 2039*	Mandible
OUMNH J.10327	Partial postcranial skeleton.
RM 4110	Postcranial and cranial fragments.
SWM uncatalogued	Not examined.
TTNCM 8345	Not examined
TTNCM 9291	Skull without mandible; tentatively referred to new taxon B.
UCD uncat.	Not examined.

The presence of seven species in the lowermost Hettangian indicates high taxic diversity at a single locality immediately following the Triassic–Jurassic boundary. This is higher than the number of species known from the Late Hettangian–Sinemurian of Lyme Regis and Charmouth, Dorset and the lower Toarcian of Holzmaden, Germany and vicinity (both localities have yielded five species; Appendix S1); it is lower than the number of species known from the lower Toarcian of the UK (eight species; Appendix S1), and from the lower Toarcian of Europe (Germany and the UK combined: 13 species; Appendix S1). However, Toarcian plesiosaur faunas contain multispecific genera that inflate species counts, and the number of genera known from the lower Toarcian of Europe is in fact comparable to that from Street (seven genera).

### Nomenclatural Acts

The electronic version of this document does not represent a published work according to the International Code of Zoological Nomenclature (ICZN), and hence the nomenclatural acts contained in the electronic version are not available under that Code from the electronic edition. Therefore, a separate edition of this document was produced by a method that assures numerous identical and durable copies, and those copies were simultaneously obtainable (from the publication date noted on the first page of this article) for the purpose of providing a public and permanent scientific record, in accordance with Article 8.1 of the Code. The separate print-only edition is available on request from PLoS by sending a request to PLoS ONE, 1160 Battery Street, Suite 100, San Francisco, CA 94111, USA along with a check for $10 (to cover printing and postage) payable to “Public Library of Science”. In addition, this published work and the nomenclatural acts it contains have been registered in ZooBank <http://zoobank.org>, the proposed online registration system for the ICZN. The ZooBank LSIDs (Life Science Identifiers) can be resolved and the associated information viewed through any standard web browser by appending the LSID to the prefix “http://zoobank.org/”. The LSID for this publication is: urn:lsid:zoobank.org:pub:5D0B1E45-4D2D-43F7-9480-F85C9832F815.

### Systematic Palaeontology

Pistosauria Baur 1887–1890 [36]

Plesiosauria de Blainville, 1835 [37]

Rhomaleosauridae Nopcsa, 1928 [38]

*Stratesaurus* n. gen.

urn:lsid:zoobank.org:act:2C627698-239B-4436-81E5-E6FA5173F618

*Type and only species. Stratesaurus taylori*

*Diagnosis*. As for the type and only species.

*Stratesaurus taylori* n. gen. et sp.

urn:lsid:zoobank.org:act:E2BA4E44-3E28-4987-928F-DF559BDCA98F

1822 *Plesiosaurus* sp. de la Beche and Conybeare; Conybeare 1822[39]:pl. 19

1996 *Thalassiodracon hawkinsi* Owen; Storrs and Taylor 1996[16]:404

2001 *Thalassiodracon hawkinsi* Owen; O'Keefe 2001[40]:fig. 4

#### Holotype

**Figure 1:**
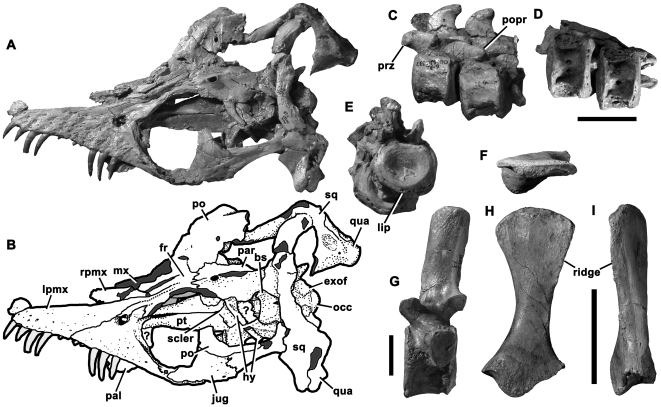
Holotype of *Stratesaurus taylori* (OUMNH J.10337). **A–B**, skull in dorsal view, **C–E**, anterior cervical vertebrae in left lateral (**C**), ventral (**D**) and anterior (**E**) views, **F**, **H–I**, left ilium in dorsal (**F**), lateral (**H**) and posterior (**I**) views, **G**, ‘pectoral’ vertebra; in left lateral view. In line drawing (**B**), grey tone indicates damage. Abbreviations: bs, basisphenoid; exof, exoccipital facet of basioccipital; jug, jugal; fr, frontal; hy, hyoid; lpmx, left premaxilla; mx, maxilla; occ, occipital condyle; pal, palatine; par, parietal; po, postorbital; popr, posterolateral process of prezygapophysis; prz, prezygapophysis; pt, pterygoid; qua, quadrate; rmx, right maxilla; rpmx, right premaxilla; scler, sclerotic ring; sq, squamosal. Scale bars equal 50 mm (**A–B**, **F**, **H–I**) and 20 mm (**C–E**, **G**).

OUMNH (Oxford University Museum of Natural History, Oxford, UK) J.10337, a skull and partial postcranial skeleton including anterior cervical and pectoral vertebrae, a partial hindlimb and ilium (Fig. 1) from Street, Somerset, UK (likely lowermost Hettangian; see above; *Plesiosaurian faunal composition at Street*).

#### Etymology

Genus name from ‘Strate’, the name for Street recorded in the Doomesday Book and *sauros*, Greek meaning lizard. Specific epithet after Michael A. Taylor, who carried out acid preparation of OUMNH J.10337 during an earlier study of Street plesiosaurians [16].

#### Referred specimens

AGT (Alfred Gillett Trust, Street, UK) 11, a skull, and GSM 26035 ([39], pl. 19), a skull with anterior cervical vertebrae. GSM 26035 possesses prominent posterior processes on the posterolateral surfaces of the prezygapophyses, an autapomorphy of *S. taylori* (below; *Diagnosis*). AGT 11 is difficult to distinguish from *Avalonnectes arturi* (below), for which only the postorbital skull is known. However, in AGT 11, GSM 26035, and probably OUMNH J.10337, the premaxilla terminates posteriorly at approximately orbital midlength, whereas it extends further posteriorly in *A. arturi*. Furthermore, in AGT 11 and OUMNH J.10337 the squamosals contact each other dorsal to the parietals, unlike in *A. arturi*, in which this contact is more posterior. AGT 11 is otherwise indistinguishable from OUMNH J.10337 and GSM 26035 and is thus referred to *S. taylori*.

#### Diagnosis

Small-bodied basal plesiosaurian (skull of holotype 180 mm long) with five premaxillary and 16 maxillary alveoli, lacking rostral constriction. Possesses two autapomorphies: prominent posterior processes on posterolateral surfaces of anterior cervical prezygapophyses; pectoral centra proportionally short (length:anterior height ratio = 0.7).

#### Short description of holotype

The skull is crushed dorsoventrally, but has been prepared using acid and is almost free of matrix (Fig. 1A, B). It shows many details that are only briefly described here. The snout tapers anteriorly and lacks a rostral constriction. Thus, the premaxilla is not transversely expanded, unlike many robust Lower Jurassic taxa, including ‘*Rhomaleosaurus*’ *megacephalus* [15]. The premaxilla contains five teeth of subequal size, as in many basal plesiosaurs, but unlike *T. hawkinsii*, which has four premaxillary teeth [35]. The maxilla contains 16 weakly heterodont alveoli that terminate ventral to the postorbital bar. A superficial flange of the maxilla extends dorsally posterior to the external naris (Fig. 1A, B). This was figured as a distinct ossification contacting the naris, and identified as a nasal, by O'Keefe ([40], fig. 4). However, it does not contact the external naris, and we interpret the suture at the base of the ‘nasal’ (of O'Keefe [40]) as a crack. The parietal bears a low sagittal crest posterior to the suboval pineal foramen. The posterior part of the parietal is abruptly expanded laterally, forming the ‘lateral angle’ of Smith & Dyke [41] that is present in rhomaleosaurids and more basal pistosaurians (e.g. [42]). A dorsoventrally thin, sheet-like anterior extension of the squamosals overlaps the posterior surface of the parietals. A rounded squamosal bulb is present and the squamosals contact one another dorsal to the parietals. The posteroventral process of the postorbital extends far posteriorly, forming the dorsal margin of the temporal bar for most of its length (Fig. 1A–B). This also occurs in basal sauropterygians [42]–[43] and rhomaleosaurids (e.g. *R. thorntoni*: Natural History Museum, London, UK [NHMUK] R4853). Contrastingly, in pliosaurids and plesiosauroids the length of the posteroventral process is reduced [35], [44].

Eighteen anterior-middle cervical vertebrae are preserved, including most of the atlas-axis complex. The neurocentral sutures are convex, as in *E. antiquior* n. sp. (below), but unlike the other taxa present at Street. The cervical centra have double-headed rib facets and concave anterior and posterior surfaces that are approximately 1.2 times as broad mediolaterally as high dorsoventrally (Fig. 1C, D, E). A small ‘lip’ projects ventrally from the anterior surface (Fig. 1E). This has been considered to be a unique synapomorphy of derived pliosaurids, known from the Middle Jurassic onwards [45]–[46]. However, during the present study it was also observed in a specimen referred to ‘*Plesiosaurus*’ *macrocephalus* Owen, 1840 from the Lower Lias Group of Lyme Regis, Dorset (NHMUK 49202 [47]), which also has transversely broad cervical centra. Uniquely in *S. taylori*, prominent processes project posteriorly from the posterolateral surfaces of the anterior cervical prezygapophyses (Fig. 1C). These are confirmed as absent in most plesiosaurian taxa represented at Street: ‘*R*.’ *megacephalus* (LEICS G221.1851), *E. arcuatus* (NHMUK 2047*), *T. hawkinsii* [16], *Avalonnectes arturi* and *Eoplesiosaurus antiquior* (see below). The condition cannot be determined in ‘*Plesiosaurus*’ *cliduchus*, for which only posterior cervical vertebrae are preserved. However, *S. taylori* has proportionally short pectoral centra (Fig. 1G), unlike all other plesiosaurians from Street, including ‘*P*.’ *cliduchus* (Sedgwick Museum of Earth Sciences, University of Cambridge, Cambridge, UK [CAMSM] J.35180).

The ilium of *S. taylori* is approximately straight in lateral view and has a broadly expanded dorsal end that is rotated approximately 20 degrees relative to the proximal end (Fig. 1F). A rugose, proximodistally oriented ridge is present on the posterolateral surface of the distal expansion (Fig. 1H–I). This is also present in *‘R. megacephalus* (LEICS G221.1851). A complete description of all material referred to *S. taylori* is beyond the scope of the present study but will form the basis of future work.

*Avalonnectes* n. gen.

urn:lsid:zoobank.org:act:027222CA-DA5A-4F63-B59F-63A47567CD93

#### Type and only species

*Avalonnectes arturi*

#### Diagnosis

As for the type and only species.

*Avalonnectes arturi* n. gen. et sp.

urn:lsid:zoobank.org:act:27A8C8C1-5F02-461E-9778-2DB9E2645061

#### Holotype

**Figure 2:**
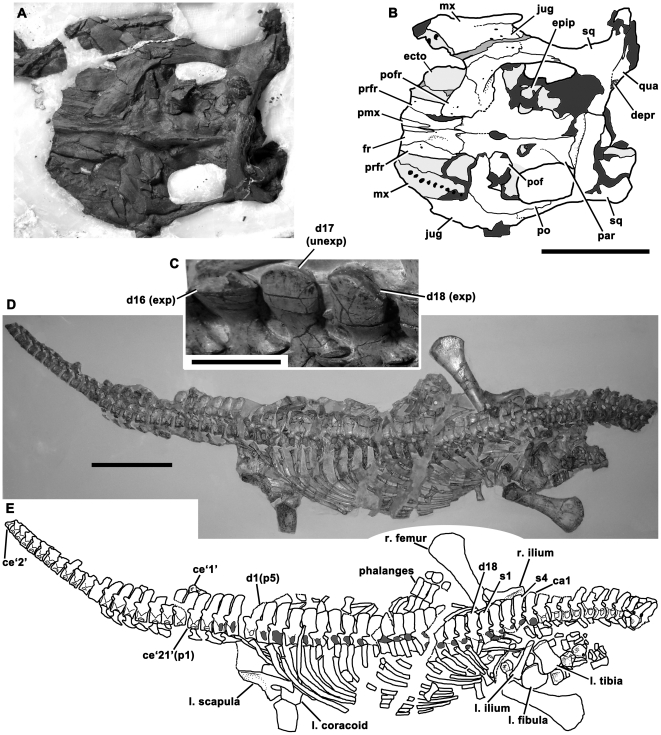
Holotype of *Avalonnectes arturi* (NHMUK 14550). **A–B**, skull in dorsal view; **C–E**, postcranial skeleton; in left dorsolateral (**C**) and left lateral (**D–E**) views. In line drawings (**B**, **E**) dark grey tone indicates damage and light grey tone indicates the palate. Abbreviations: ca, caudal vertebra [number following indicates order in preserved series]; ce, cervical vertebra; d, dorsal vertebra; depr, depression; ecto, ectopterygoid; epip, epipterygoid; exp, expanded neural spine apex; fr, frontal; jug, jugal; l., left [followed by name of element]; mx, maxilla; p, ‘pectoral’ vertebra; par, parietal; pmx, premaxilla; po, postorbital; pofr, postfrontal; prfr, prefrontal; qua, quadrate; r., right [followed by name of element]; s, sacral vertebra; sq, squamosal; unexp, unexpanded neural spine apex. Scale bars equal 50 mm (**A–B**), 20 mm (**C**), and 200 mm (**D–E**).

NHMUK 14550, the posterior portion of the skull, and a partial postcranial skeleton (Fig. 2).

#### Etymology

Genus name from Avalon, an island from the legend of King Arthur, often identified with Glastonbury, near Street, and *nectes*, Greek meaning ‘swimmer’. Species epithet after Arthur Cruickshank (1932–2011), who with M. A. Taylor initiated the restudy of British Lower Jurassic plesiosaurians in the 1990s, and also as a reference to the legendary King Arthur of British folklore.

#### Referred specimen

AGT uncatalogued, a partial postcranial skeleton from Street, Somerset, UK (likely lowermost Hettangian; see above; *Plesiosaurian faunal composition at Street*).

#### Diagnosis

Small-bodied basal rhomaleosaurid (trunk length = 735 mm) with 18–19 dorsal vertebrae, a low, autapomorphic number.

#### Short description of holotype

The postorbital portion of the skull is preserved. It has been embedded in wax so the dorsal surface is exposed (Fig. 2A–B). The tapering posterior processes of the premaxillae extend far posteriorly compared to those of *S. taylori* (Fig. 1A–B), almost to the level of the postorbital bar. However, they do not contact the parietal. The lateral surface of the maxilla is visible on the right side (Fig. 2A–B). It underlaps the jugal and has been displaced posteriorly by crushing. The jugal is penetrated by numerous small foramina and contacts the squamosal just posterior to the postorbital bar. As in *S. taylori* and other basal pistosaurians, the posteroventral process of the postorbital is long, extending more than two-thirds the length of the temporal bar. The postfrontal-postorbital suture is oriented anteroventrally, allowing only a small exposure of the postorbital in the posterior rim of the orbit. The parietal bears a low sagittal crest, which is penetrated anteriorly by a suboval pineal foramen. The posterior part of the parietal is abruptly expanded laterally (Fig. 2 A–B), forming the ‘lateral angle’ that is present in *S. taylori* and other basal pistosaurians. A low squamosal bulb is present.

Twenty-three cervical vertebrae are preserved (Fig. 2D–E). These do not include the atlas-axis complex, so *A. arturi* possessed at least 25 cervical vertebrae. Because of the very small size of the anteriormost preserved centrum (13 mm long anteroposteriorly), it is unlikely that more than one or two additional cervical vertebrae were originally present. Thus, *A. arturi* has an estimated cervical count of 26–28, fewer than in *T. hawkinsii* (exactly 31 cervicals in all four sufficiently-complete referred specimens; [35], table 2), but similar to the number in the larger-bodied ‘*R*.’ *megacephalus* (28 cervicals, trunk length = 1820 mm; New Walk Museum and Art Gallery, Leicester, UK [LEICT] G221.1851). The cervical rib facets have two articular surfaces separated by a narrow horizontal groove. The neurocentral sutures are V-shaped in lateral aspect (Fig. 2D–E), as in *E. arcuatus* (NHMUK 2047*) and ‘*P*.’ *cliduchus* (CAMSM J.35180). This is unlike the condition in *S. taylori* and *Eoplesiosaurus antiquior* (see below), which have rounded neurocentral sutures. It is also unlike the condition in *T. hawkinsii*, in which the neurocentral suture contacts the rib facet (*contra* Benson *et al*. [35]; this also occurs in the Toarcian pliosaurid *Hauffiosaurus* [48]). The posterior cervical neural spines of *A. arturi* are tall, more than 1.4 times the height of the centrum (Fig. 2D–E), as in *E. arcuatus* and ‘*R*.’ *megacephalus*. This is unlike the condition in ‘*P*.’ *cliduchus*, *S. taylori* and *T. hawkinsii*, in which the posterior cervical neural spines are subequal to the centrum height. The holotype of *A. arturi* has only 18 dorsal vertebrae (identified on the basis of rib morphology [35]), and 19 are present in the referred specimen (AGT uncatalogued). This count is low compared to *T. hawkinsii* (22 dorsals [35]) and ‘*R*.’ *megacephalus* (22–23 dorsals; LEICT G221.1851). The complete dorsal series is not known in other plesiosaurians represented at Street. The apices of the dorsal neural spines of *A. arturi* alternate in morphology between being transversely thin and sheet-like, and transversely thicker (Fig. 2C). This was also observed in *E. arcuatus*, and some rhomaleosaurids, including *Meyerasaurus victor* ([49], pl. 10), and ‘*R*.’ *megacephalus* (LEICT G221.1851) during the present study. A qualitatively different alternating morphology was reported in leptocleidid plesiosaurians [50].

**Table 2:** Results of maximum likelhood model fitting of Lower Jurassic plesiosaurian trunk length evolution.

Model	Parameters	AICc	AICc weight	Parameter estimates
Trunk length/mm				
Stasis	2	2932	<0.001	Trait mean = 1381; trait variance = 100
Brownian motion (BM)	2	2363	<0.001	Step variance = 20.0
Brownian motion+trend	3	2081	∼1.000	Step mean = 16.1; step variance = 20.0
ln(Trunk Length)/logmm				
Stasis	2	26.35	0.297	Trait mean = 7.15; trait variance = 0.149
Brownian motion (BM)	2	26.27	0.358	Step variance = 0.011
Brownian motion+trend	3	26.65	0.344	Step mean = 0.0096; step variance = 0.010

*A. arturi* possesses four sacral vertebrae, with short, robust ribs that converge laterally (Fig. 2D–E). Sixteen proximal–middle caudal vertebrae are preserved. They have flat ventral surfaces, widely-spaced chevron facets, and the dorsal portion of the caudal rib facet is formed by the neural arch. The scapular blade expands dorsally (Fig. 2D–E). It lacks a prominent posterodorsal kink in the outline in lateral view, unlike in *E. arcuatus* (NHMUK R1317) and *T. hawkinsii* (GSM 26035). It lacks the ventral projection at the distal end of the scapular blade of ‘*P*.’ *cliduchus* ([51], fig. 14). The ilium is approximately straight in lateral view, and has an anteroposteriorly expanded distal end similar to *S. taylori* and many basal plesiosaurians. However, unlike in *S. taylori*, a deep fossa bounded anteriorly by a prominent proximodistally oriented ridge is present on the medial surface (Fig. 2D–E). The femur is approximately straight and slightly more expanded posterodistally than anterodistally (the left femur has a symmetrical distal expansion). The tibia is slightly longer than the fibula.

Plesiosauroidea Welles, 1943 [52]

*Eoplesiosaurus* n. gen.

urn:lsid:zoobank.org:act:38145ACA-ECE1-4450-BEDD-76DABB807E69

#### Type and only species

*Eoplesiosaurus antiquior*

#### Diagnosis

As for the type and only species.

**Eoplesiosaurus antiquior** n. gen. et sp.

urn:lsid:zoobank.org:act:4FE5EE6F-6BCF-453C-B224-51377E656134

#### Holotype and only specimen

**Figure 3:**
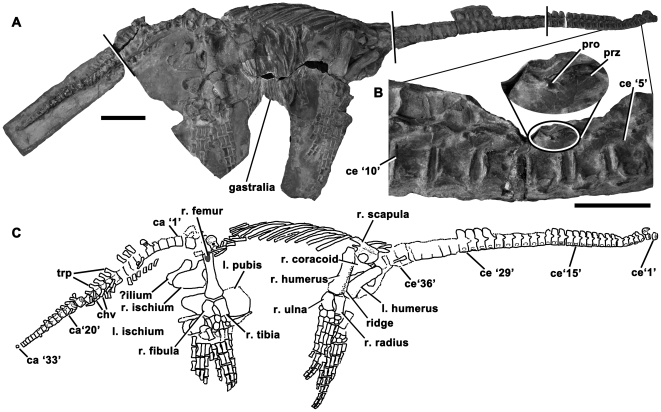
Holotype of *Eoplesiosaurus antiquior* (TTNCM 8348) in right lateral view. Image in **A** is a composite made from four photographs (divisions are indicated by black and white lines), with enlargement of anterior cervical vertebrae (**B**; magnified portion is enlarged x2.0 times). Gastralia are not shown in line drawing (**C**). Abbreviations: ca, caudal vertebra; ce, cervical vertebra [number following indicates order in preserved series]; chv, chevron; l., left [followed by name of element]; pro, lateral projection; prz, prezygapophysis; r., right [followed by name of element]; trp, transverse process. Scale bars equal 200 mm (**A**, **C**) and 50 mm (**B**).

Somerset County Museum, Taunton, UK [TTNCM] 8348, a postcranial skeleton (Fig. 3) from Watchet, Somerset UK (likely lowermost Hettangian; see above; *Plesiosaurian faunal composition at Street*).

#### Etymology

Genus name from *Eo*, Greek meaning ‘dawn’, and *Plesiosaurus*, referring to a proportionally long neck, which is also present in *Plesiosaurus*. The species epithet is Latin meaning ‘more ancient’, in reference to the old geologic age of the specimen and *Duria antiquior* (‘a more ancient Dorset’; 1830), a watercolour depiction of Lower Jurassic fauna, including plesiosaurs and ichthyosaurs, by Henry De la Beche.

#### Diagnosis

Proportionally long-necked plesiosaurian (ratio of neck:trunk length>1.2) with at least 38 cervical vertebrae and one autapomorphy: small, conical lateral projections on the bases of the anterior cervical prezygapophyses.

#### Short description of holotype

TTNCM 8348 is an almost complete postcranial skeleton, although many features are obscured by matrix (Fig. 3). Thirty-six cervical vertebrae are preserved. These do not include the atlas-axis complex, so at least 38 were present originally, more than in *A. arturi* (estimated 26–28 cervicals), ‘*R*.’ *megacaphalus* (28 cervicals; LEICT G221.1851) or *T. hawkinsii* (31 cervicals [35]). The cervical rib facets are divided by a narrow horizontal groove. A small, conical eminence projects from the base of the prezygapophyses in anterior cervical vertebrae of *E. antiquior* (Fig. 3B). This differs from the posterior projection of the prezygapophyses of *S. taylori* (Fig. 1C) and is an autapomorphy of *E. antiquior*. Unlike in *A. arturi, E. arcuatus*, ‘*P*.’ *cliduchus* (CAMSM J.35180), *S. taylori* and *T. hawkinsii*, only the anteriormost 12 preserved cervical neural spines curve posterodorsally. More posterior neural spines are inclined straight posterodorsally (Fig. 3). The dorsal vertebrae are enclosed in matrix. Thirty-three possible caudal vertebrae are preserved, although a few distal elements may be missing, and it is possible that the anterior few possible caudals are sacral vertebrae; this is difficult to determine due to incomplete preparation. The ventral surfaces of the middle caudal centra are exposed. They bear prominent chevron facets posteriorly.

The pectoral girdle is preserved, although the clavicle-interclavicle complex is incompletely prepared and difficult to observe, and the coracoids are covered by the humeri (Fig. 3). The right scapula is partly visible. It lacks the ventral projection of the distal blade that is present in ‘*P*.’ *cliduchus* ([51], fig. 14). The humerus curves posterodistally. A prominent longitudinal ridge is present on its anterior surface (Fig. 3), as observed in *E. arcuatus* and ‘*R*.’ *megacephalus* by Smith & Dyke ([41], character 92). A possible ilium is covered by the right ischium. The left pubis is approximately as long anteroposteriorly as it is wide mediolaterally. Fore- and hindflippers are well-preserved and partly articulated (Fig. 3). A small postaxial ossicle is present between the right ulna and ulnare.

Microcleididae n. fam.

urn:lsid:zoobank.org:act:5FC5F46D-6A91-4453-8F03-F55B31A74BCD

**Type genus. Microcleidus** Watson, 1909 [53]

#### Phylogenetic definition

*Microcleidus homalospondylus* and all taxa more closely related to it than to *Plesiosaurus dolichodeirus, Cryptoclidus eurymerus, Elasmosaurus platyurus, Leptocleidus superstes, Pliosaurus brachydeirus or Polycotylus latipinnis*.

#### Diagnosis

Plesiosauroids possessing: widely separated posterior cervical rib facets (character 123.1 in the *Phylogenetic analysis*; below); posteriormost dorsal rib facets split between centrum and neural arch (‘sacralised’, but bearing a dorsal rib) (146.1); medial surface of the iliac blade anteroposteriorly concave (178.0; also present in some rhomaleosaurids); and a prominent flange extends anteriorly from the proximal half of the radius (197.1; also present in *Hauffiosaurus* and some rhomaleosaurids). This diagnosis focuses on unambiguous postcranial synapomorphies because cranial material of basal microcleidids is unknown.

#### Included taxa

*Microcleidus homalospondylus, M. (Hydrorion) brachypterygius* (nov. comb), *M. (Occitanosaurus) tournemiensis* (nov. comb.), *Seeleyosaurus guilelmiimperatoris, Eretmosaurus rugosus* and *Westphaliasaurus simonsensii*.

#### Remarks

**Großmman** ([20], p. 556) referred informally to ‘microcleidid elasmosaurs’, a clade comprising the three taxa referred to *Microcleidus* herein. However, the family has not formally been erected until now. In the present study we recover very strong branch support for a clade uniting *Microcleidus* spp. and *Seeleyosaurus guilelmiimperatoris* (below: *Phylogenetic analysis*). This forms the nucleus of a new, formally-defined plesiosaurian family Microcleididae.

**Microcleidus** Watson, 1909 [53]

#### Type species

*Microcleidus homalospondylus* (Owen, 1865–1881) [17]

#### Additional included species

*Microcleidus (Hydrorion) brachypterygius* (von Huene, 1923) comb. nov. [54]; *Microcleidus (Occitanosaurus) tournemirensis* (Sciau *et al*., 1990 [55]) comb. nov.

#### Diagnosis

Microcleidid plesiosauroids with (selected unambiguous synapomorphies): jugal excluded from orbit margin by maxilla-postorbital contact (character 32.1 in the *Phylogenetic analysis*; below); jugal short, terminates around posterior orbital margin (33.1); cervical centra longer anteroposteriorly than high dorsoventrally (116.1); longitudinal ridge on lateral surface of cervical centrum (118.1); anteroposterior constriction at base of dorsal neural spines (142.1; also present in *Hauffiosaurus* and some rhomaleosaurids); anterior process of coracoid long and transversely narrow (165.1; also present in cryptoclidids and leptocleidians).

#### Remarks

Three highly similar Toarcian long-necked microcleidids are recovered in a well-supported clade herein (decay index = 6; below: *Phylogenetic analysis*). Pairwise dissimilarity (below: *Character disparity*) between all three species is very low; 7–17% of comparable characters have different scores (*M. homalospondylus-M. brachypterygius*, 0.07; *M. homalospondylus-M. tournemirensis*, 0.13; *M. brachypterygius-M. tournemirensis*, 0.17). Most of these values are not substantially higher than those between *Rhomaleosaurus* (2–11%; excluding ‘*R*.’ *megacephalus*) and *Hauffiosaurus* species (3–11%), and all are substantially lower than mean pairwise dissimilarity between all Toarcian plesiosaurians (0.37). On this basis, all three taxa are referred to a single genus, *Microcleidus* Watson, 1909 [53].

## Analysis

### Phylogenetic analysis

We constructed a new phylogenetic data matrix based on a thorough review of all previously published characters and direct observation of most Lower Jurassic, European plesiosaurians. This resulted in analysis of 207 characters (109 cranial, 98 postcranial; including 46 new characters; Appendix S1) and 32 taxa (5 outgroups+24 Lower Jurassic ingroup taxa+3 Middle Jurassic taxa; Appendix S1), 27 of which were examined directly. Two Lower Jurassic British plesiosaurians known from relatively complete remains were not included in the analysis: *Sthenarosaurus dawkinsi* from the Toarcian of Yorkshire is incompletely described [53] and was not examined; the holotype of ‘*Plesiosaurus*’ *macrocephalus* is a young juvenile and was not included, but a referred specimen, NHMUK 49202 [47], of currently uncertain taxonomic affiliation (e.g. [56]) was included. ‘*Plesiosaurus*’ *cliduchus* is currently difficult to access and was not examined closely or included in the phylogenetic analysis. *Plesiospterys wildi* O'Keefe 2004 [57] was scored separately from *Seeleyosaurus guilelmiimperatoris* (Dames, 1895) [58] to test the hypothesis that they are conspecific [20].

The matrix (Appendix S3) was analysed in PAUP* 4.0b10 for Macintosh [59] using 500 random addition replicates with TBR branch swapping, saving an unlimited number of trees at each step. Bremer support was calculated using the same search strategy, implemented using the ‘Decay Index PAUP File’ function of MacClade [60]. *Yunguisaurus* is likely the most basal pistosaurian included in our analysis [61] and was formally designated as the outgroup.

**Figure 4:**
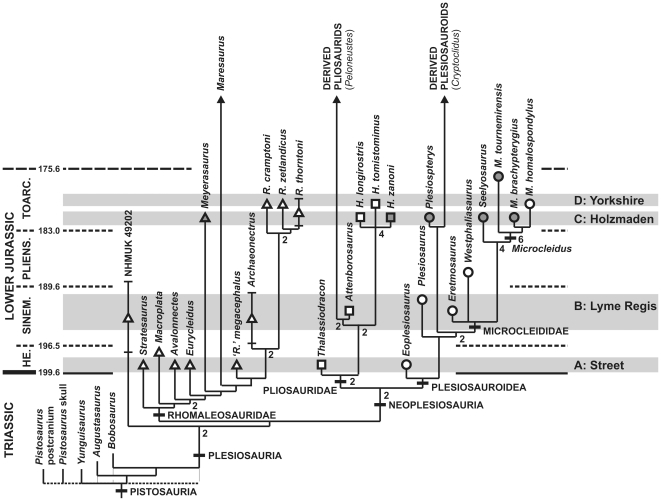
Phylogeny of Lower Jurassic plesiosaurians. Temporally-calibrated strict consensus of 42 MPTs recovered from our phylogenetic analysis. Triangular symbols represent non-neoplesiosaurian plesiosaurians (mainly rhomaleosaurids), squares represent pliosaurids and circles represent plesiosauroids. Unfilled shapes represent British taxa whereas grey-filled shapes represent German and French taxa. Key localities yielding abundant remains from four narrow horizons are indicated by grey bands, although contemporaneous specimen are known from other localities: **A**, Street, Somerset, UK (lowermost Hettangian); **B**, Lyme Regis and Charmouth, Dorset, UK (late Hettangian–Sinemurian); **C**, Holzmaden and vicinity, Baden-Württemberg, Germany (*H. falciferum* Chronozone; lower Hettangian); **D**, Yorkshire, UK (*H. bifrons* Chronozone; lower Hettangian). Dashed lines indicate polytomy at base of Pistosauria prior to deletion of *Pistosaurus* from the set of MPTs.

The analysis resulted in 42 most parsimonious cladograms, each 604 steps long with an ensemble consistency index (CI) of 0.4343, retention index (RI) of 0.5983 and rescaled consistency index (RC) of 0.2744. The strict consensus is well-resolved. Although basal pistosaurians form a polytomy, this results from the uncertain phylogenetic position of the skull of *Pistosaurus*. When *Pistosaurus* is pruned from the set of most parsimonious cladograms, *Bobosaurus* and *Augustasaurus* are resolved as successive outgroups to Plesiosauria (Fig. 4). A surprising, novel topology is recovered, in which Pliosauridae and Plesiosauroidea form a clade (Neoplesiosauria *sensu* Ketchum & Benson [56]) excluding Rhomaleosauridae. Many previous classifications, and all phylogenetic analyses have united Pliosauridae with rhomaleosaurids in a monophyletic ‘Pliosauroidea’, sometimes also including Cretaceous clades such as Polycotylidae (e.g. [40], [44], [52], [56], [62]). Neoplesiosauria receives only moderate branch support here (decay index = 2). However, it is supported by 12 synapomorphies, of which seven are unambiguously optimised: the presence of a short posteroventral process of the postorbital (character 35.2), a mediolaterally narrow parietal vault, lacking the ‘lateral angle’ (38.0), posteromedian ridge of the supraoccipital absent (58.1), parasphenoid terminates within the anterior one-third of the posterior interpterygoid vacuity forming diamond-shaped ventral platform (64.0), surangular transversely narrow and ‘blade-like’, lacking a prominent medial crest and dorsomedial fossa (99.2), distal anteroposterior width of scapular blade subequal to width at midlength (161.0; not expanded), humeral shaft has pronounced dorsodistal curvature (185.0). Strong character support for Neoplesiosauria contrasts with a previously-hypothesised ‘Pliosauroidea’, comprising Pliosauridae and Rhomaleosauridae, most proposed synapomorphies of which are actually plesiomorphies [35]. NHMUK 49202 was found basal to Rhomaleosauridae on the phylogenetic ‘stem’ leading to Neoplesiosauria as in Ketchum & Benson (2010 [56]). It possesses several plesiomorphies not found in more derived plesiosaurians, including: the premaxilla does not divide the anterior processes of the frontals (14.0); the occipital condyle lacks a constricting groove around its base, even ventrally (49.2); and the paraoccipital process is inclined dorsolaterally relative to the ventral surface of the exoccipital-opisthotic, resulting in a narrow cranioquadrate passage (54.1).

Specimens from Street and other earliest Jurassic localities include basal plesiosauroids (*Eoplesiosaurus*) and pliosaurids (*Thalassiodracon*), but most represent rhomaleosaurids (*Stratesaurus*, *Avalonnectes*, *Eurycleidus*, ‘*Rhomaleosaurus*’ *megacephalus*). Branch support for nodes within Rhomaleosauridae is low, but Pliosauridae, Microcleididae, and nodes within both families, are generally well-supported (a monophyletic *Microcleidus* comprising *M. homalospondylus*, *M. brachypterygius*, and *M. tournemirensis* received exceptionally strong support [decay index = 6]). This is part of a temporal pattern in the distribution of branch support. Older nodes are generally less well-supported, probably reflecting poor knowledge of Triassic pistosaurians: 12 nodes within Plesiosauria occur prior to the Triassic–Jurassic boundary (i.e. probably within the Triassic) and have an (mean) average decay index of 1.33; six younger nodes occur within or prior to the Sinemurian and have an average decay index of 1.67; and seven younger nodes occur prior to or within the Toarcian and have an average decay index of 2.86. Most nodes within Rhomaleosauridae split in the Triassic, whereas most pliosaurid and plesiosauroid nodes split in the Jurassic.

### Phylogenetic diversity estimates

**Figure 5:**
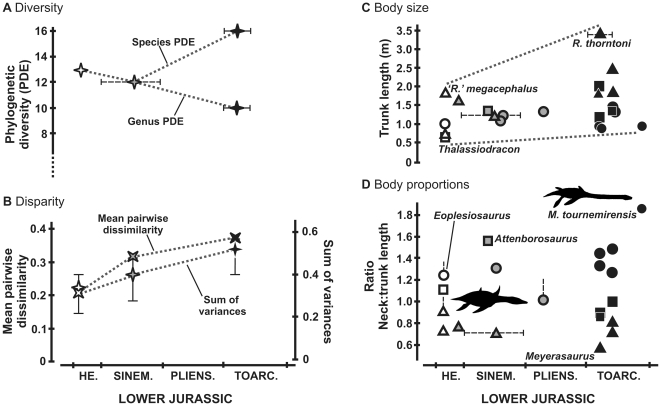
Early evolution of Plesiosauria. Plots of **A**, phylogenetic diversity [62]; **B**, disparity (main pairwise dissimilarity and sum of variances of PCO axes with 95% confidence intervals [64]); **C**, body size, based on the proxy trunk length (in metres); **D**, body proportions, based on the proxy neck:trunk length ratio. In **C–D**, triangles represent non-neoplesiosaurian plesiosaurians (mainly rhomaleosaurids), squares represent pliosaurids and circles represent plesiosauroids; unfilled shapes represent earliest Hettangian taxa, grey-filled shapes indicate late Hettangian–Pliensbachian taxa, and black shapes indicated Toarcian taxa.

‘Phylogenetic diversity estimates’ were calculated for the lower Hettangian, Sinemurian and Toarcian (Fig. 5A), counting both taxon occurrences and ‘ghost lineages’ implied by the phylogeny [63]. All Hettangian–Sinemurian operational taxonomic units (OTUs) are monotypic genera (or single specimens) whereas Toarcian OTUs include multiple species within *Hauffiosaurus*, *Microcleidus* and *Rhomaleosaurus*. Thus, genus- and species-level phylogenetic diversity estimates are identical until the Toarcian. ‘*P*.’ *cliduchus* (lower Hettangian) and *Sthenarosaurus dawkinsi* (Toarcian) were not included in our phylogeny but were added to the phylogenetic diversity estimate. This resulted in phylogenetic diversity estimates of 13 in the lower Hettangian; 12 in the Sinemurian and 10 (genera) or 16 (species) in the lower Toarcian. We prefer the genus-level estimate because German and British representatives of *Hauffiosaurus* (other than *H. longirostris*) and *Microcleidus* are not contemporaneous and may represent chronospecies that did not exist contemporaneously [48]. The lower Toarcian diversity estimate may be underestimated due to the absence of well-sampled horizons in the immediately following stages (few early Middle Jurassic plesiosaurians are known). However, unlike older taxa, lower Toarcian plesiosaurians are known from multiple, intensely sampled horizons in both Germany and the UK, likely compensating for this effect. These results confirm that plesiosaurian diversity immediately following the Triassic–Jurassic boundary was comparable to that later in the Lower Jurassic, including up to 20 million years later in the early Toarcian.

### Character disparity

Faunal disparity was estimated using our cladistic data matrix, following the protocol described by Wills *et al*. (1994 [64]) and employed by several recent studies of tetrapod disparity (e.g. [65]–[67]). Pairwise dissimilarity between all Lower Jurassic taxa was calculated as the proportion of comparable cells (those cells not scored as ‘?’ for either taxon) that differed between the taxa. Taxa known only from postcranial material such as *Eoplesiosaurus*, *Eretmosaurus* and *Westphaliasaurus* could not be compared with *Hauffiosaurus longirostris*, which has 100% postcranial missing data (a postcranial skeleton probably belonging to the holotype [68] was not scored). Thus, *H. longirostris* was deleted from the matrix before further analyses to eliminate problems of missing entries in the resulting symmetric dissimilarity matrix. This matrix formed the basis of a principal co-ordinates analysis (PCo) in Ginkgo (Universitat de Barcelona, http://biodiver.bio.ub.es/ginkgo/) using a negative eigenvalue correction (Cailliez method). Two disparity metrics were calculated for three narrow Lower Jurassic stratigraphic intervals (earliest Hettangian; Sinemurian; lower Toarcian): the mean pairwise dissimilarity (as used by e.g. Wagner 1997 [69]), and the sum of variance of scores on all 24 principal coordinate axes. The sum of variances was chosen instead of product- or range-based metrics, because it is robust to variation in sample size [66], [70]. It was computed using the freeware program RARE (M. Wills, pers. comm.), which also allows 95% confidence intervals to be computed using rarefaction. Both character-based disparity measures show monotonic increase through the Lower Jurassic (Fig. 5B).

**Figure 6:**
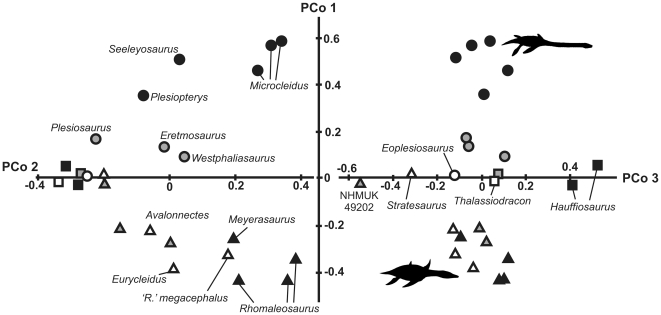
Early plesiosaurian morphospace. First three principal coordinate axes of dissimilarity among Lower Jurassic plesiosaurians. **A**, PCo2 versus PCo1, **B**, PCo2 versus PCo3.

The scree plot exhibits a break in slope between the third and fourth PCo axes, following which, each axis describes 5.73% or less of the Gower-transformed dissimilarity. The first three principal coordinate axes encompass 38.1% of the Gower-transformed dissimilarity and are plotted in Figure 6. Earliest Jurassic taxa (unfilled shapes) have mostly negative values of PCo1, and plot in the centre of PCos 2 and 3. Later rhomaleosaurids (triangles) occupy a similar region of character-distance space, likely due to their plesiomorphic anatomy. They have negative values of PCo1, but have higher positive values of PCo2. They are well-separated from later pliosaurids (squares), which plot in the centre of PCo1, have high negative values of PCo2, and high positive values of PCo3. Later plesiosauroids (circles) have high positive values of PCo1. Thus, PCo1 differentiates Toarcian (black symbols) rhomaleosaurids (negative values) from pliosaurids (values close to zero) and plesiosauroids (positive values).

### Body size evolution

Trunk length was used as a proxy for overall body size (Fig. 5C), defined as the distance between the anterior surface of the anteriormost vertebra with an elongate, ‘dorsalised’ rib and the posterior surface of the posteriormost sacral vertebra. The appearance of ribs with a ‘dorsal’ morphology (long, curving, rod-like ribs) was used because these ribs define the trunk directly, forming its lateral wall. Other criteria for identifying the neck-trunk boundary rely only on indirect proxies such as rib facet morphology. Only measurements from the largest individuals of each taxon were counted, and taxa only known from juveniles were not included in our analysis (*Plesiopterys*, *Rhomaleosaurus zetlandicus*; indicated by rounded, incompletely ossified margins of slowly-ossifying bones such as the limb girdles [*Plesiopterys*], ulna and tarsals (*Plesiopterys* and *R. zetlandicus*; compare with figures in [71]), and dorsal neural spines in *R. zetlandicus*; SMNS 16812; The Yorkshire Museum, York, UK [YORYM] G503). Minimum body size in the Triassic–Jurassic boundary fauna, as indicated by the proxy trunk length (680 mm, *Thalassiodracon*) is comparable to that among Triassic pistosaurians (680 mm, *Yunguisaurus* [61]) and low compared to later intervals (*Plesiosaurus* [Sinemurian], 1100 mm; *Seeleyosaurus* [Toarcian], 900 mm). Maximum body size also increases through time. The largest individual of ‘*Rhomaleosaurus*’ *megacephalus* from the earliest Hettangian fauna has a trunk 1820 mm long compared to 1500 mm for the Triassic pistosaurian *Bobosaurus* [72] and 3400 mm for the Toarcian *Rhomaleosaurus thorntoni* (or 2460 mm for *R. cramptoni*). Sinemurian representatives of the large-bodied rhomleosaurid lineage are not currently known. Increase in the maximum and minimum values through time is suggestive of a driven trend of size increase [73].

We tested the hypothesis of a driven trend by fitting maximum likelihood models of trunk length evolution representing stasis, Brownian motion (BM), and BM+trend, to our phylogenetic tree with temporal branch lengths estimated using stratigraphic age. This analysis was conducted in R version 2.10.1 [74], using both untransformed, and ln-transformed data. Taxa for which trunk was not known, and taxa occurring after the Lower Jurassic, were not analysed. Branch lengths were calibrated following the protocol of Brusatte *et al*. [65] using code available from http://www.graemetlloyd.com/pubdata/functions_2.r (accessed 23 December 2011). Evolutionary models were fitted using the ‘fitContinuous’ function of the Geiger package [75] as described by Hunt & Carrano [76], specifying a trait standard deviation of 55 mm, based on four individuals of *Thalassiodracon hawkinsii*. The results indicate overwhelming support for the BM+trend model when untransformed trunk length is analysed (Table 2). However, ln-transformed trunk length fits all tested models subequally well (Table 2). Thus, the appearance of driven trend in the untransformed data may arise from overweighting of extreme large sizes in taxa like *Rhomaleosaurus thorntoni*, and the true signal cannot be distinguished from white noise or Brownian motion.

### Body proportions

Neck length excluding the skull was measured for the same individuals measured for trunk length (above). The ratio of neck length:trunk length was used as a proxy for overall body proportions (Fig. 5D). Other studies have used larger sets of measurements to quantify body plans [2]–[3]. The approach employed here was selected to maximize taxonomic coverage as it can be applied to some relatively incomplete specimens (e.g. those missing limbs and skulls).

Body proportions, as indicated by the proxy neck length/trunk length, occupy a narrow range in the earliest Hettangian fauna (0.72 in ‘*R*.’ *megacephalus* – 1.25 in *Eoplesiosaurus* [although this represents a slight underestimate because a small number of anterior cervical vertebrae are not preserved]). Sinemurian (0.71 in *Archaeonectrus* – 1.57 in *Attenborosaurus*) and Toarcian (0.57 in *Meyerasaurus* – 1.90 in *Microcleidus tournemirensis*) show small decreases in the minimum ratio and larger increases in the maximum ratio, demonstrating an increase in the range of plesiosaurian body plans though the Lower Jurassic.

## Discussion

### Early evolution of Plesiosauria – evolutionary responses to pelagic adaptation

During the Mesozoic, at least 12 tetrapod lineages independently became adapted to marine life (e.g. [5]). Of these, most remained restricted to nearshore, shallow water environments. Many of these possessed ‘plesiopedal’ (i.e. terrestrially proportioned) limbs (e.g. placodont sauropterygians [4]; thalattosaurs [77]–[78]; basal mosasauroids and related squamate groups [79]). Others possessed long, slender bodies (e.g. basal ichthyosaurs [7], [10]; some basal sauropterygians [1], [4], [80] indicating low cruising efficiency [81] and locomotion by axial undulation, resulting in low stamina [82]. Thus these plesiomorphic body forms conferred only limited ability to survive in the open ocean, where food resources may be distributed patchily and locomotor efficiency is required. Derived ichthyosaurs [10], plesiosaurs [1], marine turtles, and possibly the mosasauroid *Plotosaurus* [83] became adapted for life in the open ocean by shortening and stiffening the trunk, and relying on caudal oscillation (ichthyosaurs and *Plotosaurus*) or limb-driven locomotion (plesiosaurs and turtles; the stiff trunk is primitive for turtles). Most of these clades also show evidence of viviparity [10], [84]–[86], freeing them altogether from the constraint of terrestrial locomotion in an otherwise aquatically-adapted animal, and inherent restriction of body size and proportions. *Archelon*, the largest marine turtle, provides evidence that oviparity, and the necessity of terrestrial locomotion constrains maximum body size in pelagic tetrapods. *Archelon* attained a maximum length of approximately four metres ([87], citing [88]: “3–5 m”). This is small compared to the largest ichthyosaurs (e.g. *Shonisaurus*; 20 metres long [89]), plesiosaurians (‘*Stretosaurus*’ ( = ?*Pliosaurus*) *macromerus*) and *Kronosaurus*; >10 metres [90]–[91]), and cetaceans (e.g. *Balaenoptera musculus*; commonly 25–27 metres, but up to 33.58 metres [92]).

Earliest Jurassic plesiosaurians possess their full suite of adaptations for specialised marine locomotion: reduction of intralimb flexibility, hyperphalangy, interlocking phalanges, and the appearance of supernumary ossiciations in the limbs resulting in the formation of a flipper; increased thoracic rigidity by modification of the limb girdles to form large ventral plates, and enlargement of the gastral basket; and shortening of the tail [1]. More basal, Triassic sauropterygians, including basal pistosaurians, possessed only a subset of these adaptations, demonstrated different swimming dynamics, and were restricted to shallower water facies [1], [61], [93].

Our data allow an assessment of the evolutionary response of plesiosaurs to full pelagic adaptation in the Lower Jurassic. They show that the advent of plesiosaurian locomotion was followed by a gradual increase in body size spanning approximately 17 million years between the Triassic–Jurassic boundary and the lower Toarcian. Thus, the largest lower Toarcian plesiosaurian, *Rhomaleosaurus thorntoni*, has a trunk almost twice as long as ‘*Rhomaleosaurus*’ *megacephalus* from the lower Jurassic fauna (Fig. 5C). If linear dimensions scale isometrically in rhomaleosaurids, this implies an eight-fold increase in body mass (this is inexact, but provides an estimate of the order of magnitude of the change). This trend of increasing maximum body size continued in later plesiosaurian evolution; the largest pliosaurids, known from fragmentary Late Jurassic remains, were substantially larger than *R. thorntoni*. For example, ‘*Stretosaurus*’ ( = *Pliosaurus*) *macromerus* has a humeral length of 840 mm and femoral length of 960 mm [90], compared to 720 mm and 680 mm in *R. thorntoni* (NHMUK R4853). Minimum body size also increased through the Lower Jurassic, though less prominently (Fig. 5C). *Seeleyosaurus*, the smallest Toarcian plesiosaurian, has a trunk length 1.32 times that of *Thalassiodracon*, the smallest plesiosaurian from the lowermost Jurassic. It is noteworthy that small-bodied individuals and taxa (*Thalassiodracon*, *Avalonnectes*, *Stratesaurus*) are abundant in the earliest Jurassic fauna, but rarer in the Toarcian (Fig. 5C). However, analyses of ln-transformed trunk length data suggest that the pattern of body size increase in early plesiosaurian evolution cannot be distinguished from a Brownian motion, or ‘passive expansion’ model (Table 2). This is similar to the pattern observed in Mesozoic birds [94], and during early dinosaur evolution [95], suggesting that driven trends of body size change (‘Cope's Rule’) do not always occur during Mesozoic ecological radiations.

The range of plesiosaurian body plans also increased through the Lower Jurassic (Fig. 5D). Although more basal pistosaurians exhibit high variation in cervical vertebral counts (e.g. 20 in *Bobosaurus* [72], 44 anterior to the pectoral girdle in *Yunguisaurus* [61]), this is not reflected in their overall body proportions, as even *Yunguisaurus* has an ‘intermediate’ neck length that is subequal to that of the trunk [61]. The earliest plesiosaurians include taxa with intermediate and short neck lengths, as well as *Eoplesiosaurus*, with a neck at least 1.2 times the trunk in length (Figs 3, 5D). The range of body proportions increased progressively through the Lower Jurassic, and Toarcian plesiosaurians include long-necked taxa such as *Microcleidus tournemirensis*, in which the neck is 1.9 times the trunk in length [96]. These body proportions are similar to those of some Late Jurassic plesiosaurians, such as the cryptoclidid *Muraenosaurus* [40], [97], but Cretaceous elasmosaurids exhibited substantially longer necks (e.g. ratio = 2.75 in ‘*Alzadasaurus pembertoni*’ ( = *Styxosaurus snowii*) [98]).

### Diversity dynamics in Lower Jurassic plesiosaurian evolution

The first appearance of plesiosaurians in the earliest Jurassic is characterised by a high diversity of rhomaleosaurids (five taxa). Despite the absence of derived plesiosaurians in older deposits, rhomaleosaurids from the earliest Jurassic are widely spread across morphospace (Fig. 6), and represent nodes deeply nested within Rhomaleosauridae. Thus, most rhomaleosaurid divergence likely occurred in the Late Triassic. In contrast, although the basal neoplesiosaurian split between Pliosauridae and Plesiosauroidea probably occurred in the Triassic, most nodes within these clades occur in the Jurassic, and both clades make their earliest Jurassic first appearance at low diversity (one taxon each). Plesiosauroidea became increasingly diverse and disparate through the Early Jurassic, especially at small–medium body sizes (Fig. 5C). This diversification may have been at the expense of rhomaleosaurids and represents a clear example of a candidate clade replacement event [99]; although the earliest rhomaleosaurids occupy the entire range of contemporaneous body sizes and are widely spread across morphospace, later (Toarcian) rhomaleosaurids are exclusively large-bodied (trunk length>1.5 metres) and are restricted to a local area of morphospace (Fig. 6). Although all rhomlaeosaurids have proportionally short necks (Fig. 5D), the youngest rhomaleosaurids have the shortest necks, and thus the most extreme ‘pliosauromorph’ body plans. This may be associated with a restriction of rhomaleosaurids to a macropredaceous niche prior to their final demise after the Middle Jurassic (last occurrences: *Maresaurus*, Bajocian [100]; *Borealonectes*, Callovian [101]).

### Disparity dynamics in Lower Jurassic plesiosaurian evolution

Despite their high diversity (Fig. 5A), the earliest Jurassic plesiosaurians are characterised by low disparity (Fig. 5B). In fact, mean pairwise dissimilarity (the number of character differences among comparable cells) is approximately half that 17 million years later in the lower Toarcian. Plesiosaurian disparity based on character data and the range of body proportions represented (see above) both increase monotonically through the Lower Jurassic. This mode of disparity accumulation, coupled with more or less constant species diversity, differs from that shown in the origins of major groups such as the Cambrian ‘explosion’ of metazoan disparity (e.g. [102]) and the rapid evolution of early tetrapod disparity in the early Carboniferous [103]. Although further analyses of plesiosaurian disparity over a longer time interval are required to confirm this pattern, the results obtained here suggest that invasion of morphospace by plesiosaurs following invasion of pelagic habitats was gradual, not explosive, and comparable to that in other tetrapod subclades such as dinosaurian and crurotarsan archosauromorphs [65].

### Late Triassic marine tetrapod extinctions

The observation of high taxic diversity in earliest Jurassic plesiosaurians, especially among rhomaleosaurids, suggests that an extinction event precisely at, or shortly before (∼100–150 kyrs [22]) the Triassic–Jurassic boundary had little effect on Jurassic plesiosaurians. It is impossible to date these rhomaleosaurid divergences accurately, but generally low pairwise dissimilarity exhibited by earliest Jurassic plesiosaurians suggests relatively recent divergence, and may represent the influence of earlier Late Triassic extinctions, which are concentrated at the end of the Norian [21]. Our understanding of the effect of Late Triassic extinctions on plesiosaurian evolution will remain incomplete until the discovery of more complete Late Triassic plesiosaurian fossils.

Thorne *et al*. [67] attributed a monumental decimation of ichthyosaur disparity between Late Triassic and Lower Jurassic time bins to a Triassic–Jurassic boundary extinction event. However, the ‘Late Triassic’ time bin of their analysis includes six taxa from the Carnian, 23.4 million years before the end of the Triassic (235–228 Ma [104]). Only four taxa represent the younger Norian stage (28 Ma; 228–204 Ma), and zero represent the latest Triassic Rhaetian (2.4 Ma; 204–201.6 Ma). Clearly, this coarse level of temporal and taxonomic resolution is too poor to attribute a Late Triassic decline in ichthyosaur disparity to a sudden extinction event at the Triassic–Jurassic boundary. Indeed, a progressive, not precipitous, decline is suggested by the staggered last appearances of other marine reptile clades including thalattosaurs (Carnian, early Late Triassic) and basal sauropterygian clades such as nothosauroids (Ladinian, Middle Triassic) and pachypleurosaurs (Carnian) [105]. This decline occurred concurrently with lowering sea levels and a reduction in shallow marine area [4], [6], [106]. A gradualistic, or stepwise pattern of extinction is also exhibited by well-sampled marine invertebrate clades, including bivalves, conodonts, and ammonoids, which show intervals of concentrated extinction prior to the end of the Triassic, most notably at the end of the Norian (e.g. [21], [107]–[111]) and throughout the Rhaetian [112].

## Supporting Information

Appendix S1 **Measurements, age, provenance data and lists of specimens examined for taxa included in the phylogenetic analysis.**
[XLS]

Appendix S2 **Character list for the phylogenetic analysis.**
[DOC]

Appendix S3 **Phylogenetic data matrix.**
[PDF]
