# Supplementary material for: High Diversity, Low Disparity and Small Body Size in Plesiosaurs (Reptilia, Sauropterygia) from the Triassic–Jurassic Boundary
Source: PLoS One. 2012 Mar 16;7(3):e31838. doi: 10.1371/journal.pone.0031838 (PMC3306369; doi:10.1371/journal.pone.0031838)
Supplement: Appendix S2 — Character list for the phylogenetic analysis. [DOC] [file pone.0031838.s002.doc]

**Benson RBJ, Evans M, Druckenmiller PS. High diversity, low disparity and small body size in plesiosaurs from the Triassic–Jurassic boundary**

**Appendix S2—character list**

Character list for phylogenetic analysis including full citations of previous character uses and optimisations on the strict consensus cladogram (Fig. 4). In optimistaions, the notation +++ refers to the clade including a particular taxon and all more derived taxa. For example, ‘*Meyerasaurus* +++’ indicates the clade comprising *Meyerasaurus* and all more derived rhomaleosaurids.

The character list includes parsimony-uninformative characters that code derived features of Pliosauridae (only present in *Peloneustes* in the present analysis), Cryptoclididae and other derived plesiosaurians (only present in *Cryptoclidus* in the present analysis). These are intended to make the dataset useful for determining the affinities of future discoveries from the Middle and Late Jurassic. The list also includes character states not present in any taxa in the present analysis. These are included because this list will form the basis of future studies of global plesiosaurian relationships.

**Character list**

**1. Transverse constriction of rostrum at premaxilla-maxilla suture:** absent (0); present (1).

Unambiguous: *Macroplata* +++ (0->1), NHMUK 49202 (0->1), *Peloneustes* (0->1).

Sato (2002, character 12), Großmann (2007, character 2), Druckenmiller & Russell (2008a, character 10), Smith & Dyke (2008, character 10), Ketchum & Benson (2010, character 4).

Modified from O’Keefe (2001, character 9).

**2. Maxilla, lateral expansion of maxilla posterior to maxilla-premaxilla suture accomodates expanded canniniform roots** [resulting in 'second constriction' of O'Keefe 2001]**:** absent (0); present (1).

Unambiguous: *Rhomaleosaurus* *zetlandicus* + *R. thorntoni* (0->1), Rhomaleosauridae + Neoplesiosauria (1->0), *Macroplata* (0->1), *Maresaurus* (0->1), *Yunguisaurus* (1->0).

Modified from O’Keefe (2001, character 9).

**3. Ratio of orbit length in dorsal view to temporal fenestra length:** 0.3–0.6 (0); >0.8 (1).

Unambiguous: *Maresaurus* +++ (1->0), *Hauffiosaurus* +*Attenborosaurus* + *Peloneustes* (1->0), *Microcleidus* [node] (1->0), Plesiosauria (0->1), *Macroplata* (1->0).

Modified from Bardet *et al*. (1999, character 5), Sato (2002, character 35), Gasparini *et al*. (2003, character 4), Großmann (2007, character 4), Druckenmiller & Russell (2008a, character 4), Smith & Dyke (2008, character 33), Ketchum & Benson (2010, character 1), Vincent (2010, character 3) (different quantification).

**4. Ratio of pre-orbital skull length to total skull length:** 0.3–0.5 (0); >0.6 (1).

Unambiguous: *Hauffiosaurus* +*Attenborosaurus* + *Peloneustes* (0->1), *Archaeonectrus* (0->1), *Augustasaurus* (0->1), *Macroplata* (0->1), *Pistosaurus* [skull] (0->1).

Modified from O’Keefe (2001, character 6), O’Keefe (2001, character 8), Gasparini *et al*. (2002, character 1), Sato (2002, characters 2, 13, 39), Albright et al. (2007, character 7), Druckenmiller & Russell (2008a, character 2), O’Keefe & Street (2009, character 5), O’Keefe & Wahl (2003, character 7), Smith & Dyke (2008, character 3), Ketchum & Benson (2010, character 2), Vincent (2010, character 1).

**5. Dorsal margin of orbit, outline in dorsolateral view:** concave, forming part of suboval orbit (0); convex, skull roof projects into orbit (1).

Unambiguous: *Peloneustes* (0->1), *‘R.’ megacephalus* (0->1).

Smith & Dyke (2008, character 1).

The appearance of this feature in NHMUK 49202 is due to crushing. However, it is present in *Peloneustes* (Andrews 1913; Linder 1913; Ketchum & Benson 2011a) and ‘*Rhomaleosaurus*’ *megacephalus* (Cruickshank 1994; LEICT G221.1851).

**6. Relative skull length compared to length of dorsal series:** 0.20–0.39 (0); >0.40 (1).

Unambiguous: *Peloneustes* (0->1).

DELTRAN: *‘R.’ megacephalus* +++ (0->1).

ACCTRAN: *Maresaurus* +++ (0->1).

Modified from O’Keefe (2001, character 1), Sato (2002, character 4), O’Keefe & Wahl (2003, character 1), Druckenmiller & Russell (2008a, character 1).

**7. Inclination of the suspensorium:** sub-vertical or weakly inclined (~80–90 degrees) (0); significantly inclined (<70 degrees) (1).

Modified from Carpenter (1999, character 9), Sato (2002, character 75), Druckenmiller & Russell (2008a, character 36), Ketchum & Benson (2010, character 45).

Unambiguous: *Thalassiodracon* (0->1).

DELTRAN: *Maresaurus* +++ (0->1).

ACCTRAN: *Macroplata* +++ (0->1).

**8. Relative positions of external and internal nares:** internal naris posterior to external naris (0); nares overlap (1); internal naris anterior to external naris (2).

Unambiguous: *Hauffiosaurus* +*Attenborosaurus* + *Peloneustes* (1->2), Plesiosauria (0->1).

DELTRAN: *Meyerasaurus* +++ (1->2).

ACCTRAN: *Macroplata* +++ (1->2).

Storrs (1991).

Druckenmiller & Russell (2008a, character 13), Ketchum & Benson (2010, character 6).

Modified from Sato (2002, character 19).

**9. Position of the mandibular glenoid fossa:** just posterior to the occipital condyle (0); coplanar with the occipital condyle (1); far posterior to occipital condyle, distance at least equal to basicranial (basioccipital+basisphenoid) length (2).

Unambiguous: *Maresaurus* +++ (0->2), NHMUK 49202 (0->2), *M. tournemirensis* (0->2), *Stratesaurus* (0->1).

DELTRAN: *Cryptoclidus* (0->1).

ACCTRAN: *Plesiopterys* + *Cryptoclidus* (0->1).

Modified from Sato (2002, character 90), Druckenmiller & Russell (2008a, character 81), Ketchum & Benson (2010, character 93) (new state 2 added).

**10. Fluted ornamentation of bone surface around the orbit margin:** absent (0); present (1).

DELTRAN: *Peloneustes* (0->1).

ACCTRAN: *Attenborosaurus* + *Peloneustes* (0->1).

Ketchum & Benson (2010, character 34).

**11. Temporal bar, suborbital margin:** smoothly curved (0); squared-off posteroventral margin of suborbital skull forms abrupt edge (1).

Unambiguous: *Peloneustes* (1->0).

DELTRAN: Rhomaleosauridae + Neoplesiosauria (0->1), *‘R.’ megacephalus* (1->0).

ACCTRAN: *Eurycleidus* +++ (1->0), Plesiosauria (0->1).

New character.

**12. Alveolar margin of upper jaw in lateral view:** approximately straight or weakly convex (0); undulating, forming 'scalloped' margin (1).

DELTRAN: *Maresaurus* +++ (0->1), *Hauffiosaurus* [node] (0->1), *Attenborosaurus* (0->1), *Macroplata* (0->1).

ACCTRAN: *Macroplata* +++ (0->1), *Hauffiosaurus* +*Attenborosaurus* + *Peloneustes* (0->1), *Meyerasaurus* (1->0), *Peloneustes* (1->0).

New character.

**13. Premaxilla, external surfaces:** marked neurovascular foramina but otherwise smooth (0); numerous sharp rugose crests (1); consistently undulating with rounded ridges (2).

Unambiguous: *Archaeonectrus* +++ (2->0).

DELTRAN: *Meyerasaurus* +++ (0->2), *Seeleyosaurus* +++ (0->2), *H. longirostris* (0->2).

ACCTRAN: *Avalonnectes* +++ (0->2), *Hauffiosaurus* [node] (0->2), Microcleididae (0->2).

State 1 from Benson (in press, character 203).

State 2 from from Großmann (2007, character 1), Ketchum & Benson (2010, character 5).

**14. Premaxilla contact along the dorsal midline:** contacts anterior extension of frontals only (0); partially splits frontal along the midline (1); completely splits the frontals and contacts the parietals (2).

Unambiguous: *Archaeonectrus* +++ (1->2), Rhomaleosauridae + Neoplesiosauria (0->1).

DELTRAN: *Peloneustes* (1->2).

ACCTRAN: *Attenborosaurus* + *Peloneustes* (1->2).

Ketchum & Benson (2010, character 10), Vincent (2010, character 5).

Modified from O’Keefe (2001, character 11), Sato (2002, character 6), O’Keefe & Wahl (2003, character 8), Druckenmiller & Russell (2008a, character 5).

State 1 from from Bardet et al (1999, character 8, Smith & Dyke (2008, character 17; modified from Großmann (2007, character 6).

State 2 from Carpenter (1999, character 8).

**15. Premaxilla, posterior termination:** tapering and non-interdigitating or weakly interdigitating (0); broad, deeply interdigitating suture with the frontal or parietal (1); transversely broad butt joint (2).

Unambiguous: Pliosauridae (0->1), NHMUK 49202 (0->2).

Modified from Sato (2002, character 6), Benson (in press, character 14) (addition of state 2, an autapomorphy of NHMUK 49202)

**16. Premaxilla, dorsomedian ridge:** absent or indistinct (0); prominent (1).

DELTRAN: *Macroplata* +++ (0->1), *Attenborosaurus* (0->1), *H. longirostris* (0->1), NHMUK 49202 (0->1), *Thalassiodracon* (0->1).

ACCTRAN: Plesiosauroidea (1->0), Plesiosauria (0->1), *H. tomistomimus* (1->0), *Peloneustes* (1->0), *Stratesaurus* (1->0).

Sato (2002, character 3), Druckenmiller & Russell (2008a, character 6), Smith & Dyke (2008, character 5), Ketchum & Benson (2010, character 11).

**17. Premaxilla, shape of dorsomedian ridge:** narrow and crest-like (taller than wide) (0); broad (1).

Unambiguous: Rhomaleosauridae + Neoplesiosauria (0->1).

DELTRAN: *Rhomaleosaurus* [node] (1->0), *‘R.’ megacephalus* (1->0).

ACCTRAN: *‘R.’ megacephalus* +++ (1->0), *Archaeonectrus* (0->1).

Ketchum & Benson (2010, character 12).

**18. Premaxilla, dorsomedian ridge location:** anterior (0); posterior (1); elongate, extends from orbits to rostral tip (2).

Unambiguous: *Archaeonectrus* (0->1), *R. thorntoni* (0->2).

DELTRAN: Pliosauridae (0->2), *Macroplata* (0->1), *Maresaurus* (0->2).

ACCTRAN: *‘R.’ megacephalus* +++ (2->0), Rhomaleosauridae + Neoplesiosauria (0->2), *Macroplata* (2->1).

Ketchum & Benson (2010, character 13).

**19. Premaxilla, dorsomedian foramen:** absent (0); present (1).

Unambiguous: *Archaeonectrus* +++ (0->1).

Cruickshank (1997).

O’Keefe (2001, character 13), Sato (2002, character 9), Druckenmiller & Russell (2008a, character 9), Smith & Dyke (2008, character 7), Ketchum & Benson (2010, character 9).

**20. Premaxilla–internal naris contact**: premaxilla participates broadly in the margin of the external naris (0); does not participate (1); small contact at anterodorsal corner of external naris (2).

Unambiguous: *Stratesaurus* (0->1).

DELTRAN: *‘R.’ megacephalus* +++ (0->2), *Hauffiosaurus* +*Attenborosaurus* + *Peloneustes* (0->1).

ACCTRAN: *Maresaurus* +++ (0->2), Pliosauridae (0->1).

Smith & Dyke (2008, character 8 (order of states different).

Modified from O’Keefe (2001, character 12), Sato (2002, character 8), O’Keefe & Wahl (2003, character 9), Druckenmiller & Russell (2008a, character 7), Ketchum & Benson (2010, character 8) (addition of state 2).

**21. Premaxilla, constriction of posteromedian process at level of extenal naris:** absent (0); present (1).

DELTRAN: *Hauffiosaurus* +*Attenborosaurus* + *Peloneustes* (1->0).

ACCTRAN: Pliosauridae (1->0).

New character.

The derived condition occurs even in some taxa in which the premaxilla does not contact the external naris such as *Stratenectes* (Fig. 1A–B).

**22. Premaxilla-maxilla sutures:** converging gradually posteromedially for entire length (0); anterior portion extends dorsomedially then abruptly curves posteriorly, resulting in a parallel-sided appearance of the posterior process of the premaxilla (1).

Unambiguous: *Rhomaleosaurus* [node] (0->1).

Smith & Dyke (2008, character 9), Benson (in press, character 204).

**23. Maxilla, posterior extent of maxillary tooth row:** ventral to postorbital bar (0); ventral to temporal fenestra (1); around orbital midlength or more anteriorly (2).

Unambiguous: *M. homalospondylus* (0->1).

DELTRAN: *Hauffiosaurus* [node] (0->2), *Peloneustes* (0->2).

ACCTRAN: *Hauffiosaurus* +*Attenborosaurus* + *Peloneustes* (0->2), *Attenborosaurus* (2->0).

Modified from characters describing the posterior extent of the maxilla:

State 1 from Großmann (2007, character 12).

State 2 from Sato (2002, character 14).

**24. Maxilla-internal naris contact:** maxilla participates in the margin of the internal naris (0); does not participate (1).

DELTRAN: *Cryptoclidus* (0->1).

ACCTRAN: *Plesiopterys* + *Cryptoclidus* (0->1).

Bardet et al (1999, character 11), Gasparini et al. (2002, character 5), Sato (2002, character 42), Gasparini et al. (2003, character 8), Druckenmiller & Russell (2008a, character 40), Ketchum & Benson (2010, character 50), Vincent (2010, character 20).

**25. Maxilla, trough or depressed region anterior to external naris:** absent (0); present, defined laterally by a longitudinal ridge but does not extend far anteriorly (1); prominent longitudinal trough extends most of the prenarial length of the maxilla (2).

Unambiguous: *Hauffiosaurus* [node] (0->2), *Macroplata* (0->1), NHMUK 49202 (0->1), *‘R.’ megacephalus* (0->1).

State 1 from O’Keefe (2001, character 37, Druckenmiller & Russell (2008a, character 12, Smith & Dyke (2008, character 6, Ketchum & Benson (2010, character 7).

State 2 from Benson et al. (2011b, character 179).

**26. Frontal-external naris contact:** frontal does not participate in the margin of the external naris (0); participates (1).

DELTRAN: Rhomaleosauridae (0->1), *Hauffiosaurus* +*Attenborosaurus* + *Peloneustes* (0->1).

ACCTRAN: Plesiosauroidea (1->0), Rhomaleosauridae + Neoplesiosauria (0->1).

Bardet et al (1999, character 9), O’Keefe (2001, character 19), Sato (2002, character 25), Gasparini *et al*. (2003, character 6), O’Keefe & Wahl (2003, character 13), Druckenmiller & Russell (2008a, character 8), Smith & Dyke (2008, character 15), Ketchum & Benson (2010, character 18).

Modified from Vincent (2010, character 6).

**27. Frontal enters margin of temporal fenestra:** does not (0), does narrowly because supratemporal fossa extends far anteriorly (1).

Unambiguous: Rhomaleosauridae + Neoplesiosauria (1->0), *Macroplata* (0->1), *‘R.’ megacephalus* (0->1).

O’Keefe (2001, character 18), Sato (2002, character 22), O’Keefe & Wahl (2003, character 12), Druckenmiller & Russell (2008a, character 24), Ketchum & Benson (2010, character 22), Vincent (2010, character 7).

**28. Frontal, posterolateral process:** present (0); absent (1) (inapplicable for taxa in which the premaxilla contacts the parietal).

Unambiguous: *Yunguisaurus* (0->1).

DELTRAN: *Plesiopterys* + *Cryptoclidus* (0->1), *Plesiosaurus* (0->1).

ACCTRAN: Microcleididae (1->0), Plesiosauroidea (0->1).

O’Keefe (2001, character 16), O’Keefe & Wahl (2003, character 11), Druckenmiller & Russell (2008a, character 25), Ketchum & Benson (2010, character 21), Vincent (2010, character 8).

**29. Frontal-orbit contact:** frontal participates in the orbit margin (0); does not participate [excluded by prefrontal-postfrontal contact] (1).

Unambiguous: *Microcleidus* *homalospondylus* + *M. brachypterygius* (0->1), *Pistosaurus* [skull] (0->1), *‘R.’ megacephalus* (1->0).

DELTRAN: *Macroplata* +++ (0->1), NHMUK 49202 (0->1).

ACCTRAN: Neoplesiosauria (1->0), Plesiosauria (0->1).

O’Keefe (2001, character 40), Sato (2002, character 20), Druckenmiller & Russell (2008a, character 26), Smith & Dyke (2008, character 18), Ketchum & Benson (2010, character 19).

**30. Lacrimal:** absent, maxilla participates in orbit margin (0); present, maxilla excluded from orbit margin (1).

DELTRAN: *H. tomistomimus* (0->1), *Peloneustes* (0->1).

ACCTRAN: *Hauffiosaurus* +*Attenborosaurus* + *Peloneustes* (0->1), *Attenborosaurus* (1->0).

Druckenmiller & Russell (2008a, character 18), Ketchum & Benson (2010, character 30).

This character is considered identical to characters describing extension of the ‘jugal’ anterior to the orbit (O’Keefe 2001, character 32) and exclusion of the maxilla from the orbit margin (Sato 2002, character 18).

**31. Prefrontal-external naris contact:** prefrontal does not participate in the margin of the external naris (0); does participate (1).

Unambiguous: *Yunguisaurus* (0->1).

DELTRAN: *Microcleidus* [node] (0->1), *Plesiosaurus* (0->1).

ACCTRAN: *Plesiopterys* + *Cryptoclidus* (1->0), Plesiosauroidea (0->1).

O’Keefe (2001, character 39), Sato (2002, character 27), Druckenmiller & Russell (2008a, character 27), Smith & Dyke (2008, character 16), Ketchum & Benson (2010, character 25), Vincent (2010, character 4).

Modified from Großmann (2007, character 7).

**32. Jugal-orbit contact:** jugal participates in orbit margin (0); jugal excluded from orbit margin by maxilla-postorbital contact (1).

Unambiguous: *Microcleidus* [node] (0->1).

O’Keefe (2001, character 31), Sato (2002, character 16), Bardet et al (1999, character 3), Großmann (2007, character 10), Ketchum & Benson (2010, character 26), Vincent (2010, character 10).

Modified from Smith & Dyke (2008, character 23).

**33. Jugal, size and anteroposterior length:** large, with horizontal long axis, extends anteriorly at least one-third of orbital length (0); short, terminates around posterior orbital margin (1); very reduced and anteroposteriorly short with vertical long axis (2).

Unambiguous: *Microcleidus* [node] (0->1).

DELTRAN: *Cryptoclidus* (0->2).

ACCTRAN: *Plesiopterys* + *Cryptoclidus* (0->2).

Modified from Bardet et al (1999, character 1), O’Keefe (2001, character 33), Gasparini et al. (2003, character 1), O’Keefe & Wahl (2003, character 20), Großmann (2007, character 11), Druckenmiller & Russell (2008a, character 15), O’Keefe & Street (2009, character 17), Ketchum & Benson (2010, character 28) (interposition of state 1).

**34. Jugal, shape of anterior margin:** tapering (0); squared off (1).

DELTRAN: *Seeleyosaurus* +++ (0->1).

ACCTRAN: Microcleididae (0->1).

New character.

**35. Postorbital, length of posterolateral process:** long, extending posteriorly for at least two-thirds of the temporal fenestra length (0); prominent, but not elongate, extending approximately one-third of temporal fenestra length (1); short or absent (2).

Unambiguous: Neoplesiosauria (0->2).

DELTRAN: *Seeleyosaurus* +++ (2->1).

ACCTRAN: Microcleididae (2->1).

Druckenmiller & Russell (2008a, character 22), Ketchum & Benson (2010, character 33).

Modified from Sato (2002, character 38), Smith & Dyke (2008, character 2).

State 0 from O’Keefe (2004, character 168), O’Keefe & Wahl (2003, character 90), O’Keefe & Street (2009, character 81).

State 2 from from Bardet et al (1999, character 2), Gasparini et al. (2003, character 2), Großmann (2007, character 9).

**36. Pineal foramen, surrounding elements:** enclosed entirely within the parietals (0); contacts the frontals anteriorly (1) (inapplicable for taxa with a closed pineal foramen or frontals that are separated along the midline by premaxilla-parietal contact).

Unambiguous: *Plesiopterys* + *Cryptoclidus* (0->1), *Yunguisaurus* (0->1).

O’Keefe (2001, character 21), O’Keefe & Wahl (2003, character 31), Druckenmiller & Russell (2008a, character 30), Smith & Dyke (2008, character 26), O’Keefe & Street (2009, character 13), Ketchum & Benson (2010, character 36), Vincent (2010, character 17).

Modified from Sato (2002, character 29), Sato (2002, character 32).

**37. Inter-squamosal suture along the dorsal midline in lateral view:** low and rounded (0); raised ~1/3 orbit height dorsally relative to skull table (1); forms anteriorly oriented vertex (2); raised abruptly and substantially dorsally relative to skull table (3).

Unambiguous: *Maresaurus* (0->1), NHMUK 49202 (0->1).

DELTRAN: *Microcleidus* *homalospondylus* + *M. brachypterygius* (0->1), *Cryptoclidus* (0->1), *Seelyosaurus* (0->1).

ACCTRAN: Microcleididae + *Plesiopterys* + *Cryptoclidus* (0->1), *M. tournemirensis* (1->0), *Plesiopterys* (1->0).

Modified from Benson (in press, character 43) (interposition of state 1).

Modified from Sato (2002, character 73), Smith & Dyke (2008, character 28), Ketchum & Benson (2010, character 43) (addition of states 1 and 3).

State 2 proposed by Cruickshank (1997).

**38. Parietal vault in dorsal view:** mediolaterally narrow, lateral surfaces weakly concave or slightly sinuous (0); expanded to approximately one-third the mediolateral width of the skull, lateral surfaces convex, forming abrupt ‘lateral angle’ of Smith & Dyke (2008) (1); strongly expanded, equal to at least half the transverse width of the posterior cranium, lateral surfaces concave (2).

Unambiguous: Neoplesiosauria (1->0).

Benson (in press, character 206).

State 1 from Smith & Dyke (2008, character 29).

State 2 probably corresponds to the ‘parietal wing’ of Sato (2002, character 33), Druckenmiller & Russell (2008a, character 32), Ketchum & Benson (2010, character 40).

**39. Parietal, sagittal crest height:** low, transversely convex (0); high, transversely compressed sheet (1); very high, forming convex dome in lateral view rising above the skull table (2); crest absent, dorsal surface of parietal broad and flat (3).

Unambiguous: *Microcleidus* [node] (0->1), Rhomaleosauridae + Neoplesiosauria (1->0).

DELTRAN: *Maresaurus* +++ (0->1), *Attenborosaurus* (0->1), *H. longirostris* (0->1), *Peloneustes* (0->2).

ACCTRAN: *Eurycleidus* +++ (0->1), *Hauffiosaurus* +*Attenborosaurus* + *Peloneustes* (0->1), *H. tomistomimus* (1->0), *Peloneustes* (1->2).

Modified from Benson (in press, character 182) (addition of state 3).

Modified from O’Keefe & Wahl (2003, character 94), O’Keefe (2008, character 32), O’Keefe & Street (2009, character 85).

**40. Parietal ornamentation adjacent to the pineal foramen:** ornamentation absent (0); ornamented with numerous ridges that radiate from the pineal foramen, but flat or slightly concave along midline (1), parietal with raised midline ridge (2); ‘parietal table’ [triangular depression between pineal foramen and sagittal crest] present (3)

Unambiguous: *Augustasaurus* (0->1), *Cryptoclidus* (0->1).

DELTRAN: *Peloneustes* (0->1).

ACCTRAN: *Attenborosaurus* + *Peloneustes* (0->1).

Benson (in press, character 37).

Modified from Ketchum & Benson (2010, character 37).

State 3 described by Druckenmiller & Russell (2008b).

**41. Parietal, anterior extension:** short or absent, parietal extends to the level of the temporal bar (0); long, parietal extends to orbital midlength or more anteriorly (1); very long, parietal extends to anterior orbit margin or more anteriorly (2).

Unambiguous: *Archaeonectrus* +++ (0->1), Pliosauridae (0->1).

DELTRAN: *Peloneustes* (1->2).

ACCTRAN: *Attenborosaurus* + *Peloneustes* (1->2).

Benson (in press, character 181).

Benson *et al.* (2011a) described how the anterior extent of the parietal varies independently from the presence of a parietal-premaxillary contact (i) and the width of the premaxilla-parietal contact (ii). These aspects of variation were scored as composite characters by (i) O’Keefe & Wahl (2003, character 8, O’Keefe & Street (2009, character 8, Ketchum & Benson (2010, character 14; and (ii) Sato (2002, character 6, Ketchum & Benson (2010, character 14

**42. Squamosal, anterior extent:** ventral to postorbital bar (0); significantly posterior to postorbital bar (1).

DELTRAN: *‘R.’ megacephalus* +++ (0->1), *Microcleidus* [node] (0->1).

ACCTRAN: *Eurycleidus* +++ (0->1), Microcleididae (0->1).

Großmann (2007, character 14).

**43. Inter-squamosal suture along the dorsal midline:** flat (0); bulbous (1) (inapplicable for taxa that lack midline squamosal contact).

Unambiguous: Microcleididae + *Plesiopterys* + *Cryptoclidus* (1->0), *M. tournemirensis* (0->1).

O’Keefe (2001, character 55), Sato (2002, character 72), Druckenmiller & Russell (2008a, character 34), Smith & Dyke (2008, character 30), Ketchum & Benson (2010, character 42).

**44. Squamosal-quadrate foramen:** absent (0); present (1).

Smith & Dyke (2008, character 31).

DELTRAN: *Rhomaleosaurus* [node] (0->1).

ACCTRAN: *Archaeonectrus* +++ (0->1).

**45. Squamosal-quadrate contact:** squamosal does not possess a long medial process covering the quadrate (0); does (1).

Unambiguous: Microcleididae + *Plesiopterys* + *Cryptoclidus* (1->0).

O’Keefe (2001, character 26), Sato (2002, character 76), O’Keefe & Wahl (2003, character 17), Druckenmiller & Russell (2008a, character 38), O’Keefe & Street (2009, character 15), Ketchum & Benson (2010, character 46).

**46. Squamosal, outline of posterior margin in lateral view:** approximately straight (0); dorsal portion inflected anterodorsally (1).

DELTRAN: *Seeleyosaurus* +++ (0->1), *Attenborosaurus* (0->1), *Thalassiodracon* (0->1).

ACCTRAN: Pliosauridae (0->1), Microcleididae (0->1), *Peloneustes* (1->0).

New character.

**47. Position of tooth row in lateral view:** collinear with the mandibular glenoid fossa (0); considerably higher than the glenoid fossa (1).

Unambiguous: Pliosauridae (1->0), Plesiosauria (0->1), *Attenborosaurus* (0->1), *Macroplata* (1->0).

O’Keefe (2001, character 98), Sato (2002, character 91), Gasparini et al. (2003, character 11), O’Keefe & Wahl (2003, character 53), Druckenmiller & Russell (2008a, character 82), O’Keefe & Street (2009, character 44), Ketchum & Benson (2010, character 94), Vincent (2010, character 41).

**48. Notochordal pit on occipital condyle:** absent (0); present (1).

Unambiguous: *Cryptoclidus* (1->0).

Sato (2002, character 79), Druckenmiller & Russell (2008a, character 66), Ketchum & Benson (2010, character 81), Vincent (2010, character 32).

**49. Occipital condyle constriction:** constricting groove around the occipital condyle is complete, exoccipital facets are separated from the occipital condyle by a groove (0); incomplete because exoccipital facets contact the occipital condyle (1); absent [even ventrally] (2).

Unambiguous: Rhomaleosauridae + Neoplesiosauria (2->1), *H. longirostris* (1->0), *M. tournemirensis* (1->2).

Sato (2002, character 78), Druckenmiller & Russell (2008a, character 69).

Modified from Bardet et al (1999, character 14), O’Keefe (2001, character 42), O’Keefe (2001, character 43), Gasparini et al. (2002, character 7), Sato (2002, character 77), O’Keefe & Wahl (2003, character 22), O’Keefe & Wahl (2003, character 23), Druckenmiller & Russell (2008a, character 68), O’Keefe & Street (2009, character 19), O’Keefe & Street (2009, character 20), Ketchum & Benson (2010, character 79), Vincent (2010, character 33) (addition of state 2).

**50.** **Basioccipital, ventral process/plate:** absent or weakly developed, ventral surface of occipital condyle essentially level with ventral surface eof basicranium (0); wide, flat, relatively smooth, plate present, demarcated from ventral surface of condyle by low step [ventral plate present] (1); very prominent, ventral plate present [sensu Druckenmiller & Russell (2008, ch. 70)] (2).

Unambiguous: *Hauffiosaurus* [node] (1->2), Rhomaleosauridae + Neoplesiosauria (0->1).

DELTRAN: *Microcleidus* [node] (1->2), *Plesiopterys* + *Cryptoclidus* (1->0), *Macroplata* (1->2), *Maresaurus* (1->2).

ACCTRAN: *Macroplata* +++ (1->2), Microcleididae (0->2), Microcleididae + *Plesiopterys* + *Cryptoclidus* (1->0), *Meyerasaurus* (2->1).

Modified from Sato (2002, character 81), Druckenmiller & Russell (2008a, character 70), Smith & Dyke (2008, character 51), Ketchum & Benson (2010, character 80).

**51. Exoccipital-opisthotic body, dorsoventral height:anteroposterior width ratio:** low and broad, ratio = 0.9–1.0 (0); 1.2–1.3 (1); tall and narrow, ratio >1.4 (2).

Unambiguous: Rhomaleosauridae (0->2), *Plesiopterys* + *Cryptoclidus* (0->1).

New character.

**52. Exoccipital, foramina in lateral surface:** one (0); two (1); three (2)

Unambiguous: NHMUK 49202 (0->1).

DELTRAN: Microcleididae + *Plesiopterys* + *Cryptoclidus* (0->1).

ACCTRAN: Plesiosauroidea (0->1).

Modified from Benson (in press, character 183) (addition of state 2).

**53. Opisthotic, paraoccipital process length relative to height of exoccipital body:** subequal (0); long, at least 1.3 times as long as body height (1); short, less than 0.7 times body height.

Unambiguous: *Macroplata* +++ (0->1), NHMUK 49202 (0->1).

DELTRAN: *Peloneustes* (0->1).

ACCTRAN: *Hauffiosaurus* +*Attenborosaurus* + *Peloneustes* (0->1).

Modified from O’Keefe (2001, character 46), Sato (2002, character 64), O’Keefe & Wahl (2003, character 24), Großmann (2007, character 21), Druckenmiller & Russell (2008a, character 61), Smith & Dyke (2008, character 49).

**54. Opisthotic, orientation of paraoccipital process relative to ventral surface of exoccipital in posterior view:** paraoccipital process oriented parallel to ventral surface of exoccipital (0); inclined dorsally (1); inclined ventrally (2)

Unambiguous: Rhomaleosauridae + Neoplesiosauria (1->2), *Cryptoclidus* (2->0).

DELTRAN: *Peloneustes* (2->0).

ACCTRAN: *Hauffiosaurus* +*Attenborosaurus* + *Peloneustes* (2->0).

Modified from O’Keefe (2001, character 48), Sato (2002, character 67), O’Keefe & Wahl (2003, character 26), Großmann (2007, character 22), Druckenmiller & Russell (2008a, character 65), O’Keefe & Street (2009, character 22), Ketchum & Benson (2010, character 77).

**55. Opisthotic, morphology of articulation with suspensorium:** anterior surface of expanded lateral end make broad contact with suspensorium (0); terminal surface of lateral end makes narrow contact with suspensorium (1).

Unambiguous: *Cryptoclidus* (0->1), NHMUK 49202 (0->1).

Modified from O’Keefe (2001, character 49), O’Keefe & Wahl (2003, character 27).

**56. Opisthotic, shaft of paraoccipital process cross section:** subcircular, dorsoventral height subequal to anteroposterior width (0); dorsoventrally flatened; anteroposterior width much greater than dorsoventral height (1).

Unambiguous: *Hauffiosaurus* [node] (0->1), *Macroplata* (0->1), NHMUK 49202 (0->1).

New character.

**57. Opisthotic, shaft seen in posteromedial view, curvature:** absent, shaft approximately straight (0); curves dorsodistally (1).

Unambiguous: *Cryptoclidus* (0->1), NHMUK 49202 (0->1).

New character.

**58. Posteromedian ridge of supraoccipital:** present (0); absent (1).

Unambiguous: Neoplesiosauria (0->1), *Plesiopterys* (1->0).

Sato (2002, character 2), Ketchum & Benson (2010, character 83).

Modified from Druckenmiller & Russell (2008a, character 72).

**59. Posteromedian process of supraoccipital:** present (0); absent (1).

DELTRAN: *Cryptoclidus* (0->1), *Plesiosaurus* (0->1).

ACCTRAN: Plesiosauroidea (0->1), *Plesiopterys* (1->0).

O’Keefe (2001, character 56), Sato (2002, character 69), Ketchum & Benson (2010, character 84).

Modified from Druckenmiller & Russell (2008a, character 72).

**60. Supraoccipital, minimum mediolateral width of exoccipital rami in posterior view:** a single ramus is substantially narrower than the foramen magnum (0); subequal to foramen magnum (1).

Unambiguous: *Stratesaurus* (1->0), *Thalassiodracon* (1->0).

DELTRAN: *Cryptoclidus* (1->0), *M. homalospondylus* (1->0).

ACCTRAN: Microcleididae + *Plesiopterys* + *Cryptoclidus* (1->0), *Plesiopterys* (0->1).

New character.

**61. Basisphenoid [parabasisphenoid] contribution to the articulation surface of the basioccipital tuberosities for contact with the pterygoids:** contributes (0); does not contribute (1).

Unambiguous: *Cryptoclidus* (0->1).

O’Keefe (2001, character 76), Sato (2002, character 80), O’Keefe & Wahl (2003, character 43), O’Keefe & Street (2009, character 34), Ketchum & Benson (2010, character 71).

**62. Basisphenoid-basioccipital connection in ventral view:** fontanelle absent (0); fontanelle present (1) .

Unambiguous: *Hauffiosaurus* [node] (0->1), *M. tournemirensis* (0->1).

Ketchum & Benson (2010, character 70).

**63. Parasphenoid, morphology of ventral surface within interpterygoid vacuity:** mediolaterally concave (0); flat (1); bears distinct midline keel (2); inapplicable, parasphenoid does not extend far into posterior interpterygoid vacuity (?).

Unambiguous: *Plesiopterys* + *Cryptoclidus* (2->1), *M. brachypterygius* (2->1), *Macroplata* (2->0), NHMUK 49202 (2->0), *‘R.’ megacephalus* (2->0).

Modified from Benson (in press, character 68), Druckenmiller & Russell (2008a, character 55), Ketchum & Benson (2010, character 68).

State 1 from O’Keefe (2008, character 43).

State 2 from O’Keefe (2001, character 71), Smith & Dyke (2008, character 48), Vincent (2010, character 25).

**64. Parasphenoid, posterior extent:** terminates within anterior one-third of interpterygoid vacuity or more anteriorly (0); terminates just anterior to basioccipital-basisphenoid contact on ventral surface of basicranium (1); underlaps basioccipital so basisphenoid-basioccipital contact is not visible in ventral view (2).

Unambiguous: Microcleididae + *Plesiopterys* + *Cryptoclidus* (0->1), Neoplesiosauria (2->0).

DELTRAN: *Peloneustes* (0->2).

ACCTRAN: *Attenborosaurus* + *Peloneustes* (0->2).

Modified from Druckenmiller & Russell (2008a, character 54), Ketchum & Benson (2010, character 69), Vincent (2010, character 28) (addition of state 2).

Modified from O’Keefe (2001, character 74), O’Keefe & Wahl (2003, character 42), O’Keefe & Street (2009, character 33) (characters describing basioccipital-parasphenoid contact).

**65. Parasphenoid, ventral surface anteriorly:** covered by pterygoids anterior to the posterior interpterygoid vacuities (0); visible through V-shaped notch in posterior pterygoid contact anterior to posterior interpterygoid vacuities (1).

Unambiguous: Plesiosauria (0->1), *Meyerasaurus* (1->0).

New character.

**66. Palate, suborbital fenestra bordered by ectopterygoid and maxilla:** absent (0); present (1).

DELTRAN: *‘R.’ megacephalus* +++ (0->1), *Peloneustes* (0->1).

ACCTRAN: Rhomaleosauridae (0->1), *Attenborosaurus* + *Peloneustes* (0->1).

O’Keefe (2001, character 82), Sato (2002, character 45), O’Keefe & Wahl (2003, character 46), Druckenmiller & Russell (2008a, character 44), Smith & Dyke (2008, character 40), O’Keefe & Street (2009, character 37), Ketchum & Benson (2010, character 55).

**67. Palate, lateral palatal fenestration bordered by palatine and pterygoid:** absent (0); present (1).

Unambiguous: *H. zanoni* (0->1), *Meyerasaurus* (0->1), NHMUK 49202 (0->1).

O’Keefe (2001, character 78), Druckenmiller & Russell (2008a, character 45), Smith & Dyke (2008, character 41), Ketchum & Benson (2010, character 56).

**68. Palate, element demarcating the anterior margin of the subtemporal fenestra**: ectopterygoid and pterygoid (0); ectopterygoid only (1).

Unambiguous: *M. brachypterygius* (0->1), R. cramptoni (0->1).

DELTRAN: *Plesiopterys* + *Cryptoclidus* (0->1), *Plesiosaurus* (0->1).

ACCTRAN: Microcleididae (1->0), Plesiosauroidea (0->1).

Druckenmiller & Russell (2008a, character 48).

**69. Palate, foramina between maxilla and vomer anterior to internal naris:** absent (0); present (1).

DELTRAN: *Peloneustes* (0->1).

ACCTRAN: *Attenborosaurus* + *Peloneustes* (0->1).

Benson (in press, character 184).

**70. Vomers, posterior extent:** extend to the internal nares (0); extend posterior to the internal nares (1).

Unambiguous: NHMUK 49202 (1->0), *Stratesaurus* (1->0).

DELTRAN: *M. brachypterygius* (1->0).

ACCTRAN: Microcleididae (1->0).

Sato (2002, character 44), Großmann (2007, character 17), Druckenmiller & Russell (2008a, character 42), Smith & Dyke (2008, character 37), Ketchum & Benson (2010, character 53), Vincent (2010, character 21).

Modified from O’Keefe (2001, character 83), O’Keefe & Wahl (2003, character 47), O’Keefe & Street (2009, character 38) (morphology of the pterygoid-vomer contact is coded separately by characters71 and 81).

**71. Pterygoid-vomer contact:** pterygoid does not separate vomers along midline (0); pterygoid separates vomers along the midline posteriorly (1).

Ketchum & Benson (2010, character 54), Vincent (2010, character 22).

Unambiguous: *Hauffiosaurus* +*Attenborosaurus* + *Peloneustes* (1->0), *Plesiosaurus* (1->0).

DELTRAN: *Meyerasaurus* +++ (1->0).

ACCTRAN: *Macroplata* +++ (1->0).

**72. Palatine-internal naris contact:** participates in the margin of the internal naris (0); does not participate (1).

Unambiguous: *‘R.’ megacephalus* (1->0).

DELTRAN: *Meyerasaurus* +++ (0->1).

ACCTRAN: *Macroplata* +++ (0->1).

O’Keefe (2001, character 79), Sato (2002, character 43), O’Keefe & Wahl (2003, character 44), Druckenmiller & Russell (2008a, character 41), Smith & Dyke (2008, character 36), O’Keefe & Street (2009, character 35), Ketchum & Benson (2010, character 51).

**73. Pterygoids, anterior interpterygoid vacuity:** absent (0); present (1).

Unambiguous: Plesiosauria (0->1), *Maresaurus* (1->0).

DELTRAN: *Hauffiosaurus* +*Attenborosaurus* + *Peloneustes* (1->0), *Microcleidus* [node] (1->0).

ACCTRAN: Pliosauridae (1->0), Microcleididae (1->0).

Bardet et al (1999, character 13), Gasparini et al. (2002, character 6), Gasparini et al. (2002, character 9), Großmann (2007, character 15), Smith & Dyke (2008, character 39), Ketchum & Benson (2010, character 59), Vincent (2010, character 23).

Modified from Druckenmiller & Russell (2008a, character 49).

Modified from O’Keefe (2001, character 60), Sato (2002, character 48), O’Keefe & Wahl (2003, character 34), O’Keefe & Street (2009, character 25) (the morphology of the anterior interpterygoid vacuity is coded by separate characters [74, 82–83]).

**74. Pterygoids, anterior interpterygoid vacuity, mediolateral width:** narrow, approximately one-fifth of combined pterygoid width at vacuity midlength (0); broad, at least one-third of combined pterygoid width at vacuity midlength (1); inapplicable, vacuity absent (?).

Unambiguous: *Meyerasaurus* (0->1), NHMUK 49202 (0->1), *Plesiopterys* + *Cryptoclidus* (0->1).

Modified from O’Keefe (2001, character 60), O’Keefe & Wahl (2003, character 34), O’Keefe & Street (2009, character 25).

**75. Pterygoids, posterior contact with basicranium:** loose, overlapping contact (0); firm sutural contact (1).

DELTRAN: Rhomaleosauridae + Neoplesiosauria (0->1).

ACCTRAN: Plesiosauria (0->1).

New character.

**76. Pterygoids, midline contact posterior to posterior interpterygoid vacuity:** absent (0); present posteriorly, but very small (1); present, pterygoid contact for more than two-thirds of their anteroposterior length posterior to posterior interpterygoid vacuity (2).

Unambiguous: *H. longirostris* (0->1), *‘R.’ megacephalus* (2->1), *Stratesaurus* (0->1).

DELTRAN: *Meyerasaurus* +++ (0->2), *Microcleidus* [node] (0->2), *Peloneustes* (0->2).

ACCTRAN: *Avalonnectes* +++ (0->2), *Attenborosaurus* + *Peloneustes* (0->2), Microcleididae (0->2).

Sato (2002, character 50).

Modified from Bardet et al (1999, character 12), Carpenter (1999, character 3), Großmann (2007, character 19), Druckenmiller & Russell (2008a, character 50), Smith & Dyke (2008, character 44), O’Keefe & Street (2009, character 26), Ketchum & Benson (2010, character 63), Vincent (2010, character 27) (interposition of state 1).

Modified from O’Keefe (2001, character 62), O’Keefe & Wahl (2003, character 35), O’Keefe (2008, character 44).

**77. Pterygoid lateral to the posterior interpterygoid vacuities:** flat (0); forms ventrolaterally directed flange with long axis oriented posteromedially (1); forms anteroposteriorly oriented trough (2); dished, marked depression bounded around its entire circumference (3).

Unambiguous: Plesiosauria (1->0).

DELTRAN: *Meyerasaurus* +++ (0->3), *Microcleidus* [node] (0->1), *Peloneustes* (0->1).

ACCTRAN: *Avalonnectes* +++ (0->3), *Attenborosaurus* + *Peloneustes* (0->1), Microcleididae (0->1).

State 1 from Sato (2002, character 54), Druckenmiller & Russell (2008a, character 51), Smith & Dyke (2008, character 45), Ketchum & Benson (2010, character 64); modified from O’Keefe (2001, character 67), Sato (2002, character 51).

States 2 and 3 from Druckenmiller & Russell (2008a, character 56), Ketchum & Benson (2010, character 65).

State 3 from O’Keefe (2001, character 67), Sato (2002, character 54), O’Keefe & Wahl (2003, character 38), O’Keefe & Street (2009, character 29).

**78. Pterygoid flange, posterior midline contact:** flanges do not contact on midline posterior to posterior interpterygoid vacuity (0); flanges contact on midline, enclosing semicircular fossa posterior to posterior interpterygoid vacuity (1); inapplicable, pterygoid flanges absent (?).

Unambiguous: *Peloneustes* (0->1).

Modified from O’Keefe (2001, character 69).

**79. Pterygoid, posterolateral portion of pterygoid:** does not form squared lappet (0); forms squared lappet that distinctly underlaps quadrate ramus of pterygoid (1).

DELTRAN: *Meyerasaurus* +++ (0->1).

ACCTRAN: *Macroplata* +++ (0->1).

O’Keefe (2001, character 58), O’Keefe & Wahl (2003, character 33), Druckenmiller & Russell (2008a, character 57), Smith & Dyke (2008, character 46), O’Keefe & Street (2009, character 24), Ketchum & Benson (2010, character 66).

**80. Posterior interpterygoid vacuities, ratio of maximum length to combined width:** short and broad, ratio = 0.7–1.2 (0); 1.3–1.6 (1); long and narrow, ratio = >2.0 (2).

Unambiguous: *Meyerasaurus* (0->1), *Pistosaurus* [skull] (0->2), *Yunguisaurus* (0->2).

DELTRAN: *Hauffiosaurus* [node] (0->1), *M. homalospondylus* (0->1), *M. tournemirensis* (0->1), *Peloneustes* (0->2).

ACCTRAN: *Attenborosaurus* + *Peloneustes* (1->2), *Hauffiosaurus* +*Attenborosaurus* + *Peloneustes* (0->1), Microcleididae (0->1), *M. brachypterygius* (1->0).

Modified from Smith & Dyke (2008, character 42).

**81. Pterygoid, anterior termination:** tapering (0); transversely broad (1); indeterminate (?; scored where a large anterior interpterygoid vacuity is present)

DELTRAN: *Meyerasaurus* +++ (0->1), Pliosauridae (0->1), *Plesiosaurus* (0->1).

ACCTRAN: Microcleididae + *Plesiopterys* + *Cryptoclidus* (1->0), Rhomaleosauridae + Neoplesiosauria (0->1), *Stratesaurus* (1->0).

Benson *et al.* (2011a).

Modified from O’Keefe (2001, character 83), O’Keefe & Wahl (2003, character 47), O’Keefe & Street (2009, character 38).

**82. Posterior border of anterior interpterygoid vacuity:** bordered by pterygoid (0); bordered by parasphenoid (1) (inapplicable in taxa that lack an anterior interpterygoid vacuity).

Unambiguous: Rhomaleosauridae (1->0).

Modified from Sato (2002, character 48).

**83. Anterior border of anterior interpterygoid vacuity:** bordered by pterygoid (0); bordered by vomer (1) (inapplicable in taxa that lack an anterior interpterygoid vacuity).

Unambiguous: *Meyerasaurus* (0->1), NHMUK 49202 (0->1), *Plesiosaurus* (0->1).

New character.

**84. Ectopterygoid/pterygoid boss/flange:** absent (0); ventrally deflected posterior margin forms flange (1); rugose ventral boss present (2).

Unambiguous: Rhomaleosauridae (0->2), *Hauffiosaurus* +*Attenborosaurus* + *Peloneustes* (0->2), *Meyerasaurus* (2->1), *Pistosaurus* [skull] (0->2).

DELTRAN: *Microcleidus* [node] (0->2).

ACCTRAN: Microcleididae (0->2).

Modified from O’Keefe (2001, character 84), Sato (2002, character 57), Druckenmiller & Russell (2008a, character 47), Ketchum & Benson (2010, character 58) (interposition of state 1 from Sato [2002, character 56]).

**85. Epipterygoid shape in lateral view:** plate-like with near-parallel sides (0); triangular (1); anteroposteriorly narrow (2).

Unambiguous: *Maresaurus* (0->1), *Avalonnectes* (0->2).

DELTRAN: *Plesiosaurus* +++ (0->2), NHMUK 49202 (0->1), *Plesiopterys* (2->1), *Thalassiodracon* (0->1).

ACCTRAN: Rhomaleosauridae (1->0), *Plesiopterys* + *Cryptoclidus* (2->1), Plesiosauroidea (1->2), Plesiosauria (0->1).

Druckenmiller & Russell (2008a, character 60), Ketchum & Benson (2010, character 73).

Modified from O’Keefe (2001, character 53), O’Keefe & Wahl (2003, character 30).

**86. Mandible-Shape of the mandible:** bowed (0); not significantly bowed (1).

Unambiguous: *Attenborosaurus* (1->0).

DELTRAN: *Meyerasaurus* +++ (1->0), *Plesiosaurus* +++ (1->0).

ACCTRAN: *Avalonnectes* +++ (1->0), Plesiosauroidea (1->0).

Sato (2002, character 85), Druckenmiller & Russell (2008a, character 75), Smith & Dyke (2008, character 60), Ketchum & Benson (2010, character 86), Vincent (2010, character 35).

Modified from O’Keefe (2001, character 86 [‘bowed maxilla’]).

**87. Mandible, symphysis length as measured by the number of teeth adjacent to the symphysis** [relative to the number of maxillary teeth]**:** number of teeth adjacent to symphysis ~0.20–0.30 maxillary tooth count (0); ratio ~0.4–0.5 (1); symphysis very short, only 1–2 teeth adjacent to symphysis (2).

Unambiguous: *Plesiopterys* + *Cryptoclidus* (0->2).

DELTRAN: *Hauffiosaurus* [node] (0->1), *Peloneustes* (0->1).

ACCTRAN: *Hauffiosaurus* +*Attenborosaurus* + *Peloneustes* (0->1), *Attenborosaurus* (1->0).

Modified from Sato (2002, character 87), Albright et al. (2007, character 8), Druckenmiller & Russell (2008a, character 79), O’Keefe (2008, character 8), Smith & Dyke (2008, character 54), Ketchum & Benson (2010, character 113), Vincent (2010, character 36) (different quantification).

Modified from O’Keefe (2001, character 89), O’Keefe & Wahl (2003, character 48), O’Keefe & Street (2009, character 39) (lateral expansion of the mandibular symphysis is coded separately here [character 88]).

**88. Mandible, shape of the mandibular symphysis in ventral view:** tapers anteriorly (0); laterally expanded (1).

Unambiguous: *Hauffiosaurus* [node] (1->0), Plesiosauria (0->1).

DELTRAN: *Plesiosaurus* +++ (1->0).

ACCTRAN: Plesiosauroidea (1->0).

Druckenmiller & Russell (2008a, character 78), Smith & Dyke (2008, character 56), Ketchum & Benson (2010, character 91).

Modified from O’Keefe (2001, character 89), O’Keefe & Wahl (2003, character 48), O’Keefe & Street (2009, character 39).

**89. Dentary, structure along the ventral surface of the mandibular symphysis:** no ventral elaboration (0); forms raised ventral platform or sharp keel/ridge adjacent to symphysis (1); forms raised ridge adjacent and posterior to the symphysis (2).

Unambiguous: *H. longirostris* (0->1), *Peloneustes* (0->1).

DELTRAN: *Macroplata* +++ (0->1).

ACCTRAN: Rhomaleosauridae (0->1).

Druckenmiller & Russell (2008a, character 80), Ketchum & Benson (2010, character 92).

Modified from O’Keefe (2001, character 88), Smith & Dyke (2008, character 57), Vincent (2010, character 38) (addition of state 2).

**90. Coronoid, length and morphology:** short, extending less than half mandibular length (0); long, approaching or participating in symphysis (1); small, superficial element that is often disarticulated and thus not preserved, but represented by an articular fact on the surangular (2).

DELTRAN: *Cryptoclidus* (1->2).

ACCTRAN: Plesiosauroidea (1->2).

Modified from Druckenmiller & Russell (2008a, character 84), Ketchum & Benson (2010, character 90) (‘absence’ of coronoid in state 2 replaced by small size and likely disarticulation).

Modified from Albright et al. (2007, character 10), O’Keefe (2008, character 10), Ketchum & Benson (2010, character 89) (coronoid participation in symphysis).

Modified from O’Keefe (2001, character 94) (long lingual process of coronoid).

Modified from O’Keefe (2001, character 93), Sato (2002, character 93), O’Keefe & Wahl (2003, character 50), O’Keefe & Street (2009, character 41) (‘absence’ of coronoid).

**91. Mandible, length of retroarticular process:** short (0), long (1).

Unambiguous: *Archaeonectrus* (0->1).

DELTRAN: *Meyerasaurus* +++ (1->0).

ACCTRAN: *Macroplata* +++ (1->0).

O’Keefe & Wahl (2003, character 92), O’Keefe & Street (2009, character 83).

Modified from Sato (2002, character 86).

**92. Mandible, rounded medial flange formed by articular and prearticular anterior to the glenoid fossa in dorsal view:** present (0); absent (1); absent but anterior part of outline of glenoid in ventral view appears ‘squared-off’ (2).

Unambiguous: *Macroplata* +++ (1->2), *R. thorntoni* (2->1).

DELTRAN: *Peloneustes* (1->0).

ACCTRAN: *Attenborosaurus* + *Peloneustes* (1->0).

Modified from Ketchum & Benson (2010, character 102 (addition of state 2).

**93. Mandible, prominent longitudinal trough occupies much of the lateral surface anterior to the glenoid** [dentary, angular, surangular]**:** absent (0); present, bounded ventrally by robust longitudinal, ventrolateral ridge (1).

Unambiguous: Rhomaleosauridae (0->1), *Hauffiosaurus* [node] (0->1), *‘R.’ megacephalus* (1->0).

Benson *et al.* (2011b, character 180).

**94. Mandible, retroarticular process, dorsoventral orientation of long axis:** posterodorsal (0); posteroventral or subhorizontal (1).

DELTRAN: *Meyerasaurus* (0->1), NHMUK 49202 (0->1), *Seelyosaurus* (0->1), *Stratesaurus* (0->1).

ACCTRAN: *Maresaurus* +++ (1->0), Microcleididae (0->1), Neoplesiosauria (1->0), Plesiosauria (0->1).

New character.

**95. Mandible, retroarticular process, mediolateral orientation of long axis:** directly posterior, in line with long axis of mandibular ramus (0); inflected slightly posteromedially (1).

Unambiguous: Plesiosauria (1->0).

New character.

**96. Mandible, dorsal rim of 'lingual mandibular fenestra'** **formed by:** prearticular (0); splenial (1); at prearticular-splenial contact (2); inapplicable, lingual mandibular fenestra absent (?).

DELTRAN: *Meyerasaurus* +++ (0->2), *Peloneustes* (0->1).

ACCTRAN: *Macroplata* +++ (0->2), *Hauffiosaurus* +*Attenborosaurus* + *Peloneustes* (0->1).

New character.

**97. Splenial, participation in mandibular symphysis:** does not participate (0); participates (1)

Unambiguous: *Plesiopterys* + *Cryptoclidus* (1->0).

Sato (2002, character 88), Druckenmiller & Russell (2008a, character 76), Smith & Dyke (2008, character 59), Ketchum & Benson (2010, character 87), Vincent (2010, character 37).

Modified from O’Keefe (2001, character 90), O’Keefe & Wahl (2003, character 49), Albright et al. (2007, character 9), O’Keefe (2008, character 9), O’Keefe & Street (2009, character 40) (participation of the angular in the symphysis is coded separately here by character 99).

**98. Angular, relative length and participation in mandibular symphysis:** long, extending more than half mandibular length, but does not participate in the symphysis (0); short, extends less than half mandibular length (1); very long, participates in symphysis (2).

DELTRAN: Microcleididae + *Plesiopterys* + *Cryptoclidus* (0->1).

ACCTRAN: Plesiosauroidea (0->1).

State 1 from Sato (2002, character 99).

State 2 from Sato (2002, character 89), Druckenmiller & Russell (2008a, character 77), Ketchum & Benson (2010, character 88).

Modified from O’Keefe (2001, character 90), O’Keefe & Wahl (2003, character 49), Albright et al. (2007, character 9), O’Keefe (2008, character 9), O’Keefe & Street (2009, character 40).

**99. Surangular, dorsal/dorsomedial fossa and longitudinal crest on medial surface anterior to glenoid:** prominent longitudinal crest forms ventral margin of deep, dorsomedially facing surangular fossa (0); prominent longitudinal crest forms medial margin of mediolaterally expanded dorsal surface of surangular bearing shallow, dorsally facing fossa (1); crest and surangular fossa weak or absent, dorsal portion of surangular 'blade-like' (2).

Unambiguous: Neoplesiosauria (0->2).

DELTRAN: *Peloneustes* (2->1).

ACCTRAN: *Attenborosaurus* + *Peloneustes* (2->1).

State 0 from Druckenmiller & Russell (2008a, character 86), Ketchum & Benson (2010, character 97).

State 1 from Druckenmiller & Russell (2008a, character 87), Ketchum & Benson (2010, character 98).

**100. Surangular foramen:** present (0); absent (1).

DELTRAN: *Peloneustes* (0->1).

ACCTRAN: *Hauffiosaurus* +*Attenborosaurus* + *Peloneustes* (0->1).

Ketchum & Benson (2010, character 99).

In many basal plesiosaurians a large foramen peforates the surangular anteroventrally, extending anteriorly as a horizontal groove (e.g. Cruickshank 1994b, fig. 9b; Benson *et al.* 2011a, fig. 2A,C,E).

**101. Surangular foramen:** visible in lateral view (0); fully or partly obscured by overlapping dentary when mandible is articulated (1); inapplicable, foramen absent (?).

DELTRAN: Microcleididae + *Plesiopterys* + *Cryptoclidus* (0->1).

ACCTRAN: Plesiosauroidea (0->1).

New character.

**102. Articular, deep anteroposteriorly oriented cleft** **[notch]** **posterior to glenoid:** absent (0); present (1); cleft absent, but dorsal surface is strongly concave mediolaterally (2)

Unambiguous: *Hauffiosaurus* [node] (0->2), *Yunguisaurus* (0->1).

Modified from Ketchum & Benson (2010, character 104 (addition of state 2).

**103. Dentition, number of premaxillary teeth:** four (0); five (1); six (2); seven or more (3).

Unambiguous: *Hauffiosaurus* [node] (1->3), *Macroplata* (1->2), NHMUK 49202 (1->0), *Peloneustes* (1->2), *Thalassiodracon* (1->0), *Yunguisaurus* (1->3).

DELTRAN: *Cryptoclidus* (1->2).

ACCTRAN: *Plesiopterys* + *Cryptoclidus* (1->2).

Modified from Bardet et al (1999, character 15), Carpenter (1999, character 11), Sato (2002, character 107), Gasparini et al. (2002, character 8), Gasparini et al. (2003, character 13), O’Keefe & Wahl (2003, character 56), Albright et al. (2007, character 1), Großmann (2007, character 23), Druckenmiller & Russell (2008a, character 93), O’Keefe (2008, character 1), Smith & Dyke (2008, character 4), O’Keefe & Street (2009, character 47), Ketchum & Benson (2010, character 110), Vincent (2010, character 42) (different quantification).

**104. Dentition, regularity of premaxillary dentition:** homodont (0); heterodont (1).

Unambiguous: *M. tournemirensis* (0->1), *Peloneustes* (0->1).

DELTRAN: *R. thorntoni* (0->1).

ACCTRAN: *Archaeonectrus* +++ (0->1).

Sato (2002, character 104), Druckenmiller & Russell (2008a, character 89), Ketchum & Benson (2010, character 105), Vincent (2010, character 43).

Modified from O’Keefe (2001, character 101) (dentary heterodonty not considered here).

Modified from Bardet et al (1999, character 17), Carpenter (1999, character 10), Gasparini et al. (2003, character 14), Großmann (2007, character 24) (maxillary heterodonty coded separately by character 105 here).

**105. Dentition, regularity of maxillary dentition:** homodont (0); heterodont (1).

Unambiguous: *‘R.’ megacephalus* (1->0), *Stratesaurus* (1->0).

DELTRAN: *Plesiosaurus* +++ (1->0).

ACCTRAN: Plesiosauroidea (1->0).

O’Keefe (2001, character 102), Sato (2002, character 105), O’Keefe & Wahl (2003, character 54), Albright et al. (2007, character 3), Druckenmiller & Russell (2008a, character 90), O’Keefe (2008, character 3), O’Keefe & Street (2009, character 45), Ketchum & Benson (2010, character 106), Vincent (2010, character 44).

**106. Teeth, enamel ‘striations’** **(grooves):** present (0); absent (1).

Unambiguous: Plesiosauria (0->1).

Ketchum & Benson (2010, character 108).

**107.Teeth, form of apicobasally extending enamel ridges:** coarse (0); fine (1); absent (2).

Unambiguous: Plesiosauria (2->1).

DELTRAN: *Rhomaleosaurus* [node] (1->0), *Meyerasaurus* (1->0), *Peloneustes* (1->0), *‘R.’ megacephalus* (1->0).

ACCTRAN: *Meyerasaurus* +++ (1->0), *Attenborosaurus* + *Peloneustes* (1->0), *Archaeonectrus* (0->1), *Maresaurus* (0->1).

Ketchum & Benson (2010, character 107).

States 1 and 2 from Druckenmiller & Russell (2008a, character 91).

State 2 modified from Bardet et al (1999, character 18), O’Keefe (2001, character 105), Sato (2002, character 102), Gasparini et al. (2002, character 9), Gasparini et al. (2003, character 16).

Characters describing other aspects of tooth ornamentation have also been used in previous studies but are not included in the current analysis:

Spacing of enamel ridges: Smith & Dyke (2008, character 53).

Distribution/symmetry of enamel ridges: O’Keefe (2001, character 105), Sato (2002, character 102), Großmann (2007, character 25).

Prominence/reduction of ridges: Bardet et al (1999, character 18), Sato (2002, character 102), Gasparini et al. (2003, character 16), Gasparini *et al.* (2002, character 9), Großmann (2007, character 26).

The presence of carinae: Sato (2002, character 101), (Cruickshank 1997).

**108. Dentition, number of maxillary teeth:** 12–17 (0); 20–25 (1); >28 (2).

Unambiguous: *‘R.’ megacephalus* +++ (1->2), *Hauffiosaurus* +*Attenborosaurus* + *Peloneustes* (1->2), *Augustasaurus* (0->1), *R. thorntoni* (2->1).

DELTRAN: *Macroplata* +++ (0->1), *Seeleyosaurus* +++ (1->0), Neoplesiosauria (0->1).

ACCTRAN: Microcleididae (1->0), Rhomaleosauridae + Neoplesiosauria (0->1), *Stratesaurus* (1->0).

Modified from Albright et al. (2007, character 2), Druckenmiller & Russell (2008a, character 94), Ketchum & Benson (2010, character 111) (different quantification).

Modified from Bardet et al (1999, character 16), Sato (2002, character 111), Gasparini et al. (2003, character 15), Albright et al. (2007, character 4), O’Keefe (2008, character 4) (characters coding the dentary tooth count).

**109. Premaxilla, first alveolus:** not significantly smaller than third alveolus (0); less than half the diameter of third alveolus (1).

DELTRAN: Microcleididae + *Plesiopterys* + *Cryptoclidus* (0->1).

ACCTRAN: Plesiosauroidea (0->1).

Sato (2002, character 108); modified from Benson (in press, character 202).

**110. Axial rib articulation:** articulates solely with the axial centrum (0); articulates partially with the atlantal centrum (1).

DELTRAN: *Cryptoclidus* (0->1), *H. tomistomimus* (0->1), *Peloneustes* (0->1).

ACCTRAN: Neoplesiosauria (0->1), *H. zanoni* (1->0), *Thalassiodracon* (1->0).

O’Keefe (2001, character 109), O’Keefe & Wahl (2003, character 58), O’Keefe & Street (2009, character 49), Ketchum & Benson (2010, character 117).

**111. Atlantal centrum morphology:** excluded from lateral rim of atlantal cup by contact between the atlantal intercentrum and neural arches (0); participtes in rim of atlantal cup (1).

DELTRAN: *Cryptoclidus* (0->1).

ACCTRAN: Plesiosauroidea (0->1).

Sato (2002, character 112), Druckenmiller & Russell (2008a, character 96), Ketchum & Benson (2010, character 114).

Modified from O’Keefe (2001, character 110), O’Keefe & Wahl (2003, character 59), O’Keefe & Street (2009, character 50) (morphology of axial intercentrum not included in character description here).

**112. Atlas-axis complex, hypophyseal ridge:** absent or low bulge (0); present (1).

DELTRAN: *Cryptoclidus* (0->1).

ACCTRAN: Microcleididae + *Plesiopterys* + *Cryptoclidus* (0->1).

Druckenmiller & Russell (2008a, character 97), Ketchum & Benson (2010, character 115).

**113. Atlas rib/rib facet or rib-like projection:** absent (0); rib present, contacts atlas via a distinct rib facet (1); posteroventral projection resembling a fused atlantal rib, but lacking evidence of a rib facet (2).

Unambiguous: Rhomaleosauridae + Neoplesiosauria (0->2), *Augustasaurus* (0->2).

DELTRAN: *Cryptoclidus* (2->1).

ACCTRAN: Plesiosauroidea (2->1).

Modified from Sato (2002, character 116), Druckenmiller & Russell (2008a, character 98), Ketchum & Benson (2010, character 116) (addition of state 2).

**114. Axial neural spine:** transversely narrow (0); transversely broad (1).

DELTRAN: *H. tomistomimus* (0->1).

ACCTRAN: *Hauffiosaurus* +*Attenborosaurus* + *Peloneustes* (0->1).

Benson *et al.* (2011, character 188).

**115. Number of cervical vertebrae** (excluding ‘pectoral’ vertebrae when ribs are not preserved; but including all vertebrae anterior to the pectoral girdle, or with ‘cervical’ ribs where specimens are more complete)**:** 30–36 (0); 24–29 (1); >37 (2); <23 (3).

Unambiguous: Pliosauridae (2->0), *Bobosaurus* (2->3), *Cryptoclidus* (2->0), *M. brachypterygius* (2->0), *Peloneustes* (0->3), *Seelyosaurus* (2->0).

DELTRAN: *Macroplata* +++ (2->1).

ACCTRAN: Rhomaleosauridae (2->1).

Modified from Bardet et al (1999, character 19), Carpenter (1999, characters 12–15), O’Keefe (2001, character 2), Sato (2002, characters 117–118), Gasparini et al. (2003, character 17), O’Keefe & Wahl (2003, characters 2, 60), Albright et al. (2007, character 13), Großmann (2007, character 27), Druckenmiller & Russell (2008a, character 99), O’Keefe (2008, character 13), O’Keefe & Street (2009, character 51), Ketchum & Benson (2010, character 118).

Published counts follow different criteria for identification of cervical vertebrae in different studies, and so differ from some counts included in our matrix. For example, although Sato *et al.* (2010) described 49 cervical vertebrae in *Yunguisaurus*. They included ‘pectorals’ in this count. We instead define cervical vertebrae as all vertebrae anterior to the pectoral girdle (including the clavicle-interclavicle complex) and count 44 in *Yunguisaurus*. Sollas (1881) counted 38 cervical vertebrae in *Attenborosaurus*, but included in this count two vertebrae with ‘dorsalised’ ribs that are counted as dorsals in the present study (i.e. *Attenborosaurus* is considered to have 36 cervical vertebrae).

**116. Proportions of the anterior cervical centra:** approximately as long as high (0); longer than high (1); shorter than high [length~1/2 height] (2).

Unambiguous: *Meyerasaurus* +++ (0->2), *Microcleidus* [node] (0->1), *Archaeonectrus* (2->0), *Bobosaurus* (0->2), *Macroplata* (0->2), *Peloneustes* (0->2), *Pistosaurus* [postcranium] (0->1), *Plesiopterys* (0->1).

O’Keefe (2001, character 112), Sato (2002, character 120), O’Keefe & Wahl (2003, character 61), Druckenmiller & Russell (2008a, character 102), O’Keefe & Street (2009, character 52), Ketchum & Benson (2010, character 120).

Modified from Bardet et al (1999, character 20), Vincent (2010, character 21) (addition of state 0).

Modified from Großmann (2007, character 28) (addition of state 2).

Modified from Albright et al. (2007, character 12), O’Keefe (2008, character 12), Smith & Dyke (2008, character 61) (interposition of state 1).

**117. Cervical centra, articular surfaces:** strongly concave (0); gently concave, nearly flat (1).

DELTRAN: *M. homalospondylus* (0->1), *Peloneustes* (0->1).

ACCTRAN: *Attenborosaurus* + *Peloneustes* (0->1), *Microcleidus* *homalospondylus* + *M. brachypterygius* (0->1).

Bardet et al (1999, character 21), Gasparini et al. (2003, character 18), Großmann (2007, character 29), Druckenmiller & Russell (2008a, character 106).

Modified from Ketchum & Benson (2010, character 124) (‘gently convex’ [mainly scored for pliosaurids] and ‘flat’ [mainly scored for elasmosaurids] states describe near-identical conditions [state 1 here]).

Modified from Sato (2002, character 128).

**118. Lateral surface of anterior cervical centra:** longitudinal ridge absent (0); present (1).

Unambiguous: *Microcleidus* [node] (0->1).

Bardet et al (1999, character 22), Carpenter (1999, character 17), O’Keefe (2001, character 115), Gasparini et al. (2003, character 19), Großmann (2007, character 30), Druckenmiller & Russell (2008a, character 103), Ketchum & Benson (2010, character 121).

Modified from Sato (2002, characters 123–124) (separate characters for anterior and posterior cervical vertebrae lumped into a single character here).

**119. Anterior cervical neural spines, morphology:** curve posterodorsally (0); inclined straight posterodorsally (1); inflected anterodorsally (2); inapplicable in some pistosaurians that have extremely low neural spines (?).

Unambiguous: Microcleididae + *Plesiopterys* + *Cryptoclidus* (0->1), *Pistosaurus* [postcranium] (0->1).

DELTRAN: *H. zanoni* (0->1), *Peloneustes* (0->1).

ACCTRAN: *Hauffiosaurus* +*Attenborosaurus* + *Peloneustes* (0->1), *Attenborosaurus* (1->0).

Modified from Sato (2002, character 135) (more states and different qualitative description here).

Modified from O’Keefe (2001, character 125), O’Keefe & Wahl (2003, character 70), Druckenmiller & Russell (2008a, character 111), Smith & Dyke (2008, character 70), O’Keefe & Street (2009, character 61), Vincent (2010, character 50) (these studies coded a single character for all cervical vertebrae; we code separate characters for anterior and posterior [ch. 120] cervical vertebrae; and include more states).

**120. Posterior cervical neural spines, morphology:** curve posterodorsally (0); inclined straight posterodorsally (1); inflected anterodorsally (2); inapplicable in some pistosaurians that have extremely low neural spines (?).

Unambiguous: Rhomaleosauridae (1->0), *Seelyosaurus* (1->2), *Thalassiodracon* (1->0).

DELTRAN: *Eretmosaurus* (1->0), R. cramptoni (0->1), *Westphaliasaurus* (1->0).

ACCTRAN: *Rhomaleosaurus* [node] (0->1), *Seeleyosaurus* +++ (0->1), Microcleididae (1->0).

Modified from Sato (2002, character 136) (more states and different qualitative description here).

Modified from O’Keefe (2001, character 125), O’Keefe & Wahl (2003, character 70), Druckenmiller & Russell (2008a, character 111), Smith & Dyke (2008, character 70), O’Keefe & Street (2009, character 61), Vincent (2010, character 50).

**121. Posterior cervical neural spines, height relative to centrum:** substantially shorter than centrum (0); subequal (1); substantially taller [equal to or greater than 1.2 times centrum height] (2).

Unambiguous: *Plesiosaurus* +++ (1->2), *Archaeonectrus* (2->1), *Peloneustes* (1->2).

DELTRAN: *Avalonnectes* +++ (1->2), Rhomaleosauridae + Neoplesiosauria (0->1).

ACCTRAN: *Macroplata* +++ (1->2), Plesiosauria (0->1).

Modified from Ketchum & Benson (2010, character 130) (character restricted to posterior cervical neural spines; anterior neural spines coded by character 131)

**122. Rib facets of the anterior–middle cervical vertebrae:** rib facets broadly separated (0); two co-joined rib facets (1); one rib facet (2).

Unambiguous: *Bobosaurus* (0->1), *Cryptoclidus* (1->2).

DELTRAN: Rhomaleosauridae + Neoplesiosauria (0->1), NHMUK 49202 (0->2).

ACCTRAN: Plesiosauria (0->1), NHMUK 49202 (1->2).

Benson (in press, character 125).

Modified from Druckenmiller & Russell (2008a, character 107), Ketchum & Benson (2010, character 125) (these studies coded one character for all cervical vertebrae; we use separate characters for anterior and posterior [ch. 123] cervical vertebrae).

Modified from O’Keefe (2001, character 117), Sato (2002, character 144), Smith & Dyke (2008, character 72), Vincent (2010, character 51).

An anterior cervical of *Maresaurus* was reported by Gasparini (1997) as having a single-headed rib facet. However, other anterior centra have double-headed facets. This is interesting but the taxon is scored as ‘1’ for the present time.

**123. Rib facets of the posterior cervical vertebrae:** rib facets broadly separated (0); two co-joined rib facets (1); one rib facet (2).

Unambiguous: Microcleididae (1->0), *Bobosaurus* (0->1), *Cryptoclidus* (1->2), *Peloneustes* (1->2).

DELTRAN: Rhomaleosauridae + Neoplesiosauria (0->1).

ACCTRAN: Plesiosauria (0->1).

Modified from Benson (in press, character 210).

Modified from Druckenmiller & Russell (2008a, character 107), Ketchum & Benson (2010, character 125) (these studies coded a single character for all cervical vertebrae; we use separate characters for anterior [ch. 122] and posterior cervical vertebrae).

Modified from O’Keefe (2001, character 117), Sato (2002, character 144), Smith & Dyke (2008, character 72), Vincent (2010, character 51).

**124. Anterior process of cervical ribs:** present forming a prominent elongate projection (0); present but dorsoventrally thick, anteroposteriorly low, and rounded (1: new state); absent in most cervical ribs, small processes may be present in anterior cervical ribs (2).

Unambiguous: *Plesiopterys* + *Cryptoclidus* (0->2).

DELTRAN: *Peloneustes* (0->1).

ACCTRAN: *Attenborosaurus* + *Peloneustes* (0->1).

Ketchum & Benson (2011, character 134).

Modified from O’Keefe (2001, character 123), Sato (2002, character 146), O’Keefe & Wahl (2003, character 68), Druckenmiller & Russell (2008a, character 115), Smith & Dyke (2008, character 71), O’Keefe & Street (2009, character 59), Ketchum & Benson (2010, character 134), Vincent (2010, character 52).

**125. Cervical zygapophyses, combined width:** broader than the centrum (0), subequal to the centrum (1), or distinctly narrower than the centrum (2).

Unambiguous: *Eurycleidus* +++ (1->0), Plesiosauria (0->1), *Bobosaurus* (0->1).

DELTRAN: *Microcleidus* [node] (1->2).

ACCTRAN: *Seeleyosaurus* +++ (1->2).

O’Keefe (2001, character 120), O’Keefe & Wahl (2003, character 65), Druckenmiller & Russell (2008a, character 108), Ketchum & Benson (2010, character 127).

Smith & Dyke (2008, character 63) (addition of state 2).

Modified from O’Keefe & Street (2009, character 56) (addition of state 0).

**126. Cervical centra, median ventral surface:** approximately flat or convex (0); bears a rounded midline ridge [cf. Tarlo 1960] (1); or bears a sharp keel (2).

Unambiguous: *Plesiosaurus* +++ (2->0), *Eretmosaurus* (0->2), *Pistosaurus* [postcranium] (2->0), *R. thorntoni* (2->0), *Yunguisaurus* (2->0).

DELTRAN: *Hauffiosaurus* [node] (2->0), *Peloneustes* (2->1).

ACCTRAN: *Attenborosaurus* + *Peloneustes* (0->1), *Hauffiosaurus* +*Attenborosaurus* + *Peloneustes* (2->0).

Druckenmiller & Russell (2008a, character 105).

State 2 from O’Keefe (2001, character 114), O’Keefe & Wahl (2003, character 63), Smith & Dyke (2008, characters 64, 67), O’Keefe & Street (2009, character 54).

Modified from Sato (2002, characters 125–126) (separate characters for anterior and posterior cervical vertebrae lumped together here).

Modified from Ketchum & Benson (2010, character 123) (paired ventral ridges lateral to the midline are coded separately by character 127 here).

The presence of a sharp ventral keel (state 2) was only scored when present in most cervical vertebrae, not just anterior cervical vertebrae.

**127. Cervical centra, paired lateral ridges on ventral surface:** absent (0), present (1).

DELTRAN: *Microcleidus* *homalospondylus* + *M. brachypterygius* (0->1).

ACCTRAN: *Seeleyosaurus* +++ (0->1).

Modified from Ketchum & Benson (2010, character 123).

**128. Cervical zygapophyses, orientation:** horizontal (0); dorsomedially facing (1).

Unambiguous: Plesiosauria (0->1).

New character.

**129. Cervical vertebrae, medial contact of the left and right prezygapophyses:** absent, zygapophyseal spacing subequal to width of neural canal (0); contact present at bases (1); contact present for entire anteroposterior length (2).

Unambiguous: *Plesiosaurus* (0->1).

DELTRAN: *Cryptoclidus* (0->2).

ACCTRAN: *Plesiopterys* + *Cryptoclidus* (0->2).

Benson (in press, character 128).

Modified from Sato (2002, character 132), Druckenmiller & Russell (2008a, character 109), Ketchum & Benson (2010, character 128) (interposition of state 1).

**130.** **Cervical zygapophyseal facets:** planar (0); transversely concave/convex (1).

DELTRAN: *Cryptoclidus* (0->1), *Plesiosaurus* (0->1).

ACCTRAN: Microcleididae (1->0), Plesiosauroidea (0->1).

New character.

**131. Cervical vertebrae, proportions of anterior cervical neural spines:** taller than their anteroposterior length (0); longer than tall (1); anteroposteriorly short and 'rod-like', approximately as long anteroposteriorly as the transverse width (2).

Unambiguous: Plesiosauria (1->0), *Bobosaurus* (1->0).

Modified from Druckenmiller & Russell (2008a, character 112) (addition of state 2).

Modified from Ketchum & Benson (2010, character 130) (character restricted to anterior cervical neural spines; posterior cervical neural spines coded by character 121).

**132. Cervical vertebrae, shape of neurocentral suture in anterior–middle cervical vertebrae:** rounded, ventrally convex and located dorsally (0); V-shaped (1); extends far ventrally so that neural arch contacts dorsal part of rib facet (2); extends ventrally approximately one=third of centrum height, but is evenly convex and does not contact the rib facet (3).

Unambiguous: *Avalonnectes* +++ (3->1), *Attenborosaurus* + *Peloneustes* (2->0), Pliosauridae (3->2), Plesiosauria (0->3), *Maresaurus* (1->3).

DELTRAN: *Seeleyosaurus* +++ (3->0), *Plesiopterys* + *Cryptoclidus* (3->0).

ACCTRAN: Microcleididae + *Plesiopterys* + *Cryptoclidus* (3->0), *Westphaliasaurus* (0->3).

New character.

State 2 from Ketchum & Benson (2010, character 126).

State ‘2’ was originally described as a synapomorphy of *Hauffiosaurus* (Benson *et al.* 2011). However, it is also present in *Thalassiodracon* (NHMUK 2020* [14551]).

**133. Cervical centra, proportional width:** mediolateral width subequal to height or less (0); at least 1.2 times are wide mediolaterally as high dorsoventrally (1).

Unambiguous: *Hauffiosaurus* +*Attenborosaurus* + *Peloneustes* (1->0), *Archaeonectrus* (0->1), *Augustasaurus* (1->0), *Cryptoclidus* (1->0), *Eretmosaurus* (1->0).

DELTRAN: *Eurycleidus* +++ (1->0), *Macroplata* (1->0).

ACCTRAN: *Macroplata* +++ (1->0), *Avalonnectes* (0->1).

New character.

**134. Anterior cervical centra, small, semi-oval ‘lip’ extends ventrally from anterior articular surface:** no (0); yes (1).

Unambiguous: NHMUK 49202 (0->1), *Stratesaurus* (0->1).

DELTRAN: *Peloneustes* (0->1).

ACCTRAN: *Attenborosaurus* + *Peloneustes* (0->1).

Tarlo (1960).

Ketchum & Benson (2011, character 189).

Vincent (2011) described the ventral ridge of the anterior cervical centra as extending further ventrally than the anterior articular surface in *Hauffiosaurus zanoni*, and likened this to the condition in pliosaurids (Tarlo 1960). However, the ‘lip’ in pliosaurids is a ventral extension of the actual articular surface, and is present even in taxa that lack the ventral ridge (e.g. *Liopleurodon*). This is distinct from the condition in *H. zanoni*, which is scored as ‘0’ for this character.

**135. Posterior cervical rib facets:** face laterally (0); or posterolaterally (1).

Unambiguous: *Pistosaurus* [postcranium] (0->1).

DELTRAN: *Microcleidus* [node] (0->1), *Eretmosaurus* (0->1), *Plesiopterys* (0->1).

ACCTRAN: Microcleididae + *Plesiopterys* + *Cryptoclidus* (0->1), *Cryptoclidus* (1->0), *Westphaliasaurus* (1->0).

New character.

**136. Middle­–posterior dorsal transverse processes, distal articular facet:** dorsoventrally tall oval, perhaps composed of two weakly divided rib facets (0); composed of only a single subcircular facet (1).

Unambiguous: *Peloneustes* (1->0), *Pistosaurus* [postcranium] (1->0), R. cramptoni (1->0), *Yunguisaurus* (1->0).

Modified from Druckenmiller & Russell (2008a, character 116), Smith & Dyke (2008, character 73), Ketchum & Benson (2010, character 136), Vincent (2010, character 54), Ketchum & Benson (2011, character 192).

**137. Dorsal neural spines, height:** less than or equal to the height of the centrum (0); conspicuously taller than the centrum (1); more than twice as tall as the centrum (2).

Unambiguous: *Seeleyosaurus* +++ (1->2), *Bobosaurus* (1->2).

DELTRAN: R. cramptoni (1->0).

ACCTRAN: *Rhomaleosaurus* [node] (1->0).

Ketchum & Benson (2011, character 137).

Modified from Druckenmiller & Russell (2008a, character 117), Vincent (2010, character 55) (addition of state 2).

Modified from O’Keefe (2001, character 129).

**138. Number of dorsal vertebrae:** 17–19 (0); 20–23 (1); >26 (2).

Unambiguous: *Rhomaleosaurus* [node] (1->2), *Seeleyosaurus* +++ (1->0), *Bobosaurus* (1->2), *Meyerasaurus* (1->2), *Avalonnectes* (1->0), *Yunguisaurus* (1->2).

Modified from Sato (2002, character 119), Ketchum & Benson (2010, character 135).

**139. Number of pectoral vertebrae:** 2–4 (0); 5–7 (1).

Unambiguous: *Microcleidus* *homalospondylus* + *M. brachypterygius* (0->1), *Peloneustes* (0->1), *Pistosaurus* [postcranium] (1->0).

DELTRAN: Pliosauridae (1->0), Microcleididae + *Plesiopterys* + *Cryptoclidus* (1->0).

ACCTRAN: Neoplesiosauria (1->0), *Plesiosaurus* (0->1).

New character.

‘Pectoral’ vertebrae are those in which the rib facet comprises portions of both the centrum and neural arch (Seeley 1874). They can form part of the posterior cervical or anterior dorsal series and this varies among different taxa (e.g. Benson *et al.* 2011a, table 2).

**140. Dorsal neural arch height:** tall, base of transverse process located dorsal to midheight of neural canal (0); short, transverse process adjacent to neural canal (1).

Unambiguous: *Bobosaurus* (0->1).

DELTRAN: Rhomaleosauridae + Neoplesiosauria (0->1).

ACCTRAN: Plesiosauria (0->1).

O’Keefe (2001, character 127).

**141. Dorsal transverse processes, orientation in middle dorsal region:** approximately horizontal [laterally directed] (0); inclined significantly dorsolaterally (1).

Unambiguous: *Attenborosaurus* + *Peloneustes* (0->1).

DELTRAN: *Plesiosaurus* +++ (0->1).

ACCTRAN: Plesiosauroidea (0->1).

New character.

**142. Dorsal neural spines, strong anteroposterior constriction at base:** absent (0); present (1).

Unambiguous: *Avalonnectes* +++ (0->1), *Microcleidus* [node] (0->1), *Pistosaurus* [postcranium] (0->1).

DELTRAN: *H. tomistomimus* (0->1).

ACCTRAN: *Hauffiosaurus* [node] (0->1).

Benson (in press, character 213).

**143. Dorsal neural spines, symmetry:** symmetrical and with consistent morphology throughout series (0); alternating asymmetrical morphology (1).

Unambiguous: *Archaeonectrus* +++ (1->0), *Avalonnectes* +++ (0->1).

Modified from Benson (in press, character 211).

**144. Dorsal neural spines, mediolateral width of apices in mid–posterior dorsal neural spines:** unexpanded, transversely narrow relative to anteroposterior width (0); mediolaterally thick, subequal to anteroposterior width (1); alternate spines expanded laterally to one side (2).

Unambiguous: *Archaeonectrus* +++ (2->1), *Avalonnectes* +++ (0->2).

Modified from Benson (in press, character 211).

**145. Posteriormost dorsal neural spines, orientation:** dorsal or posterodorsal (0); anterodorsally inclined (1).

Unambiguous: *Hauffiosaurus* +*Attenborosaurus* + *Peloneustes* (0->1), *Seelyosaurus* (0->1).

New character.

**146. Posteriormost dorsal rib facets:** prominent transverse process located entirely on neural arch (0); rib facet split between neural arch and centrum [‘sacralised’], but bears a typical posterior dorsal rib (1).

Unambiguous: Microcleididae (0->1), *Bobosaurus* (0->1).

New character.

**147. Sacral ribs:** cylindrical and slightly expanded towards distal end (0); transversely expanded, dorsoventrally thin and sheet-like (1).

Unambiguous: *Peloneustes* (0->1).

Ketchum & Benson (2011, character 194).

**148. Caudal vertebral count:** 33–35 (0); 36–40 (1); >40 (2).

Unambiguous: Microcleididae + *Plesiopterys* + *Cryptoclidus* (0->2), *Bobosaurus* (0->1), *Meyerasaurus* (0->1).

New character.

**149. Caudal ribs facet location in proximal–middle caudal vertebrae:** located dorsally, contacting or almost contacting neural arch (0); at midheight of centrum, neural arch does not form part of facet (1).

Unambiguous: *Seeleyosaurus* +++ (0->1), *Bobosaurus* (0->1).

New character.

**150. Caudal centra, outline of middle caudal centra in anterior view:** suboval (0); lateral surfaces converge ventrally so centrum appears to ‘taper’ ventrally (1); subrectangular, chevron facets widely spaced and located ventrolaterally, ventral surface approximately flat giving ‘square’ appearance to centrum in anterior view (2).

Unambiguous: Plesiosauroidea (2->0).

DELTRAN: *Peloneustes* (2->1).

ACCTRAN: *Attenborosaurus* + *Peloneustes* (2->1).

New character.

**151. Caudal centra, length:height ratio of proximal elements:** >0.9 (0); 0.6-0.8 (1); <0.55 (2).

Unambiguous: *Cryptoclidus* (0->1).

DELTRAN: *Macroplata* +++ (0->1), Pliosauridae (0->1), *‘R.’ megacephalus* (1->2), R. *zetlandicus* (1->2).

ACCTRAN: *Maresaurus* +++ (1->2), Plesiosauroidea (1->0), Plesiosauria (0->1), *R. thorntoni* (2->1).

New character.

**152. Middle and distal caudal vertebrae, chevron facets:** low, flush with level of ventral surface of centrum (0); prominent and project ventrally (1).

Unambiguous: *Seeleyosaurus* +++ (0->1), *Pistosaurus* [postcranium] (0->1), *Eoplesiosaurus* (0->1).

New character.

**153. Proximal caudal centra: width to height ratio:** subequal width:height, ratio = 0.9-1.1 (0); mediolaterally broad, ratio >1.1 (1).

Unambiguous: *Attenborosaurus* + *Peloneustes* (0->1).

DELTRAN: Microcleididae + *Plesiopterys* + *Cryptoclidus* (0->1).

ACCTRAN: Plesiosauroidea (0->1).

New character.

**154. Ratio of coracoid to scapular length:** > or equal to 1.8 (0); < or equal to 1.7 (1).

Unambiguous: *Peloneustes* (1->0), *Seelyosaurus* (1->0).

DELTRAN: Rhomaleosauridae + Neoplesiosauria (0->1), *Meyerasaurus* (1->0).

ACCTRAN: *Meyerasaurus* +++ (1->0), Plesiosauria (0->1).

Modified from O’Keefe (2001, character 4), Sato (2002, character 160), Druckenmiller & Russell (2008a, character 125), Smith & Dyke (2008, character 76), Ketchum & Benson (2010, character 147) (different quantification).

**155. Anteromedial margin of the coracoid:** does not contact the scapula (0); contacts the scapula (1).

Unambiguous: *Meyerasaurus* (0->1), *M. tournemirensis* (1->0).

DELTRAN: *Plesiosaurus* +++ (0->1).

ACCTRAN: Plesiosauroidea (0->1).

Sato (2002, character 169), Smith & Dyke (2008, character 79), O’Keefe & Street (2009, character 89), Ketchum & Benson (2010, character 144).

Modified from O’Keefe (2001, character 137), O’Keefe & Wahl (2003, character 77), Druckenmiller & Russell (2008a, character 122), O’Keefe & Street (2009, character 68) (coracoid-clavicle/interclavicle contact coded separately by character 156 here).

Modified from Sato (2002, character 158) (character coding presence of a posteromedial process of the scapula).

This character was scored as ‘?’ in young juveniles (e.g. *Plesiopterys*).

**156. Anteromedial margins of the coracoids:** do not contact the dermal girdle elements (0); contacts dermal girdle elements (1).

Unambiguous: *Cryptoclidus* (1->0).

DELTRAN: *Microcleidus* *homalospondylus* + *M. brachypterygius* (1->0), *Seelyosaurus* (1->0).

ACCTRAN: *Seeleyosaurus* +++ (1->0), *M. tournemirensis* (0->1).

Sato (2002, character 155), Ketchum & Benson (2010, character 143).

Modified from O’Keefe (2001, character 137), O’Keefe & Wahl (2003, character 77), Druckenmiller & Russell (2008a, character 122), O’Keefe & Street (2009, character 68) (coracoid-scapula contact coded separately by character 155 here).

**157. Scapula morphology:** dorsal blade expanding ventrally to form acetabular region, lacks expanded ventral plate (0); triradiate with expansive ventral plate (1).

DELTRAN: Rhomaleosauridae + Neoplesiosauria (0->1).

ACCTRAN: Plesiosauria (0->1).

New character.

**158. Contact of the ventral plates of the scapulae along the midline:** do not meet along the midline (0); meet along the ventral midline (1).

DELTRAN: *Cryptoclidus* (0->1), *M. homalospondylus* (0->1).

ACCTRAN: *Microcleidus* *homalospondylus* + *M. brachypterygius* (0->1), *Plesiopterys* + *Cryptoclidus* (0->1).

Bardet et al (1999, character 24), Carpenter (1999, character 19), Sato (2002, character 156), Druckenmiller & Russell (2008a, character 123), Smith & Dyke (2008, character 80), O’Keefe & Street (2009, character 66), Ketchum & Benson (2010, character 145), Vincent (2010, character 56).

Modified from O’Keefe (2001, character 135), O’Keefe & Wahl (2003, character 75).

**159. Scapula blade, outline of anterior margin in lateral view:** approximately straight, weakly concave or weakly convex (0); pronounced posterodorsal inflection (1); distinct concave region anterodorsally (2).

Unambiguous: Pliosauridae (0->1), *Eurycleidus* (0->1), *Pistosaurus* [postcranium] (0->1), *Plesiosaurus* (0->1).

DELTRAN: *M. homalospondylus* (0->1).

ACCTRAN: *Microcleidus* *homalospondylus* + *M. brachypterygius* (0->1).

Modified from O’Keefe (2008, character 37), Smith & Dyke (2008, character 81), Ketchum & Benson (2011, character 195) (addition of state 2).

**160. Shape of the anterolateral margin of the scapula where the dorsal ramus meets the ventral ramus:** flat or gently convex (0); forms prominent ridge or shelf (1); inapplicable, ventral plate not developed (?).

Unambiguous: *Cryptoclidus* (1->0).

Sato (2002, character 157), Druckenmiller & Russell (2008a, character 124), Ketchum & Benson (2010, character 146).

**161. Scapular blade, anteroposterior width:** width at distal end subequal to that at midlength (0); narrow, tapering dorsally (1); broad, distal part expanded relative to midlength (2).

Unambiguous: Microcleididae + *Plesiopterys* + *Cryptoclidus* (0->2), Neoplesiosauria (2->0), *Augustasaurus* (2->1), *Cryptoclidus* (2->1).

DELTRAN: *H. zanoni* (0->2), *M. homalospondylus* (2->0).

ACCTRAN: *Hauffiosaurus* [node] (0->2), *Microcleidus* *homalospondylus* + *M. brachypterygius* (2->0).

O’Keefe (2001, character 132), Sato (2002, character 159), Ketchum & Benson (2011, character 196).

**162. Scapula blade, medial surface:** smoothly convex or flat (0); robust buttress oriented parallel to long axis of blade (1)

DELTRAN: *Hauffiosaurus* +*Attenborosaurus* + *Peloneustes* (1->0), *Cryptoclidus* (1->0).

ACCTRAN: Pliosauridae (1->0), *Plesiopterys* + *Cryptoclidus* (1->0).

New character.

[the buttress is marked off from the blade in *Eurycleidus*, but not marked off in *R. thorntoni* and *Archaeonectrus*, in which it is just a strongly convex medial surface]

**163. Coracoid, posterolateral cornu:** does not extend as far laterally as glenoid (0); extends lateral to glenoid (1); extends to level of glenoid (2).

Unambiguous: *Seeleyosaurus* +++ (0->2), *Peloneustes* (0->2).

DELTRAN: *Cryptoclidus* (0->1), *M. brachypterygius* (2->1).

ACCTRAN: *Microcleidus* *homalospondylus* + *M. brachypterygius* (2->1), *Plesiopterys* + *Cryptoclidus* (0->1).

Ketchum & Benson (2011, character 151).

Modified from Bardet et al (1999, character 25), O’Keefe (2001, character 142), Sato (2002, character 166), Großmann (2007, character 32), Druckenmiller & Russell (2008a, character 128), Ketchum & Benson (2010, character 151), Vincent (2010, character 57) (addition of state 2).

Modified from Smith & Dyke (2008, character 75).

[report as ‘?’ in young juveniles (e.g. *Plesiopterys*)

**164. Coracoid, median fenestra/large embayment:** absent, although intercoracoid contact may be slightly split posteriorly (0); median embayment present (1); posterior processes strongly divergent forming prominent V-shaped or otherwise mediolaterally narrow emargination (2).

Unambiguous: *Attenborosaurus* (0->2), *Augustasaurus* (0->2).

DELTRAN: *Eurycleidus* +++ (0->2).

ACCTRAN: *Avalonnectes* +++ (0->2).

State 1 from Bardet et al (1999, character 27), O’Keefe (2001, character 141), Sato (2002, character 164), O’Keefe & Wahl (2003, character 79), Großmann (2007, character 33), Druckenmiller & Russell (2008a, character 127), O’Keefe & Street (2009, character 70), Ketchum & Benson (2010, character 149).

State 2 modified from Smith & Dyke (2008, character 77.

**165. Coracoid, shape of anterior process:** anteroposteriorly long and transversely broad, approximately rectangular (0); anteroposteriorly long and transversely narrow (1); anteroposteriorly short and subtriangular (2).

Unambiguous: *Hauffiosaurus* +*Attenborosaurus* + *Peloneustes* (0->2), *Microcleidus* [node] (0->1).

DELTRAN: *Cryptoclidus* (0->1).

ACCTRAN: *Plesiopterys* + *Cryptoclidus* (0->1).

Ketchum & Benson (2011, character 150).

State 1 from Ketchum & Benson (2010, character 150); modified from Smith & Dyke (2008, character 78).

Modified from Bardet et al (1999, character 26), Sato (2002, characters 161–162), Albright et al. (2007, character 25), Großmann (2007, character 31), O’Keefe (2008, character 25), O’Keefe & Street (2009, character 88) (these characters coded the relative length of the anterior process).

**166. Coracoid, posterior margin, outline in dorsal view:** oriented approximately mediolaterally, may be convex, straight or weakly concave (0); anterolaterally oriented ['posterior extension' of Albright *et al.* 2007] (1); possesses by distinct posterior process adjacent to midline (2); oriented posterolaterally (3).

Unambiguous: *Seeleyosaurus* +++ (0->3).

DELTRAN: *Eurycleidus* +++ (0->1).

ACCTRAN: *Avalonnectes* +++ (0->1).

New character.

State 1 from Albright et al. (2007, character 26), O’Keefe (2008, character 26).

**167. Coracoid, dorsoventral height of anterior process:** dorsoventrally low and thus plate-like (0); taller dorsoventrally than mediolaterally (1).

DELTRAN: *Microcleidus* [node] (0->1), *Cryptoclidus* (0->1).

ACCTRAN: *Seeleyosaurus* +++ (0->1), *Plesiopterys* + *Cryptoclidus* (0->1).

Ketchum & Benson (2011, character 197).

**168. Coracoid, robust buttress on dorsal [visceral] surface connecting glenoid to median symphysis, orientation:** posteromedially (0); mediolaterally, and forms posterior margin of an anterior depression (1); mediolaterally oriented, but located anteriorly so anterior depression is absent (2).

DELTRAN: Rhomaleosauridae + Neoplesiosauria (0->1), *Cryptoclidus* (1->2).

ACCTRAN: *Plesiopterys* + *Cryptoclidus* (1->2), Plesiosauria (0->1).

Modified from Sato (2002, character 163) (addition of state 2 from Benson [in press, character 214]).

**169. Coracoid, low, mediolaterally oriented buttress connecting glenoid to median symphysis on ventral surface:** present (0); absent (1).

Unambiguous: Microcleididae + *Plesiopterys* + *Cryptoclidus* (0->1), *Pistosaurus* [postcranium] (0->1).

DELTRAN: *H. zanoni* (0->1).

ACCTRAN: *Hauffiosaurus* [node] (0->1).

New character.

**170. Clavicle/interclavicle complex, shape of anterior margin:** concave, anteroposterior depth of concavity approximately 0.8 times the mediolateral width or greater (0); anteriorly convex or pointed (1); transversely broad and weakly concave, mediolateral width at least 4.0 times the anteroposterior depth (2); transversely narrow and weakly concave (3).

Unambiguous: *Meyerasaurus* (2->0).

DELTRAN: *Seeleyosaurus* +++ (2->3), *Attenborosaurus* (2->3), *Plesiosaurus* (2->0), *Plesiopterys* (2->3).

ACCTRAN: *Attenborosaurus* + *Peloneustes* (2->3), Microcleididae + *Plesiopterys* + *Cryptoclidus* (0->3), Plesiosauroidea (2->0), *Westphaliasaurus* (3->2).

Benson (in press, character 141).

State 1 from Sato (2002, character 153), Ketchum & Benson (2010, character 141).

**171. Median pelvic bar:** present (0); absent (1).

Unambiguous: *Attenborosaurus* + *Peloneustes* (0->1), *M. brachypterygius* (0->1).

DELTRAN: *Cryptoclidus* (0->1).

ACCTRAN: *Plesiopterys* + *Cryptoclidus* (0->1).

Carpenter (1999, character 20), O’Keefe (2001, character 146), Sato (2002, character 177), O’Keefe & Wahl (2003, character 81), Druckenmiller & Russell (2008a, character 145), O’Keefe & Street (2009, character 72), Ketchum & Benson (2010, character 171).

This was scored as ‘?’ in young juveniles (*Plesiopterys*).

The pelvic bar was considered absent where the posteromedial process of the pubis or anteromedial process of the ischium tapers to a point (eg. *Cryptoclidus*), and present where these processes for mediolaterally thick, regardless of whether they contact each other.

**172. Ilium curvature shaft in lateral view:** appears straight, pelvic articular end equally expanded anteriorly and posteriorly (0); curves anterodorsally, articular end expanded only anteriorly (1); sigmoidal (2).

Unambiguous: *Cryptoclidus* (0->1).

Sato (2002, character 170), Albright et al. (2007, character 29), O’Keefe (2008, character 29), Druckenmiller & Russell (2008a, character 142), Ketchum & Benson (2010, character 167), Vincent (2010, character 60).

**173. Ilium, rotation of dorsal blade relative to long axis of proximal articular end:** ends perpendicular to one another (0); ends rotated by approximately 45 degrees relative to one another (1).

Unambiguous: *Cryptoclidus* (1->0), *Plesiosaurus* (1->0).

DELTRAN: *Peloneustes* (1->0).

ACCTRAN: *Attenborosaurus* + *Peloneustes* (1->0).

New character.

**174. Ilium, shape of dorsal expansion:** subequal anterior and posterior expansion, occupies dorsal half of ilium (0); subequal anterior and posterior expansion confined to dorsal one-third of ilium (1); assymetrical, extends much further posterodorsally than anteriorly, dorsal surface slopes posterodorsally (2); inapplicable, dorsal expansion absent (?).

Unambiguous: *Microcleidus* [node] (0->2), *Peloneustes* (0->2).

DELTRAN: Rhomaleosauridae + Neoplesiosauria (2->0), *R. thorntoni* (0->2).

ACCTRAN: *Rhomaleosaurus* *zetlandicus* + *R. thorntoni* (0->2), Plesiosauria (2->0).

Modified from Sato (2002, character 172), Smith & Dyke (2008, character 86), Ketchum & Benson (2010, character 168), Vincent (2010, character 61).

**175. Ilium, anteroposterior width of dorsal expansion:** expanded, between 1.5–2.0 times the minimum anteroposterior width of the shaft (0); large, over 2.5 times minimum shaft width (1); slight, just greater than minimum shaft width (2); tapering, less than minimum shaft width (3).

Unambiguous: *Peloneustes* (0->1).

Modified from Ketchum & Benson (2011, character 201) (addition of state 3 from Sato [2002, character 172]).

**176. Ilium, tubercle on posterior surface around midlength:** absent (0); present as a tubercle (1); rugose crest present (2)

Unambiguous: *Cryptoclidus* (0->1), *Pistosaurus* [postcranium] (0->2), *Stratesaurus* (0->2).

Modified from Ketchum & Benson (2011, character 198) (addition of state 2).

**177. Ilium ratio of length to minimum anteroposterior width:** <3.0 (0); 4.0–5.2 (1); >5.3 (2).

Unambiguous: *Cryptoclidus* (2->1), *M. brachypterygius* (2->1), *Macroplata* (1->2), *Yunguisaurus* (0->1).

DELTRAN: *Plesiosaurus* +++ (1->2), Rhomaleosauridae + Neoplesiosauria (0->1), *H. zanoni* (1->2).

ACCTRAN: *Hauffiosaurus* [node] (1->2), Plesiosauroidea (1->2), Plesiosauria (0->1).

Modified from Smith & Dyke (2008, character 85).

**178. Ilium, pronounced, broad fossa on medial surface of the dorsal end:** present (0); absent or weak (1).

Unambiguous: Microcleididae (1->0).

DELTRAN: *Avalonnectes* +++ (1->0).

ACCTRAN: *Macroplata* +++ (1->0).

A prominent proximodistally oriented ridge forms the anterior margin of this fossa, which is present in rhomaleosaurids (e.g. *Avalonnectes*; Fig. 2D–E) and microcleidids. Where the ridge is absent, the fossa is scored as weak or absent (0).

New character.

**179. Pubis, ratio of anteroposterior length to minimum mediolateral width:** < or equal to 1.2 (0); >1.3 (1).

Unambiguous: *Peloneustes* (0->1).

Modified from Smith & Dyke (2008, character 83), Ketchum & Benson (2011, character 199), Vincent (2010, character 58).

**180. Anterolateral cornu of pubis:** absent or weak (0); present (1).

Unambiguous: *Cryptoclidus* (0->1).

Sato (2002, character 174), Druckenmiller & Russell (2008a, character 148), Ketchum & Benson (2010, character 174).

**181. Ischium, length:width ratio:** < or equal to 0.9 (0); 1.0–1.3 (1); >1.4 (2).

Unambiguous: *Microcleidus* *homalospondylus* + *M. brachypterygius* (1->0), *Macroplata* (1->2), *Peloneustes* (1->2), *Yunguisaurus* (1->0).

DELTRAN: *H. zanoni* (1->0).

ACCTRAN: *Hauffiosaurus* [node] (1->0).

Modified from Sato (2002, character 175), Albright et al. (2007, character 31), Druckenmiller & Russell (2008a, character 147), O’Keefe (2008, character 31), Smith & Dyke (2008, character 84), Ketchum & Benson (2010, character 173) (different quantification).

**182. Limbs, postaxial accessory ossicles:** absent (0); present (1).

Unambiguous: *Attenborosaurus* + *Peloneustes* (1->0), *Archaeonectrus* (1->0), *Plesiosaurus* (1->0).

DELTRAN: Rhomaleosauridae + Neoplesiosauria (0->1).

ACCTRAN: Plesiosauria (0->1).

Modified from Druckenmiller & Russell (2008a, character 137), Ketchum & Benson (2010, characters 160–161) (merged separate characters/states of the propodial and epipodial rows).

Modified from O’Keefe (2001, character 153), O’Keefe & Wahl (2003, character 87), Albright et al. (2007, character 20), O’Keefe (2008, character 20), O’Keefe & Street (2009, character 78) (split single character describing all ‘supernumary ossifications’ into separate characters for postaxial and preaxial [ch. 183] ossifications).

Sato (2002, characters 199–200) (merged separate characters for forelimb and hindlimb).

This character was scored as ‘?’ in young juveniles.

**183. Limbs, preaxial accessory ossicles:** absent (0); present (1).

DELTRAN: *Cryptoclidus* (0->1).

ACCTRAN: *Plesiopterys* + *Cryptoclidus* (0->1).

Modified from O’Keefe (2001, character 153), O’Keefe & Wahl (2003, character 87), Albright et al. (2007, character 20), O’Keefe (2008, character 20), O’Keefe & Street (2009, character 78) (split single character describing all ‘supernumary ossifications’ into separate characters for postaxial [ch. 182] and preaxial ossifications).

This character was scored as ‘?’ in young juveniles.

**184. Propodials, dorsal and ventral surfaces of distal half:** uniformly convex or flat with robust pre-and postaxial margins (0); weakly concave region separates central, convex portion from strongly tapering, flange-like pre- and postaxial margins (1).

Unambiguous: *Peloneustes* (0->1).

New character.

**185. Humerus, long axis curvature in anterior view:** straight or almost straight (0); pronounced dorsodistal curve (1).

Unambiguous: Neoplesiosauria (1->0), *Cryptoclidus* (0->1).

New character.

**186. Femur, long axis curvature in anterior view:** straight or almost straight (0); pronounced dorsodistal curve (1); pronounced ventrodistal curve (2).

Unambiguous: *Cryptoclidus* (0->1), *Westphaliasaurus* (0->1).

DELTRAN: *R. thorntoni* (0->1), R. *zetlandicus* (0->2).

ACCTRAN: *Meyerasaurus* +++ (0->1), R. *zetlandicus* (1->2).

New character.

**187. Humerus length versus width ratio:** >2.9 (0); 1.9–2.3 (1); 2.4–2.7 (2); < 1.6 (3).

Unambiguous: *Hauffiosaurus* [node] (1->2), *Cryptoclidus* (1->3), *Eoplesiosaurus* (1->2).

DELTRAN: Rhomaleosauridae + Neoplesiosauria (0->1).

ACCTRAN: Plesiosauria (0->1).

Modified from Bardet et al (1999, character 29), Sato (2002, character 184), Druckenmiller & Russell (2008a, character 132), Smith & Dyke (2008, character 87), Ketchum & Benson (2010, character 154) (different quantification).

**188. Preaxial margin of distal humerus in dorsal or ventral view:** straight or convex (0); concave [distal humerus expands anteriorly], but anterior expansion relatively small, substantially less than posterior expansion (1); concave, and anterior expansion is large, approaching the size of the posterior expansion (2).

Unambiguous: *Rhomaleosaurus* [node] (0->1), *Microcleidus* *homalospondylus* + *M. brachypterygius* (0->1), *Cryptoclidus* (0->2), *Eurycleidus* (0->1), *Peloneustes* (0->1).

DELTRAN: *Eretmosaurus* (0->1), *Westphaliasaurus* (0->1).

ACCTRAN: *Seeleyosaurus* +++ (1->0), Microcleididae (0->1).

Modified from O’Keefe (2001, character 150), Sato (2002, character 186), O’Keefe & Wahl (2003, character 82), Druckenmiller & Russell (2008a, character 133), O’Keefe & Street (2009, character 73), Ketchum & Benson (2010, character 157), Vincent (2010, character 62) (addition of state 2).

Modified from Smith & Dyke (2008, character 89).

**189. Humerus, sharp longitudinal ridge on anterior surface:** absent (0); present (1).

DELTRAN: *Eurycleidus* (0->1), *Plesiosaurus* (0->1), *‘R.’ megacephalus* (0->1), *Eoplesiosaurus* (0->1).

ACCTRAN: *Archaeonectrus* +++ (1->0), Pliosauridae (1->0), Microcleididae + *Plesiopterys* + *Cryptoclidus* (1->0), Plesiosauria (0->1).

Smith & Dyke (2008, character 90).

**190. Humerus, shape of the distal end:** uniformly convex (0); humerus distinctly angled for articulation with the epipodials (1).

Unambiguous: Microcleididae + *Plesiopterys* + *Cryptoclidus* (0->1), *Westphaliasaurus* (1->0).

O’Keefe (2001, character 152), O’Keefe & Wahl (2003, character 83), Druckenmiller & Russell (2008a, character 136), Smith & Dyke (2008, character 91), O’Keefe & Street (2009, character 74), Ketchum & Benson (2010, character 159).

**191. Humerus, inclination of proximal end in dorsal view:** not inclined, extends proximally so shaft appears straight (0); inclined anteriorly so shaft appears sigmoidal (1); inclined posteriorly so that the proximal portion of the anterior margin is convex in dorsal view [low bulge often located proximally on anterior surface] (2).

Unambiguous: *Hauffiosaurus* +*Attenborosaurus* + *Peloneustes* (2->0), *Plesiopterys* + *Cryptoclidus* (2->0), *R. thorntoni* (0->2), *Westphaliasaurus* (2->0).

DELTRAN: *‘R.’ megacephalus* +++ (2->0), *Eurycleidus* (2->0).

ACCTRAN: *Eurycleidus* +++ (2->0), *Meyerasaurus* (0->2).

State 1 from O’Keefe & Wahl (2003, character 95), Albright et al. (2007, character 18), O’Keefe (2008, character 18), O’Keefe & Street (2009, character 86).

State 2 from Smith & Dyke (2008, character 89).

**192. Humerus, shallow groove on ventral surface between epipodial facets** (flexor groove)**:** present and prominent (0); absent (1); present but anteroposteriorly short and shallow (2).

Unambiguous: *Meyerasaurus* +++ (0->1), *Hauffiosaurus* +*Attenborosaurus* + *Peloneustes* (0->1), *Westphaliasaurus* (0->2).

DELTRAN: *Cryptoclidus* (0->1).

ACCTRAN: *Plesiopterys* + *Cryptoclidus* (0->1).

New character.

Storrs (1999) described this groove as an autapomorphy of *Plesiosaurus dolichodeirus*. However, it is present in basal sauropterygians and several basal plesiosaurians.

**193. Humerus, orientation of the tuberosity:** not tilted, tuberosity rises dorsally (0); posterodorsally tilted (1); inapplicable, trochanters absent (?).

DELTRAN: *Cryptoclidus* (0->1).

ACCTRAN: *Plesiopterys* + *Cryptoclidus* (0->1).

Sato (2002, character 192).

**194. Radius, ratio of length to maximum width:** >2.7 (0); 1.1–1.5 (1); < or equal to 1.0 (2).

Unambiguous: *Peloneustes* (1->2).

DELTRAN: Rhomaleosauridae + Neoplesiosauria (0->1).

ACCTRAN: Plesiosauria (0->1).

Modified from Smith & Dyke (2008, character 93), Ketchum & Benson (2010, character 162) (radius).

Modified from Sato (2002, character 202), Druckenmiller & Russell (2008a, character 138), Vincent (2010, character 64) (radius and ulna).

Modified from O’Keefe (2001, character 161), O’Keefe & Wahl (2003, character 86), Albright et al. (2007, character 21), O’Keefe (2008, character 21), O’Keefe & Street (2009, character 77) (epipodial proportions).

**195.Tibia, ratio of length to maximum width:** >2.5 (0); 1.1–1.5 (1); < or equal to 1.0 (2).

Unambiguous: *Cryptoclidus* (1->2).

DELTRAN: Rhomaleosauridae + Neoplesiosauria (0->1), *Peloneustes* (1->2).

ACCTRAN: *Attenborosaurus* + *Peloneustes* (1->2), Plesiosauria (0->1).

Modified from Ketchum & Benson (2010, character 177) (tibia).

Modified from Sato (2002, character 203), Druckenmiller & Russell (2008a, character 151), Vincent (2010, character 66) (tibia and fibula).

Modified from O’Keefe (2001, character 161), O’Keefe & Wahl (2003, character 86), Albright et al. (2007, character 21), O’Keefe (2008, character 21), O’Keefe & Street (2009, character 77) (epipodial proportions).

**196. Radius, morphology:** preaxial margin concave (0); straight or convex (1).

Unambiguous: *Augustasaurus* (0->1), *Peloneustes* (0->1).

Ketchum & Benson (2010, character 163).

Modified from O’Keefe & Street (2009, character 90) (radius).

Modified from Sato (2002, character 204), Druckenmiller & Russell (2008a, character 139), Vincent (2010, character 65) (radius and ulna).

**197. Radius, prominent anterior flange extends from anteroproximal surface:** absent (0); present (1).

Unambiguous: *Hauffiosaurus* [node] (0->1), Microcleididae (0->1).

DELTRAN: *Meyerasaurus* +++ (0->1).

ACCTRAN: Rhomaleosauridae (0->1).

New character.

**198. Ulna, morphology:** postaxial margin concave (0); convex (1).

Unambiguous: *M. brachypterygius* (1->0).

DELTRAN: Rhomaleosauridae + Neoplesiosauria (0->1).

ACCTRAN: Plesiosauria (0->1).

O’Keefe (2001, character 158), O’Keefe & Wahl (2003, character 85), O’Keefe & Street (2009, character 76), Ketchum & Benson (2010, character 164) (ulna).

Modified from Smith & Dyke (2008, character 92) (ulna and fibula).

**199. Tibia, morphology:** preaxial side of tibia concave (0); convex or straight (1).

Ketchum & Benson (2010, character 178) (tibia).

Unambiguous: *Cryptoclidus* (0->1).

DELTRAN: *H. tomistomimus* (0->1), *Peloneustes* (0->1).

ACCTRAN: *Hauffiosaurus* +*Attenborosaurus* + *Peloneustes* (0->1), *H. zanoni* (1->0).

Modified from Sato (2002, character 205), Druckenmiller & Russell (2008a, character 152), Vincent (2010, character 67) (tibia and fibula).

**200. Fibula, morphology:** postaxial side of fibula concave (0); convex (1).

DELTRAN: Rhomaleosauridae + Neoplesiosauria (0->1).

ACCTRAN: Plesiosauria (0->1).

Modified from Sato (2002, character 205), Druckenmiller & Russell (2008a, character 152), Vincent (2010, character 67) (tibia and fibula).

Modified from Smith & Dyke (2008, character 92) (ulna and fibula).

**201. Epipodial foramen** [spatium interossium; antebrachial foramen]**:** present, proximodistal length slightly shorter than epipodials (0); present but short, proximodistal length less than half epipodial length (1); absent (2).

Unambiguous: *Cryptoclidus* (0->2).

Modified from Sato (2002, characters 208–209), Albright et al. (2007, character 23), O’Keefe (2008, character 23).

**202. Forelimb epipodials, ratio of maximum radius length to maximum ulna length:** 1.0–1.3 (0); 1.4–1.7 (1); > or equal to 2.0 (2).

Unambiguous: *Cryptoclidus* (0->2), *Peloneustes* (0->1).

New character.

Modified from Sato (2002, character 202

**203. Radius, posterodistal facet for intermedium:** absent (0); present (1).

Unambiguous: *Plesiopterys* (0->1).

DELTRAN: *Microcleidus* *homalospondylus* + *M. brachypterygius* (0->1), *H. tomistomimus* (0->1), *Peloneustes* (0->1), R. *zetlandicus* (0->1), *Seelyosaurus* (0->1).

ACCTRAN: *Rhomaleosaurus* [node] (0->1), *Hauffiosaurus* +*Attenborosaurus* + *Peloneustes* (0->1), *Seeleyosaurus* +++ (0->1), *H. zanoni* (1->0), *M. tournemirensis* (1->0).

New character.

**204. Tibia, posterodistal facet for intermedium:** absent (0); present (1).

Unambiguous: *Hauffiosaurus* +*Attenborosaurus* + *Peloneustes* (0->1).

DELTRAN: *Archaeonectrus* +++ (0->1), *Seeleyosaurus* +++ (0->1), *Meyerasaurus* (0->1), *Plesiopterys* (0->1), *Westphaliasaurus* (0->1).

ACCTRAN: *Eurycleidus* +++ (0->1), Microcleididae + *Plesiopterys* + *Cryptoclidus* (0->1), *Cryptoclidus* (1->0), *Eretmosaurus* (1->0), *‘R.’ megacephalus* (1->0).

Ketchum & Benson (2011, character 200).

**205. Limbs, position of the fifth metapodial:** lies in the metapodial row (0); shifted proximally so that the proximal half is in the distal mesopodial row (1); shifted proximally so the entire fifth metapodial is in the mesopodial row (2).

Unambiguous: *Cryptoclidus* (1->2).

DELTRAN: Rhomaleosauridae + Neoplesiosauria (0->1).

ACCTRAN: Plesiosauria (0->1).

Modified from O’Keefe (2001, character 163), Druckenmiller & Russell (2008a, character 140), Ketchum & Benson (2010, character 165) (addition of state 2 from Sato (2002, characters 211–212).

**206. Metapodials III and IV, morphology of proximal ends:** straight, anteroposteriorly oriented butt contact with distal tarsals (0); angled facets, proximal articular surface of metapodial IV bi-faceted (1).

Unambiguous: *Plesiopterys* + *Cryptoclidus* (0->1), *Peloneustes* (0->1), *Stratesaurus* (0->1).

New character.

**207. Phalanx proportions:** long and slender [~2-3 times as long proximodistally as broad anteroposteriorly] (0); short and robust (1).

Unambiguous: *Attenborosaurus* + *Peloneustes* (0->1).

DELTRAN: *Seeleyosaurus* +++ (0->1), *Cryptoclidus* (0->1), *Westphaliasaurus* (0->1).

ACCTRAN: Microcleididae + *Plesiopterys* + *Cryptoclidus* (0->1), *Eretmosaurus* (1->0), *Plesiopterys* (1->0)

New character.
