# Supplementary material for: High Diversity, Low Disparity and Small Body Size in Plesiosaurs (Reptilia, Sauropterygia) from the Triassic–Jurassic Boundary
Source: PLoS One. 2012 Mar 16;7(3):e31838. doi: 10.1371/journal.pone.0031838 (PMC3306369; doi:10.1371/journal.pone.0031838)
Supplement: Appendix S3 — Phylogenetic data matrix. [PDF] [file pone.0031838.s003.pdf]

	1	2	3	4	5	6	7	8	9	10	11	12	13	14	15	16
Yunguisaurus	0	0	0	0	0	0	?	?	0	0	0	0	0	0	0	0
***Pistosaurus* [postcranium]**	?	?	?	?	?	?	?	?	?	?	?	?	?	?	?	?
***Pistosaurus* [skull]**	0	1	0	1	0	?	0	0	0	0	0	0	0	0	0	0
Augustasaurus	0	1	0	1	0	?	0	0	0	0	?	0	0	0	0	0
Bobosaurus	?	?	?	?	?	?	?	?	?	?	?	?	?	?	?	0
**NHMUK_49202**	1	1	1	0	0	?	0	1	2	0	?	0	0	0	2	1
Stratesaurus	0	0	1	0	0	0	0	1	1	0	1	0	0	1	0	0
Macroplata	1	1	0	1	0	0	?	?	0	0	?	1	0	1	0	1
Avalonnectes	?	?	1	?	?	0	?	?	?	0	1	?	?	1	0	?
Eurycleidus	?	?	?	?	?	?	?	?	?	?	?	?	?	?	?	?
Meyerasaurus	1	0	1	0	?	0	?	2	0	?	?	0	2	?	?	1
Maresaurus	1	1	0	0	0	?	1	2	2	0	?	1	2	?	?	1
'R.' megacephalus	1	0	0	0	1	1	1	2	2	0	0	1	2	1	0	1
Archaeonectrus	1	0	0	1	0	1	?	?	?	0	?	1	0	2	?	1
R. cramptoni	1	0	0	0	0	1	1	2	2	0	?	1	0	2	0	1
R. zetlandicus	1	1	0	0	?	1	1	2	2	0	?	1	0	2	0	1
R. thorntoni	1	1	?	?	?	?	?	2	?	0	?	1	0	?	?	1
Thalassiodracon	0	0	1	0	0	0	1	1	0	0	1	0	0	1	1	1
H. longirostris	0	0	0	1	?	0	0	?	0	?	?	1	2	1	1	1
H. tomistomimus	0	0	0	1	?	0	0	2	0	0	?	1	?	1	1	0
H. zanoni	0	0	?	1	?	0	?	?	0	?	?	?	?	?	?	?
Attenborosaurus	0	0	0	1	0	0	0	?	?	?	1	1	0	?	?	1
Peloneustes	1	0	0	1	1	1	0	2	0	1	0	0	0	2	1	0
Eoplesiosaurus	?	?	?	?	?	?	?	?	?	?	?	?	?	?	?	?
Plesiosaurus	0	0	1	0	0	?	0	1	0	0	?	0	0	1	0	0
Eretmosaurus	?	?	?	?	?	?	?	?	?	?	?	?	?	?	?	?
Westphaliasaurus	?	?	?	?	?	?	?	?	?	?	?	?	?	?	?	?
Seelyosaurus	0	0	1	0	?	0	0	?	0	0	?	0	2	?	?	?
M.tournemirensis	0	0	0	0	0	0	?	1	2	0	1	0	2	1	0	0
M. brachypterygius	0	0	0	0	0	0	?	1	0	0	1	0	2	1	0	0
M. homalospondylus	0	0	0	0	0	0	0	1	0	0	1	0	2	1	0	?
Plesiopterys	0	0	1	0	?	0	?	1	?	?	?	0	0	?	?	?
Cryptoclidus	0	0	1	0	?	0	0	1	1	?	1	0	?	1	0	0

	17	18	19	20	21	22	23	24	25	26	27	28	29	30	31	32
Yunguisaurus	?	?	0	0	1	0	0	?	0	0	1	1	0	0	1	?
***Pistosaurus* [postcranium]**	?	?	?	?	?	?	?	?	?	?	?	?	?	?	?	?
***Pistosaurus* [skull]**	?	?	0	0	1	0	0	0	0	0	1	?	1	0	0	0
Augustasaurus	?	?	0	0	1	0	0	0	0	0	1	0	0	0	0	0
Bobosaurus	?	?	?	?	?	?	?	?	?	?	?	?	?	?	?	?
**NHMUK_49202**	0	0	0	0	1	0	?	0	1	0	1	0	1	0	0	0
Stratesaurus	?	?	0	1	1	0	0	0	0	1	0	0	?	0	?	0
Macroplata	1	1	0	{02}	?	0	0	?	1	1	1	0	1	0	?	?
Avalonnectes	?	?	?	?	?	?	0	?	?	?	0	0	1	?	?	0
Eurycleidus	?	?	?	?	?	?	?	?	?	?	?	?	?	?	?	?
Meyerasaurus	1	?	?	0	1	0	?	0	0	?	?	?	?	0	?	?
Maresaurus	1	2	0	{02}	1	0	0	0	?	?	?	?	?	?	?	?
'R.' megacephalus	0	0	0	2	1	0	0	0	1	?	1	0	0	0	?	0
Archaeonectrus	1	1	1	?	?	0	?	?	?	?	0	?	?	0	?	0
R. cramptoni	0	0	1	2	1	1	0	0	0	1	0	?	1	0	0	0
R. zetlandicus	0	0	1	2	1	1	?	0	0	1	0	?	?	0	0	?
R. thorntoni	0	2	1	?	1	1	0	0	0	?	?	?	?	0	?	0
Thalassiodracon	1	2	0	?	?	0	0	0	0	?	0	0	0	0	0	0
H. longirostris	1	2	0	?	?	0	?	?	?	2	?	?	?	?	?	?
H. tomistomimus	?	?	0	1	0	0	2	0	2	1	0	0	0	1	0	?
H. zanoni	?	?	?	?	?	?	2	0	?	?	?	?	?	?	?	?
Attenborosaurus	1	2	?	?	?	0	0	?	0	?	?	?	?	0	?	0
Peloneustes	?	?	0	1	0	0	2	0	0	1	0	?	0	1	0	0
Eoplesiosaurus	?	?	?	?	?	?	?	?	?	?	?	?	?	?	?	?
Plesiosaurus	?	?	0	0	1	0	0	?	0	0	0	1	0	0	1	0
Eretmosaurus	?	?	?	?	?	?	?	?	?	?	?	?	?	?	?	?
Westphaliasaurus	?	?	?	?	?	?	?	?	?	?	?	?	?	?	?	?
Seelyosaurus	?	?	0	?	?	?	?	?	0	?	?	?	?	0	?	0
M.tournemirensis	?	?	0	0	1	0	0	0	0	0	0	0	0	0	1	1
M. brachypterygius	?	?	0	0	1	0	0	0	0	0	0	0	1	0	1	1
M. homalospondylus	?	?	0	0	1	0	1	?	0	?	0	0	1	0	?	1
Plesiopterys	?	?	?	?	?	0	?	?	0	?	0	1	?	0	?	?
Cryptoclidus	?	?	0	0	1	0	0	1	0	0	0	1	0	0	0	0

	33	34	35	36	37	38	39	40	41	42	43	44	45	46	47	48
Yunguisaurus	0	0	0	1	?	?	1	0	0	?	?	?	?	?	0	1
***Pistosaurus* [postcranium]**	?	?	?	?	?	?	?	?	?	?	?	?	?	?	?	?
***Pistosaurus* [skull]**	0	0	0	0	0	1	1	0	0	?	?	?	?	0	0	1
Augustasaurus	0	0	?	0	0	1	1	1	0	0	1	0	?	?	?	?
Bobosaurus	?	?	?	?	?	?	?	?	?	?	?	?	?	?	?	?
**NHMUK_49202**	0	?	0	0	1	1	1	0	0	?	1	0	?	0	1	1
Stratesaurus	0	0	0	0	0	1	0	0	0	0	1	0	1	0	1	1
Macroplata	?	?	?	0	?	?	?	?	0	?	?	?	?	?	0	1
Avalonnectes	0	0	0	0	0	1	0	0	0	0	1	0	1	0	?	?
Eurycleidus	?	?	?	?	?	?	?	?	?	?	?	?	?	?	?	?
Meyerasaurus	?	?	?	?	?	?	?	?	?	?	?	0	1	0	1	?
Maresaurus	?	?	?	0	1	1	1	0	?	?	1	0	?	0	1	1
'R.' megacephalus	0	0	0	0	0	1	1	0	0	1	1	0	1	0	1	?
Archaeonectrus	0	?	0	0	0	1	1	0	1	?	1	?	?	0	1	?
R. cramptoni	0	0	0	0	0	1	1	0	1	1	1	1	1	0	?	1
R. zetlandicus	?	?	?	0	0	1	?	0	1	?	1	1	1	0	1	1
R. thorntoni	0	0	0	?	?	?	?	?	?	1	?	?	?	?	?	?
Thalassiodracon	0	?	2	0	0	0	0	0	1	0	1	0	1	1	0	1
H. longirostris	?	?	?	?	?	0	1	?	?	?	?	?	?	?	0	1
H. tomistomimus	?	?	?	0	0	0	0	0	1	?	1	0	1	?	0	1
H. zanoni	0	?	?	?	?	?	?	?	?	0	1	?	?	?	0	1
Attenborosaurus	0	0	2	?	?	?	1	?	?	0	?	?	?	1	1	?
Peloneustes	0	0	2	0	0	0	2	1	2	0	1	0	?	0	0	1
Eoplesiosaurus	?	?	?	?	?	?	?	?	?	?	?	?	?	?	?	?
Plesiosaurus	0	0	?	0	0	0	0	0	0	0	1	0	1	0	1	1
Eretmosaurus	?	?	?	?	?	?	?	?	?	?	?	?	?	?	?	?
Westphaliasaurus	?	?	?	?	?	?	?	?	?	?	?	?	?	?	?	?
Seelyosaurus	0	1	1	?	1	?	0	?	0	?	0	?	?	1	1	?
M.tournemirensis	1	1	1	0	0	0	1	0	0	1	1	0	0	1	?	?
M. brachypterygius	1	1	1	0	1	0	1	0	0	1	0	0	?	?	?	?
M. homalospondylus	1	1	1	0	1	0	1	0	0	1	?	?	?	1	1	1
Plesiospterys	?	?	?	1	0	0	0	0	0	?	0	0	?	0	?	1
Cryptoclidus	2	0	2	1	1	0	0	1	0	0	0	0	0	0	1	0

	49	50	51	52	53	54	55	56	57	58	59	60	61	62	63	64
Yunguisaurus	2	0	?	?	?	?	?	?	?	?	?	?	?	0	2	?
***Pistosaurus* [postcranium]**	?	?	?	?	?	?	?	?	?	?	?	?	?	?	?	?
***Pistosaurus* [skull]**	?	0	?	?	?	?	?	?	?	?	?	?	?	0	2	?
Augustasaurus	?	0	?	?	0	?	0	0	0	?	?	?	?	0	2	?
Bobosaurus	?	?	?	?	?	?	?	?	?	?	?	?	?	?	?	?
**NHMUK_49202**	2	0	0	1	1	1	1	1	1	0	0	1	0	0	0	2
Stratesaurus	1	1	2	0	0	2	0	0	0	0	?	0	0	0	2	2
Macroplata	1	2	?	0	1	2	?	1	0	?	0	1	?	0	0	2
Avalonnectes	?	?	?	?	?	?	?	?	?	?	?	?	?	?	?	?
Eurycleidus	?	?	?	?	?	?	?	?	?	?	?	?	?	?	?	?
Meyerasaurus	{01}	1	?	?	?	?	?	?	?	?	?	?	?	0	2	2
Maresaurus	?	2	?	?	?	?	0	?	?	?	?	?	?	0	?	?
'R.' megacephalus	?	{12}	2	0	1	?	0	0	0	?	0	?	?	0	0	2
Archaeonectrus	?	?	?	?	?	?	?	?	?	?	?	?	?	?	?	?
R. cramptoni	?	?	?	?	1	?	0	0	0	?	?	?	?	0	2	?
R. zetlandicus	?	?	?	?	1	?	0	?	0	?	?	?	?	?	2	2
R. thorntoni	?	?	?	?	?	?	?	?	?	?	?	?	?	?	?	?
Thalassiodracon	1	1	0	0	0	2	0	0	0	1	0	0	0	0	?	0
H. longirostris	0	2	?	0	?	?	?	1	?	?	?	?	?	1	?	0
H. tomistomimus	1	2	?	?	?	?	?	1	0	?	?	?	?	1	?	0
H. zanoni	?	?	?	?	?	?	?	?	?	?	?	?	?	1	?	0
Attenborosaurus	?	?	?	?	?	?	?	?	?	?	?	?	?	?	2	?
Peloneustes	1	1	0	0	1	0	0	0	0	1	?	1	0	0	2	2
Eoplesiosaurus	?	?	?	?	?	?	?	?	?	?	?	?	?	?	?	?
Plesiosaurus	{01}	1	0	?	0	2	0	0	0	1	1	1	?	0	?	0
Eretmosaurus	?	?	?	?	?	?	?	?	?	?	?	?	?	?	?	?
Westphaliasaurus	?	?	?	?	?	?	?	?	?	?	?	?	?	?	?	?
Seelyosaurus	?	?	?	?	?	?	?	?	?	?	?	?	?	?	?	?
M.tournemirensis	2	2	?	?	?	?	0	0	0	?	?	?	0	1	2	?
M. brachypterygius	1	{12}	?	?	?	?	?	?	?	?	?	?	?	0	1	?
M. homalospondylus	1	2	0	1	0	2	0	0	0	?	?	0	0	0	2	1
Plesiospterys	1	0	1	1	0	2	0	0	0	0	0	1	0	0	1	1
Cryptoclidus	1	0	1	1	0	0	1	0	1	1	1	0	1	0	1	1

	65	66	67	68	69	70	71	72	73	74	75	76	77	78	79	80
Yunguisaurus	0	?	?	?	?	?	?	?	0	?	0	0	1	0	0	2
***Pistosaurus* [postcranium]**	?	?	?	?	?	?	?	?	?	?	?	?	?	?	?	?
***Pistosaurus* [skull]**	0	0	0	0	0	?	?	0	0	?	0	0	1	0	0	2
Augustasaurus	0	0	0	?	0	1	1	0	0	?	0	0	1	0	0	0
Bobosaurus	?	?	?	?	?	?	?	?	?	?	?	?	?	?	?	?
**NHMUK_49202**	1	?	1	0	0	0	?	0	1	1	?	0	0	?	0	0
Stratesaurus	1	?	?	?	0	0	1	0	1	0	1	1	0	?	0	0
Macroplata	1	?	?	?	?	?	?	?	?	?	1	0	0	?	?	0
Avalonnectes	?	?	?	?	?	?	?	?	?	?	?	?	?	?	?	?
Eurycleidus	?	?	?	?	?	?	?	?	?	?	?	?	?	?	?	?
Meyerasaurus	0	?	1	?	0	1	0	1	1	1	1	2	3	?	1	1
Maresaurus	1	?	0	0	0	1	0	1	0	?	1	2	3	?	1	0
'R.' megacephalus	1	1	0	0	0	1	0	0	1	0	1	1	?	?	?	0
Archaeonectrus	?	?	?	?	?	?	?	?	?	?	?	?	?	?	?	?
R. cramptoni	1	1	0	1	0	1	0	1	1	0	1	2	3	?	1	0
R. zetlandicus	1	?	?	0	?	1	?	?	1	?	1	?	3	?	?	0
R. thorntoni	?	1	?	?	0	1	?	?	?	?	?	?	?	?	?	?
Thalassiodracon	1	?	0	0	0	1	1	0	?	?	1	0	0	?	0	0
H. longirostris	?	?	?	?	?	?	?	?	?	?	1	1	0	?	0	1
H. tomistomimus	1	?	0	?	0	1	0	0	0	?	1	0	0	?	0	1
H. zanoni	1	0	1	?	0	1	0	0	0	?	1	0	0	?	0	1
Attenborosaurus	?	?	?	?	?	?	?	?	?	?	?	?	?	?	?	?
Peloneustes	1	1	0	0	1	1	0	{01}	0	?	1	2	1	1	0	2
Eoplesiosaurus	?	?	?	?	?	?	?	?	?	?	?	?	?	?	?	?
Plesiosaurus	1	0	0	1	0	1	0	0	1	0	1	0	0	?	0	0
Eretmosaurus	?	?	?	?	?	?	?	?	?	?	?	?	?	?	?	?
Westphaliasaurus	?	?	?	?	?	?	?	?	?	?	?	?	?	?	?	?
Seelyosaurus	?	?	?	?	?	?	?	?	?	?	?	?	?	?	?	?
M.tournemirensis	1	0	0	0	0	?	?	0	0	?	1	2	1	0	0	1
M. brachypterygius	1	0	0	1	0	0	1	0	0	?	1	2	1	0	0	0
M. homalospondylus	1	?	0	0	?	?	?	?	0	?	?	2	1	0	0	1
Plesiospterys	?	?	?	1	?	?	?	?	1	1	?	0	0	?	?	0
Cryptoclidus	?	0	0	1	0	1	1	0	1	1	?	0	0	?	0	?

	81	82	83	84	85	86	87	88	89	90	91	92	93	94	95	96
Yunguisaurus	?	?	?	0	?	1	0	0	0	?	1	1	0	0	1	?
***Pistosaurus* [postcranium]**	?	?	?	?	?	?	?	?	?	?	?	?	?	?	?	?
***Pistosaurus* [skull]**	0	?	?	2	0	?	?	?	?	?	?	?	?	?	?	?
Augustasaurus	0	?	?	0	0	1	0	0	0	?	1	1	0	0	1	?
Bobosaurus	?	?	?	?	?	?	?	?	?	?	?	?	?	?	?	?
**NHMUK_49202**	0	1	1	?	1	1	0	1	0	?	1	1	0	1	0	?
Stratesaurus	0	0	0	2	?	1	0	1	?	?	1	1	1	1	0	0
Macroplata	?	?	?	?	0	1	0	1	1	?	?	2	1	?	?	?
Avalonnectes	?	?	?	?	2	?	?	?	?	?	?	?	?	?	?	?
Eurycleidus	?	?	?	?	?	?	0	1	1	?	?	?	?	?	?	?
Meyerasaurus	1	0	1	1	?	0	0	1	1	?	0	2	1	1	0	2
Maresaurus	1	?	?	2	1	0	0	1	1	?	0	2	1	0	0	?
'R.' megacephalus	1	0	0	2	0	0	0	1	1	?	?	2	0	?	0	?
Archaeonectrus	?	?	?	?	?	?	0	1	?	?	1	?	1	?	?	?
R. cramptoni	1	0	0	2	?	0	?	1	1	?	0	2	1	0	0	?
R. zetlandicus	?	0	?	2	0	0	0	1	1	1	0	2	1	0	0	2
R. thorntoni	1	?	?	2	?	0	0	1	1	1	?	1	1	?	?	?
Thalassiodracon	1	?	?	0	1	1	0	1	0	1	1	1	0	0	0	0
H. longirostris	?	?	?	?	?	1	1	0	1	1	1	1	1	0	0	?
H. tomistomimus	1	?	?	?	?	1	1	0	0	1	1	1	1	0	0	?
H. zanoni	1	?	?	2	?	1	1	0	?	1	1	1	1	0	0	?
Attenborosaurus	?	?	?	?	?	0	0	1	0	?	?	?	0	?	?	?
Peloneustes	1	?	?	2	?	1	1	1	1	1	1	0	0	0	0	1
Eoplesiosaurus	?	?	?	?	?	?	?	?	?	?	?	?	?	?	?	?
Plesiosaurus	1	1	1	0	2	0	0	0	0	?	1	1	0	0	0	?
Eretmosaurus	?	?	?	?	?	?	?	?	?	?	?	?	?	?	?	?
Westphaliasaurus	?	?	?	?	?	?	?	?	?	?	?	?	?	?	?	?
Seelyosaurus	?	?	?	?	?	?	0	0	?	?	1	1	0	1	0	?
M.tournemirensis	?	?	?	2	?	?	?	?	?	?	?	?	?	?	?	?
M. brachypterygius	0	?	?	2	?	?	?	?	?	?	?	?	?	?	?	?
M. homalospondylus	0	?	?	2	2	0	0	0	0	?	?	?	0	?	?	?
Plesiospterys	0	1	0	?	1	?	2	0	?	?	1	1	0	0	0	?
Cryptoclidus	0	?	0	0	?	0	2	0	0	2	1	1	0	0	0	?

	97	98	99	100	101	102	103	104	105	106	107	108	109	110	111	112
Yunguisaurus	1	?	?	0	0	1	3	?	?	?	?	?	0	?	?	?
***Pistosaurus* [postcranium]**	?	?	?	?	?	?	?	?	?	?	?	?	?	?	?	?
***Pistosaurus* [skull]**	?	?	?	?	?	?	{12}	0	1	?	?	0	?	?	?	?
Augustasaurus	?	0	?	?	?	0	1	0	1	0	2	1	0	?	0	0
Bobosaurus	?	?	?	?	?	?	?	?	?	0	2	?	?	0	0	0
**NHMUK_49202**	1	0	0	?	?	0	0	0	1	1	1	0	0	0	0	0
Stratesaurus	1	?	0	0	0	0	1	0	0	1	1	0	0	0	0	0
Macroplata	1	0	?	?	?	0	2	0	1	1	1	1	0	0	0	0
Avalonnectes	?	?	?	?	0	?	?	?	?	?	?	?	?	?	?	?
Eurycleidus	1	?	?	?	?	?	?	?	?	1	1	1	?	?	?	?
Meyerasaurus	1	0	?	0	0	0	1	?	1	1	0	1	?	?	?	0
Maresaurus	1	0	?	0	0	0	1	?	1	1	1	1	0	?	?	0
'R.' megacephalus	1	0	?	?	?	?	?	0	0	1	0	2	?	?	?	0
Archaeonectrus	?	?	?	?	?	?	?	?	?	1	1	2	?	?	?	?
R. cramptoni	1	0	?	0	0	0	1	?	1	1	0	2	?	0	?	?
R. zetlandicus	1	0	0	0	0	0	?	?	1	1	0	2	?	?	?	?
R. thorntoni	1	0	0	0	0	?	1	1	1	1	0	1	0	?	?	?
Thalassiodracon	?	0	2	0	0	?	0	0	1	1	1	1	0	0	0	0
H. longirostris	1	0	?	?	?	2	3	0	1	1	1	2	0	?	?	?
H. tomistomimus	1	0	2	?	?	2	3	0	1	1	1	2	?	1	0	?
H. zanoni	?	0	2	?	?	2	3	0	1	1	1	2	0	0	0	0
Attenborosaurus	?	?	?	?	?	?	1	0	1	?	?	?	?	?	?	?
Peloneustes	1	0	1	1	?	0	2	1	1	1	0	2	0	1	0	0
Eoplesiosaurus	?	?	?	?	?	?	?	?	?	?	?	?	?	?	?	?
Plesiosaurus	?	?	?	?	?	?	1	0	0	1	1	1	?	?	?	0
Eretmosaurus	?	?	?	?	?	?	?	?	?	?	?	?	?	?	?	?
Westphaliasaurus	?	?	?	?	?	?	?	?	?	?	?	?	?	?	?	?
Seelyosaurus	?	?	?	?	?	?	1	0	0	1	1	0	?	?	?	?
M.tournemirensis	?	?	?	?	?	?	1	1	0	1	1	0	1	?	?	?
M. brachypterygius	?	?	?	?	?	?	?	0	0	1	1	0	1	?	?	?
M. homalospondylus	1	1	2	0	1	?	1	0	0	?	?	0	1	?	?	?
Plesiospterys	0	?	2	0	?	0	?	0	0	1	1	1	?	?	?	?
Cryptoclidus	0	1	2	0	1	0	2	0	0	1	1	1	1	1	1	1

	113	114	115	116	117	118	119	120	121	122	123	124	125	126	127	128
Yunguisaurus	?	?	2	0	0	0	?	?	0	0	?	0	0	0	0	0
***Pistosaurus* [postcranium]**	?	?	?	1	0	0	1	1	0	0	0	0	0	0	0	0
***Pistosaurus* [skull]**	?	?	?	?	?	?	?	?	?	?	?	?	?	?	?	?
Augustasaurus	2	?	2	0	?	?	?	?	0	0	0	0	0	2	0	0
Bobosaurus	0	?	3	2	0	0	0	?	?	1	1	?	1	2	?	0
**NHMUK_49202**	0	0	?	0	0	0	0	?	?	2	?	?	1	2	0	1
Stratesaurus	2	0	?	0	0	0	0	0	1	1	1	0	1	2	0	1
Macroplata	2	0	1	2	0	0	?	?	?	1	1	?	1	2	0	1
Avalonnectes	?	?	1	0	0	0	0	0	2	1	1	0	1	?	?	1
Eurycleidus	?	?	?	0	0	0	0	0	2	1	1	0	0	2	0	1
Meyerasaurus	?	?	1	2	0	?	?	?	?	?	?	0	?	2	0	?
Maresaurus	?	?	?	2	?	0	?	?	?	1	?	?	{01}	?	?	1
'R.' megacephalus	?	0	1	2	0	0	?	?	?	1	1	?	?	2	0	1
Archaeonectrus	?	?	1	0	0	0	0	0	1	1	1	0	0	2	0	1
R. cramptoni	?	?	1	2	?	0	?	1	2	1	1	0	0	?	?	1
R. zetlandicus	?	?	1	2	?	0	0	?	?	1	1	?	0	2	0	1
R. thorntoni	?	?	?	2	0	0	?	?	?	1	1	?	0	0	0	1
Thalassiodracon	2	0	0	0	0	0	0	0	1	1	1	0	1	2	0	1
H. longirostris	?	?	?	?	?	?	?	?	?	?	?	?	?	?	?	?
H. tomistomimus	{02}	1	0	0	0	0	?	1	1	1	1	?	1	0	0	1
H. zanoni	?	?	0	0	0	0	1	?	1	1	1	0	1	0	0	1
Attenborosaurus	?	?	0	0	?	0	0	1	1	1	1	?	?	?	?	1
Peloneustes	2	?	3	2	1	0	1	1	2	1	2	1	1	1	0	1
Eoplesiosaurus	?	?	2	0	?	0	0	1	1	1	?	?	1	2	?	1
Plesiosaurus	?	0	2	0	0	0	0	1	2	1	{01}	0	1	0	0	1
Eretmosaurus	?	?	{02}	0	0	0	1	0	?	1	0	?	1	2	0	1
Westphaliasaurus	?	?	{02}	?	0	0	1	0	2	1	?	?	?	0	0	1
Seelyosaurus	?	?	0	0	0	0	1	2	2	1	0	0	?	?	?	1
M.tournemirensis	?	?	2	1	0	1	1	1	2	1	0	?	2	0	?	1
M. brachypterygius	?	?	0	1	?	1	1	1	2	1	?	0	?	?	1	?
M. homalospondylus	?	?	2	1	1	1	1	1	2	1	0	0	2	0	1	1
Plesiospterys	?	0	2	1	?	0	1	1	2	1	1	2	?	0	0	1
Cryptoclidus	1	0	0	0	0	0	1	1	2	2	2	2	1	0	0	1

	129	130	131	132	133	134	135	136	137	138	139	140	141	142	143	144
Yunguisaurus	?	0	1	?	?	?	?	0	1	2	1	0	?	0	?	?
***Pistosaurus* [postcranium]**	0	0	1	0	1	0	1	0	1	1	0	0	0	1	0	0
***Pistosaurus* [skull]**	?	?	?	?	?	?	?	?	?	?	?	?	?	?	?	?
Augustasaurus	?	0	1	0	0	0	0	1	1	?	?	0	0	0	0	0
Bobosaurus	0	0	0	0	?	0	0	1	2	2	1	1	0	0	?	?
**NHMUK_49202**	0	0	0	3	1	1	?	?	?	?	?	?	?	?	?	?
Stratesaurus	0	0	0	3	1	1	?	?	1	?	?	?	?	0	?	0
Macroplata	0	0	?	3	0	0	0	1	?	1	1	1	0	0	0	0
Avalonnectes	0	0	0	1	1	0	0	1	1	0	1	1	0	1	1	2
Eurycleidus	0	0	0	1	0	0	0	?	1	?	1	1	0	1	1	?
Meyerasaurus	?	?	0	?	?	0	0	1	1	2	1	1	0	1	1	2
Maresaurus	?	?	0	3	0	?	?	?	?	?	?	?	?	?	?	?
'R.' megacephalus	?	?	?	1	0	0	0	1	1	1	?	1	0	1	1	2
Archaeonectrus	?	?	0	?	1	?	0	1	1	1	1	1	0	1	0	1
R. cramptoni	?	0	0	1	?	?	0	0	0	2	1	1	0	?	0	1
R. zetlandicus	0	0	0	1	0	0	0	?	?	2	1	1	0	?	0	1
R. thorntoni	0	0	?	1	0	?	0	1	?	2	1	1	0	1	?	?
Thalassiodracon	0	0	0	2	1	0	0	1	1	1	0	1	0	0	0	0
H. longirostris	?	?	?	?	?	?	?	?	?	?	?	?	?	?	?	?
H. tomistomimus	0	0	0	2	0	0	0	1	1	1	0	1	0	1	0	0
H. zanoni	0	0	0	2	0	0	0	?	?	?	?	?	?	?	?	?
Attenborosaurus	?	?	0	{03}	0	?	?	1	1	1	0	1	1	0	0	0
Peloneustes	0	0	0	0	0	1	0	0	1	1	1	1	1	0	0	0
Eoplesiosaurus	?	?	0	3	?	0	?	?	?	?	?	?	?	?	?	?
Plesiosaurus	1	1	0	3	{01}	0	0	1	1	{01}	1	1	1	0	0	0
Eretmosaurus	0	0	0	?	0	0	1	1	1	?	?	1	1	?	0	0
Westphaliasaurus	0	?	0	3	1	0	0	1	1	1	?	1	?	0	0	0
Seelyosaurus	?	?	0	0	?	0	?	1	2	0	0	1	1	0	0	0
M.tournemirensis	0	0	0	0	1	0	1	1	2	0	0	1	1	1	0	0
M. brachypterygius	?	?	0	0	1	0	?	1	2	0	1	1	1	1	0	0
M. homalospondylus	0	0	0	0	1	0	1	1	2	0	1	1	1	1	0	0
Plesiospterys	?	?	0	0	1	0	1	1	1	1	{01}	1	1	0	?	0
Cryptoclidus	2	1	0	0	0	0	0	1	1	1	0	1	1	0	0	0

	145	146	147	148	149	150	151	152	153	154	155	156	157	158	159	160
Yunguisaurus	0	0	0	?	?	?	0	?	?	?	0	?	0	0	0	?
***Pistosaurus* [postcranium]**	?	?	?	?	?	2	0	1	0	0	0	?	0	0	1	?
***Pistosaurus* [skull]**	?	?	?	?	?	?	?	?	?	?	?	?	?	?	?	?
Augustasaurus	?	?	?	?	?	?	?	?	?	0	0	?	0	0	0	?
Bobosaurus	0	1	0	1	1	?	0	?	?	?	?	?	?	?	?	?
**NHMUK_49202**	?	?	?	?	?	?	?	?	?	?	?	?	?	?	?	?
Stratesaurus	?	?	?	?	?	?	?	?	?	?	?	?	1	?	?	?
Macroplata	0	0	0	?	0	2	1	0	0	1	0	1	1	0	?	1
Avalonnectes	?	0	0	?	0	2	1	0	0	?	?	?	1	?	0	1
Eurycleidus	?	?	?	?	0	2	1	0	0	1	0	1	1	0	1	1
Meyerasaurus	0	0	0	1	?	2	1	0	?	0	1	1	?	0	?	1
Maresaurus	?	?	?	?	?	?	?	?	?	?	?	?	?	?	?	?
'R.' megacephalus	0	0	0	0	?	?	2	0	?	?	?	?	?	?	?	?
Archaeonectrus	0	0	0	0	0	?	?	?	?	?	?	?	?	?	0	?
R. cramptoni	0	0	0	?	0	?	?	0	?	?	?	?	1	?	?	?
R. zetlandicus	?	?	?	?	0	2	2	0	0	?	?	?	?	?	?	?
R. thorntoni	?	0	0	?	0	2	1	0	0	?	0	1	1	0	0	1
Thalassiodracon	0	0	0	0	0	2	1	0	0	1	0	1	1	0	1	1
H. longirostris	?	?	?	?	?	?	?	?	?	?	?	?	?	?	?	?
H. tomistomimus	1	0	0	0	?	?	1	?	?	?	?	?	1	?	?	1
H. zanoni	?	?	0	?	0	2	1	0	0	1	0	1	1	0	1	1
Attenborosaurus	1	?	0	?	?	?	1	?	1	1	0	?	1	0	?	1
Peloneustes	?	?	1	?	0	1	1	0	1	0	0	?	1	0	1	1
Eoplesiosaurus	?	?	0	0	0	0	0	1	?	1	?	?	1	0	0	1
Plesiosaurus	0	0	0	0	0	0	0	0	{01}	1	1	1	1	0	1	1
Eretmosaurus	?	?	?	?	0	0	0	0	1	?	?	?	1	?	?	?
Westphaliasaurus	0	1	0	?	0	0	0	0	1	1	1	1	1	0	0	1
Seelyosaurus	1	1	0	2	1	0	0	1	1	0	1	0	1	0	0	1
M.tournemirensis	0	1	0	?	?	?	?	?	?	1	0	1	1	0	0	1
M. brachypterygius	?	?	0	2	1	0	0	1	?	1	1	0	?	?	?	1
M. homalospondylus	0	1	0	?	1	0	0	1	1	1	1	0	1	1	1	1
Plesiospterys	0	0	0	2	0	0	0	0	?	1	?	1	1	?	0	1
Cryptoclidus	0	0	0	?	0	0	1	0	1	1	1	0	1	1	0	0

	161	162	163	164	165	166	167	168	169	170	171	172	173	174	175	176
Yunguisaurus	2	?	?	?	?	?	?	?	?	?	0	0	1	2	0	0
***Pistosaurus* [postcranium]**	2	1	0	0	?	?	?	0	1	?	?	0	1	2	0	2
***Pistosaurus* [skull]**	?	?	?	?	?	?	?	?	?	?	?	?	?	?	?	?
Augustasaurus	1	?	0	2	0	0	0	0	?	?	?	?	?	?	?	?
Bobosaurus	?	?	?	?	?	?	?	?	?	?	?	0	1	2	0	0
**NHMUK_49202**	?	?	?	?	?	?	?	?	?	?	?	?	?	?	?	?
Stratesaurus	?	?	?	?	?	?	?	?	?	?	?	0	1	0	0	2
Macroplata	?	?	0	0	0	0	0	?	0	2	0	0	1	?	0	0
Avalonnectes	2	?	?	?	?	?	?	?	?	?	?	0	1	0	0	?
Eurycleidus	2	1	0	2	0	1	0	1	0	2	0	0	1	0	0	0
Meyerasaurus	?	?	0	2	0	1	0	?	0	0	0	0	1	0	0	0
Maresaurus	?	?	?	?	?	?	?	?	?	?	?	?	?	?	?	?
'R.' megacephalus	?	1	?	?	?	?	?	?	?	?	?	0	1	0	0	0
Archaeonectrus	2	1	?	?	?	?	?	?	?	?	?	0	1	?	0	?
R. cramptoni	?	?	?	?	?	?	?	1	?	?	?	0	1	0	0	?
R. zetlandicus	?	?	?	?	?	?	?	?	?	?	?	?	?	?	?	?
R. thorntoni	2	1	0	?	0	?	0	1	0	2	0	0	1	2	0	0
Thalassiodracon	0	?	0	0	0	0	0	1	0	{02}	0	0	1	0	0	0
H. longirostris	?	?	?	?	?	?	?	?	?	?	?	?	?	?	?	?
H. tomistomimus	?	0	?	?	?	?	?	?	?	2	0	?	?	?	?	?
H. zanoni	2	?	0	0	2	0	0	?	1	2	0	0	1	0	0	0
Attenborosaurus	?	?	0	2	2	0	0	?	0	3	1	0	?	0	0	?
Peloneustes	0	0	2	0	2	0	0	?	?	?	1	0	0	2	1	0
Eoplesiosaurus	0	?	0	?	0	?	0	?	?	?	?	0	?	?	0	?
Plesiosaurus	0	1	0	0	0	0	0	?	0	0	0	0	0	0	0	0
Eretmosaurus	?	?	{02}	?	0	?	0	?	?	?	0	0	1	0	0	0
Westphaliasaurus	2	?	0	0	0	0	0	1	?	2	0	0	?	?	0	0
Seelyosaurus	2	?	2	0	0	3	?	?	?	3	0	0	?	0	0	?
M.tournemirensis	2	1	2	0	1	3	1	?	1	3	0	0	1	2	0	0
M. brachypterygius	?	?	1	0	1	3	?	?	1	?	1	0	1	2	0	0
M. homalospondylus	0	1	?	0	1	?	1	1	1	?	0	0	1	2	0	0
Plesiospterys	2	?	?	0	?	?	?	?	1	3	?	0	1	0	0	0
Cryptoclidus	1	0	1	0	1	0	1	2	1	?	1	1	0	0	0	1

	177	178	179	180	181	182	183	184	185	186	187	188	189	190	191	192
Yunguisaurus	1	?	0	0	0	0	0	0	?	?	0	0	?	0	2	?
***Pistosaurus* [postcranium]**	0	?	?	?	1	?	?	0	1	?	0	0	0	0	2	0
***Pistosaurus* [skull]**	?	?	?	?	?	?	?	?	?	?	?	?	?	?	?	?
Augustasaurus	?	?	?	?	?	0	0	0	?	?	0	0	0	0	2	0
Bobosaurus	0	?	0	0	1	?	?	0	?	?	0	0	0	?	?	?
**NHMUK_49202**	?	?	?	?	?	?	?	?	?	?	?	?	?	?	?	?
Stratesaurus	1	1	?	?	?	?	?	?	?	?	?	?	?	?	?	?
Macroplata	2	?	0	0	2	?	?	?	1	0	?	0	?	0	2	?
Avalonnectes	1	0	?	?	1	?	?	?	?	?	1	0	?	0	2	?
Eurycleidus	1	0	0	0	1	?	?	0	1	0	1	1	1	0	0	0
Meyerasaurus	1	?	0	0	1	1	0	0	?	?	1	0	?	0	2	1
Maresaurus	?	?	?	?	?	?	?	?	?	?	?	?	?	?	?	?
'R.' megacephalus	1	0	?	?	?	?	?	0	?	?	1	0	1	0	0	?
Archaeonectrus	1	0	0	?	1	0	0	0	?	?	1	0	?	0	?	?
R. cramptoni	1	0	0	0	?	1	?	0	?	?	1	1	?	0	0	?
R. zetlandicus	?	?	?	?	?	1	0	0	1	2	1	1	0	0	0	1
R. thorntoni	1	0	0	0	1	?	?	0	1	1	1	1	0	0	2	1
Thalassiodracon	1	1	0	0	1	1	0	0	0	0	1	0	0	0	2	0
H. longirostris	?	?	?	?	?	?	?	?	?	?	?	?	?	?	?	?
H. tomistomimus	?	?	0	0	?	?	0	0	0	0	2	0	0	0	0	1
H. zanoni	2	?	0	0	0	1	0	0	?	?	2	0	?	0	0	1
Attenborosaurus	1	?	0	0	1	0	0	0	?	?	1	0	?	0	?	?
Peloneustes	1	1	1	0	2	0	0	1	?	?	1	1	0	0	0	1
Eoplesiosaurus	?	?	0	0	1	1	0	0	0	?	2	0	1	0	?	?
Plesiosaurus	2	1	0	0	1	0	0	0	0	?	1	0	1	0	2	0
Eretmosaurus	2	0	0	0	1	1	0	0	?	?	1	1	?	1	2	0
Westphaliasaurus	2	0	0	0	1	?	?	0	0	1	1	1	0	0	0	2
Seelyosaurus	2	0	0	0	1	1	0	0	?	?	1	0	?	1	2	?
M.tournemirensis	2	0	0	0	1	1	?	0	?	?	1	0	?	1	2	?
M. brachypterygius	1	0	?	?	0	1	0	0	?	?	1	1	?	1	2	?
M. homalospondylus	2	0	0	0	0	1	?	0	0	0	1	1	?	1	2	0
Plesiospterys	2	?	0	0	1	?	?	0	0	0	1	0	0	1	0	?
Cryptoclidus	1	1	0	1	1	1	1	0	1	1	3	2	0	1	0	1

	193	194	195	196	197	198	199	200	201	202	203	204	205	206	207
Yunguisaurus	?	0	0	0	0	0	0	0	0	0	0	0	0	0	0
***Pistosaurus* [postcranium]**	?	0	?	?	?	0	?	?	0	?	?	?	?	?	0
***Pistosaurus* [skull]**	?	?	?	?	?	?	?	?	?	?	?	?	?	?	?
Augustasaurus	?	0	?	1	0	0	?	?	0	0	0	?	0	0	0
Bobosaurus	?	?	0	?	?	?	0	?	0	?	?	0	?	?	?
**NHMUK_49202**	?	?	?	?	?	?	?	?	?	?	?	?	?	?	?
Stratesaurus	?	?	1	?	?	?	0	1	0	?	?	0	?	1	0
Macroplata	0	?	1	?	?	?	0	1	0	?	?	0	?	?	?
Avalonnectes	0	1	1	0	?	1	0	1	0	0	0	0	1	0	0
Eurycleidus	0	?	?	?	?	1	?	1	0	?	?	?	?	?	0
Meyerasaurus	?	1	1	0	1	1	0	1	0	0	0	1	1	0	0
Maresaurus	?	?	?	?	?	?	?	?	?	?	?	?	?	?	?
'R.' megacephalus	?	?	1	?	?	1	0	1	0	?	?	0	?	?	0
Archaeonectrus	0	1	1	0	1	1	0	1	0	0	0	1	?	0	0
R. cramptoni	?	1	1	0	1	1	0	1	0	0	?	1	?	0	0
R. zetlandicus	0	1	1	0	1	1	0	?	0	0	1	?	1	0	?
R. thorntoni	0	?	?	?	?	?	?	?	?	?	?	?	?	?	?
Thalassiodracon	0	1	1	0	0	1	0	1	0	0	0	0	1	0	0
H. longirostris	?	?	?	?	?	?	?	?	?	?	?	?	?	?	?
H. tomistomimus	0	1	1	0	1	?	1	1	0	?	1	1	1	0	0
H. zanoni	?	1	1	0	1	1	0	1	0	0	0	1	1	0	0
Attenborosaurus	?	1	?	0	0	1	?	?	0	0	?	?	1	0	1
Peloneustes	0	2	2	1	0	1	1	1	0	1	1	1	1	1	1
Eoplesiosaurus	0	1	1	0	?	1	0	1	0	0	0	0	1	0	0
Plesiosaurus	0	1	1	0	{01}	1	0	1	0	0	0	0	1	0	0
Eretmosaurus	0	1	1	0	1	1	0	1	0	0	0	0	1	0	0
Westphaliasaurus	0	1	1	0	1	1	0	1	0	0	0	1	?	?	1
Seelyosaurus	0	1	1	0	1	1	0	1	0	0	1	1	1	0	1
M.tournemirensis	0	1	1	0	1	1	0	1	0	0	0	1	?	?	1
M. brachypterygius	?	1	1	0	1	0	0	1	0	0	1	1	1	0	1
M. homalospondylus	0	1	1	0	1	1	0	1	0	0	1	1	1	0	1
Plesiospterys	?	1	1	0	0	1	0	1	0	0	1	1	1	1	0
Cryptoclidus	1	1	2	0	0	1	1	1	2	2	0	0	2	1	1
